# Supplementary figures and images for: The intrinsically disordered cytoplasmic tail of a dendrite branching receptor uses two distinct mechanisms to regulate the actin cytoskeleton
Source: eLife. 2023 Aug 9;12:e88492. doi: 10.7554/eLife.88492 (PMC10411975; doi:10.7554/eLife.88492)

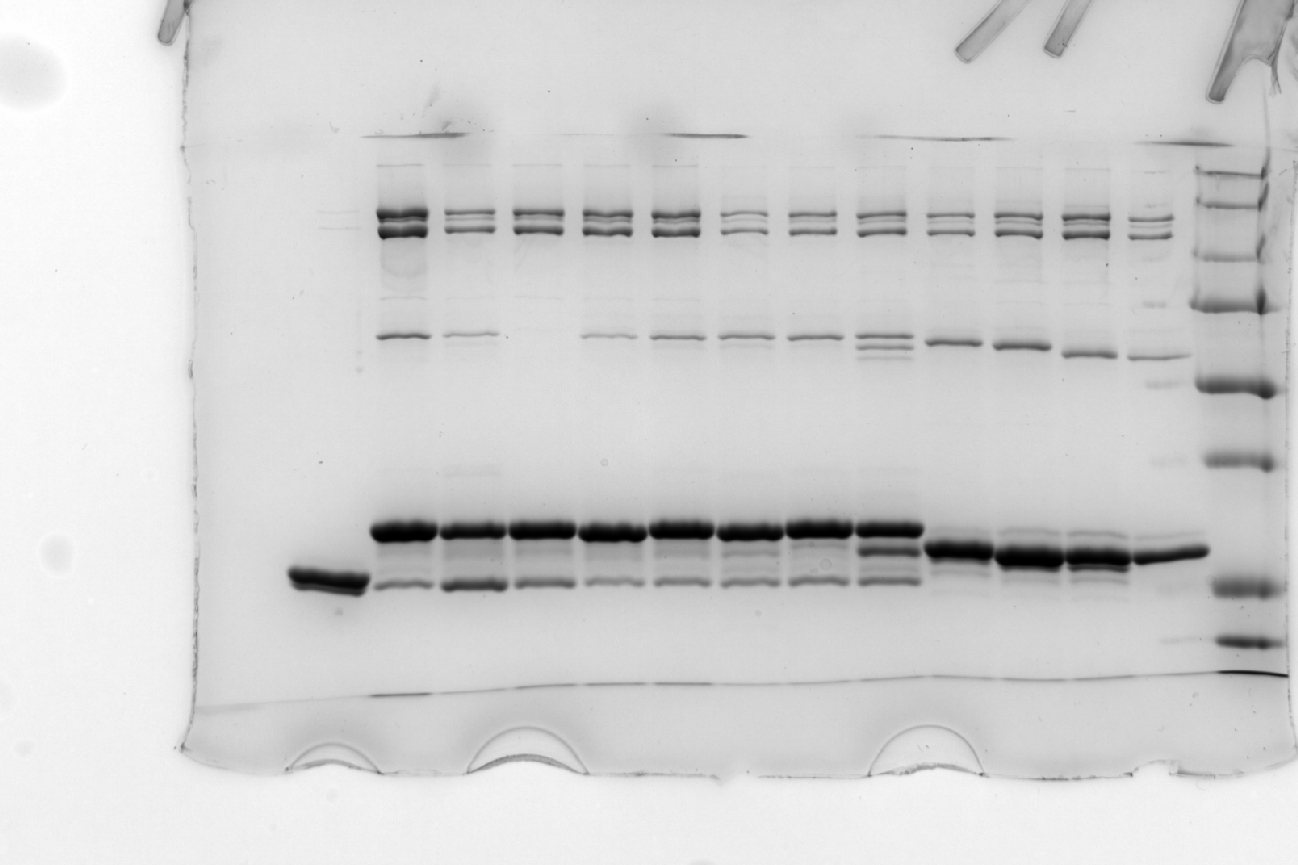

Supplement: Figure 1—source data 1. [file elife-88492-fig1-data1.zip › Figure 1 - source data 1/Figure 1B raw image.jpg]

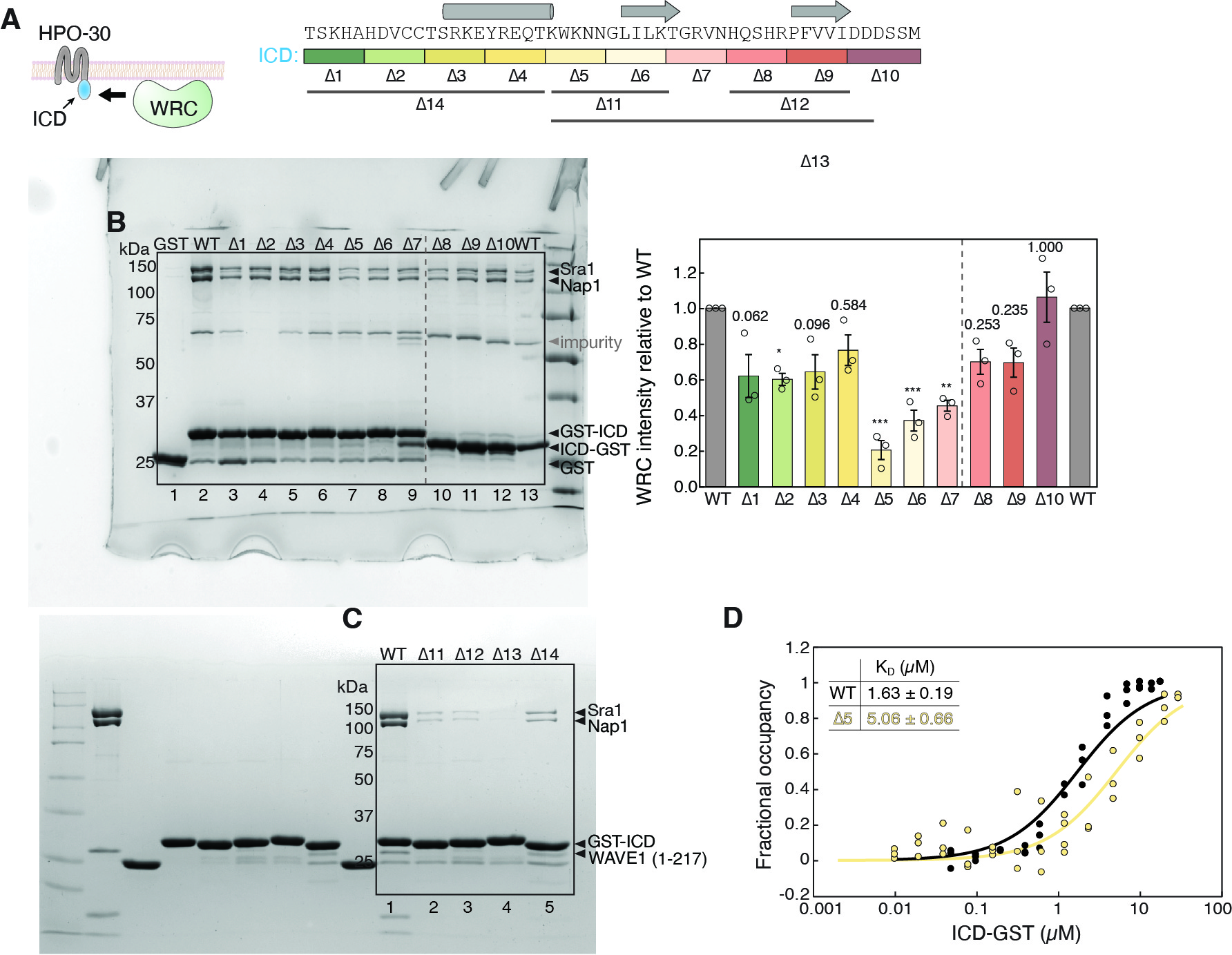

Supplement: Figure 1—source data 1. [file elife-88492-fig1-data1.zip › Figure 1 - source data 1/Figure 1 unedited.jpg]

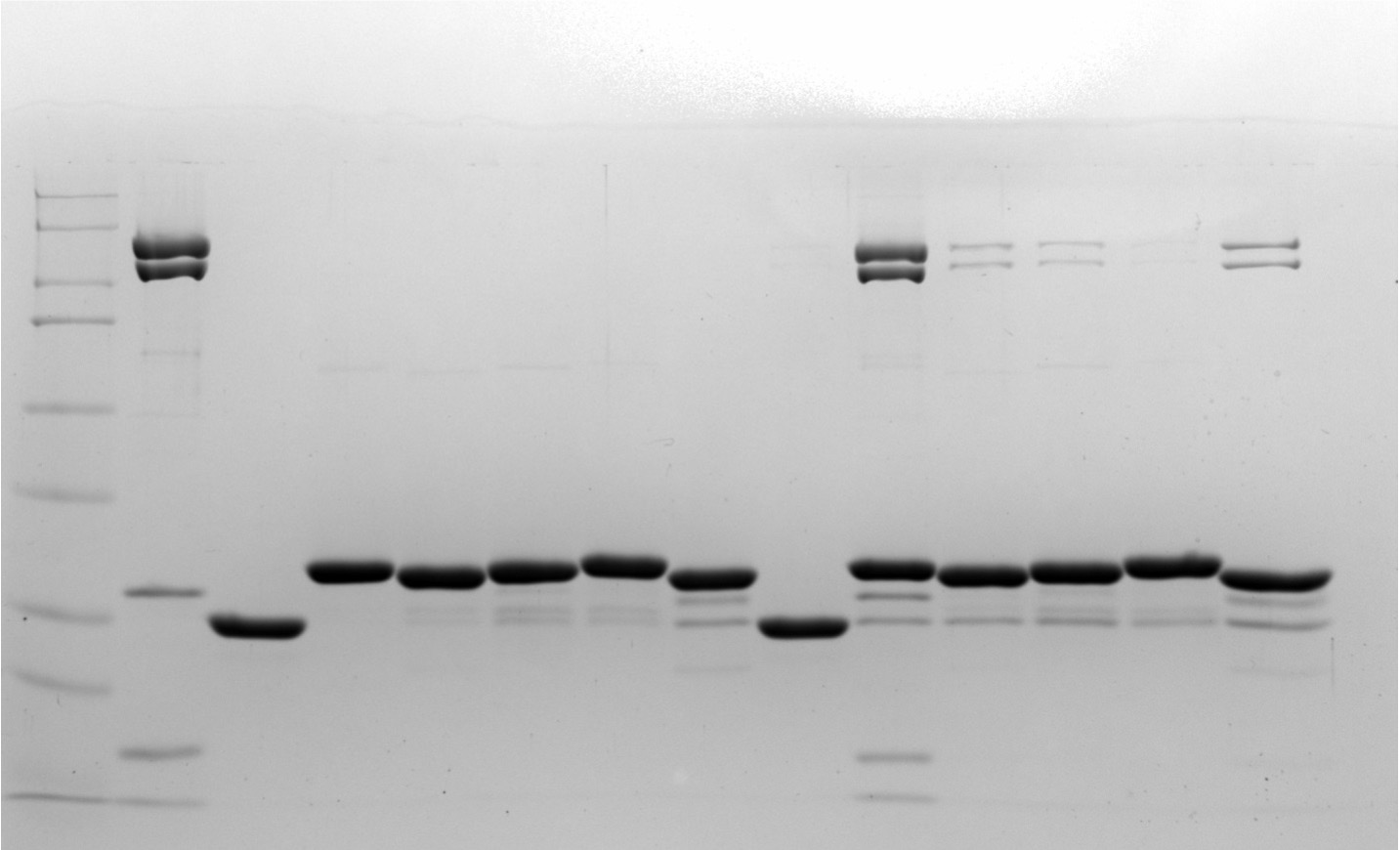

Supplement: Figure 1—source data 1. [file elife-88492-fig1-data1.zip › Figure 1 - source data 1/Figure 1C raw image.jpg]

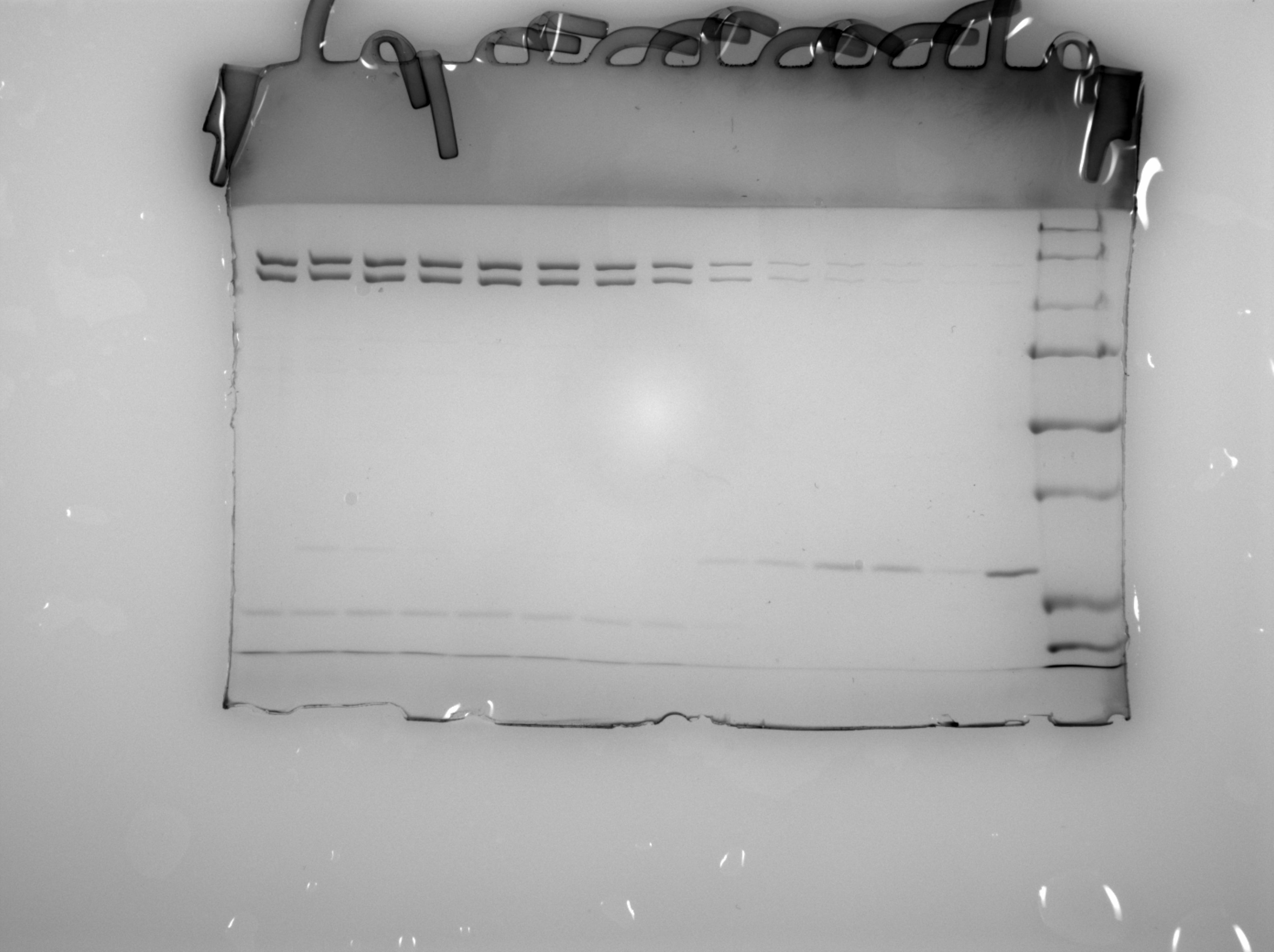

Supplement: Figure 1—figure supplement 1—source data 1. [file elife-88492-fig1-figsupp1-data1.zip › Figure 1 - figure supplement 1 - source data 1/Figure 1 - figure supplement 1A raw image WT.jpg]

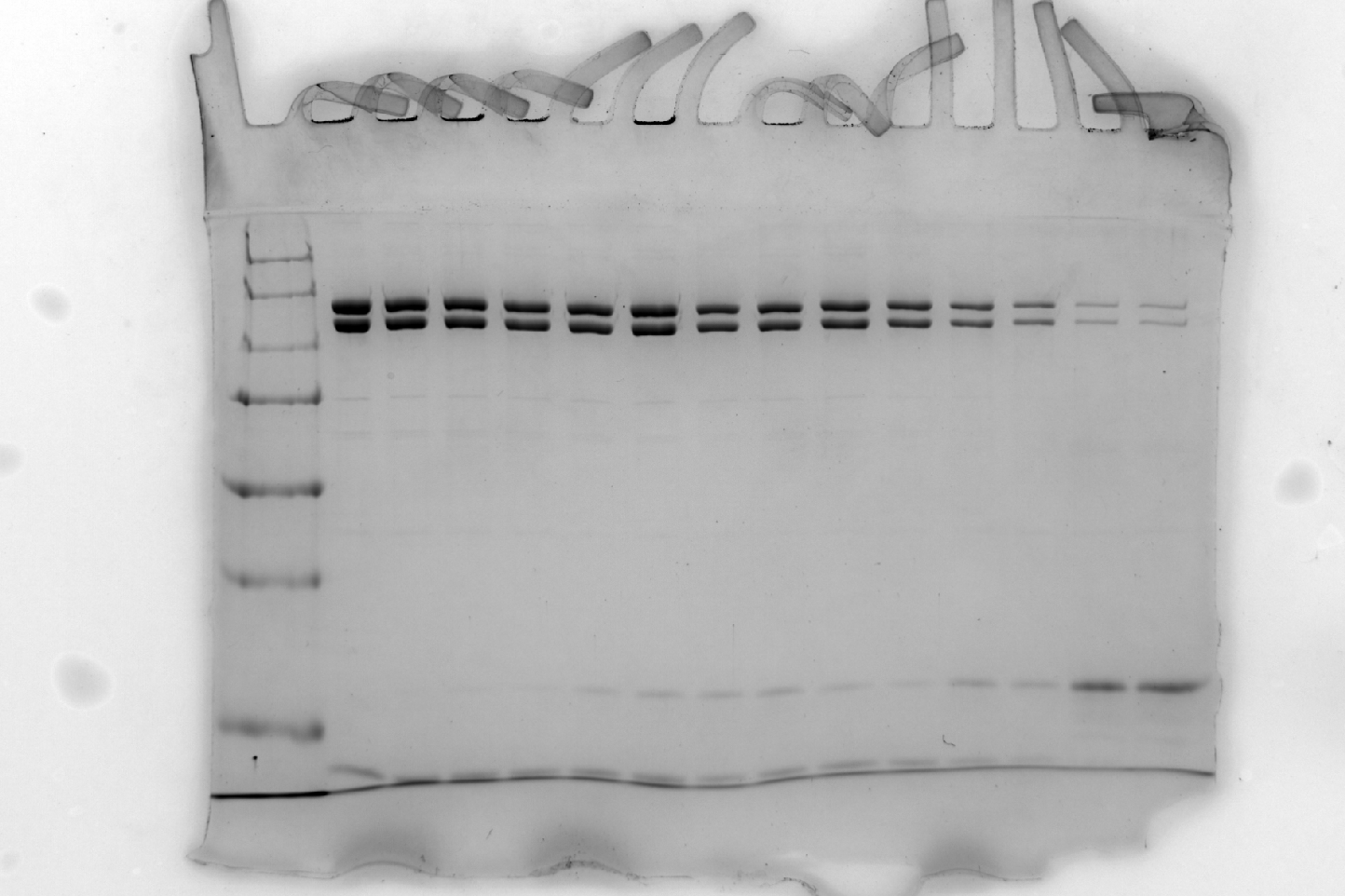

Supplement: Figure 1—figure supplement 1—source data 1. [file elife-88492-fig1-figsupp1-data1.zip › Figure 1 - figure supplement 1 - source data 1/Figure 1 - figure supplement 1B raw image Ala #5.jpg]

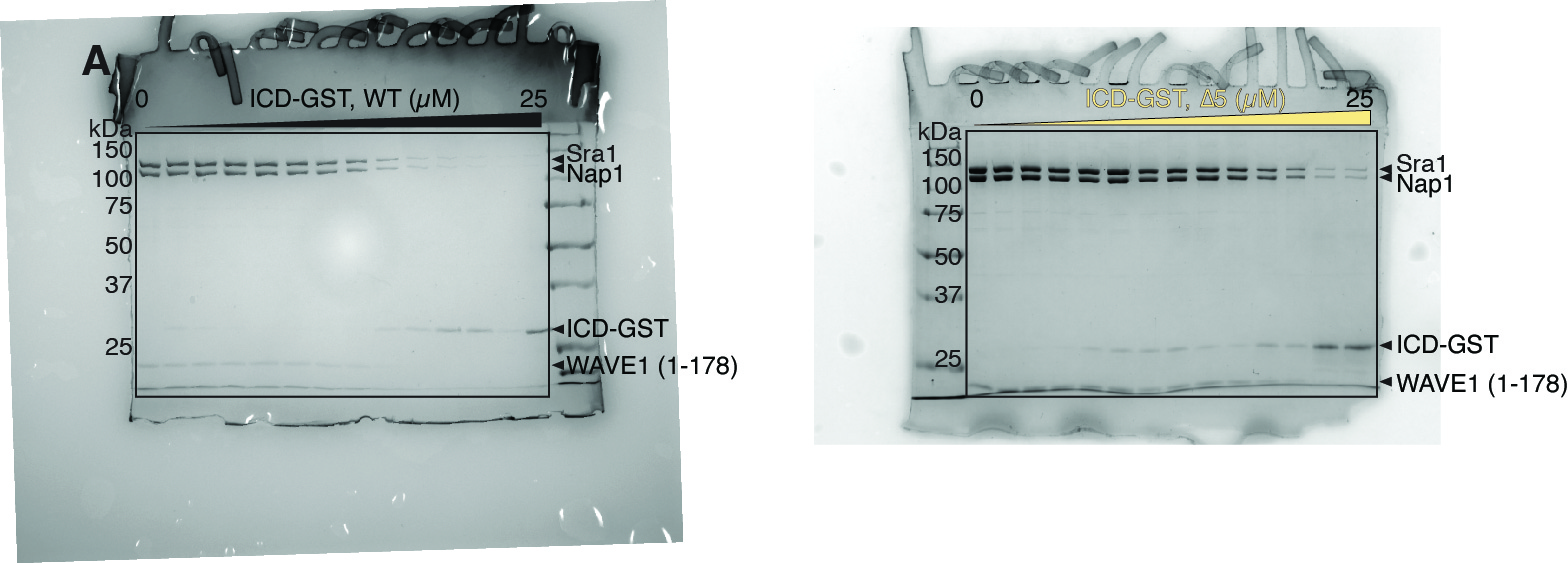

Supplement: Figure 1—figure supplement 1—source data 1. [file elife-88492-fig1-figsupp1-data1.zip › Figure 1 - figure supplement 1 - source data 1/Figure 1 - figure supplement 1- unedited.jpg]

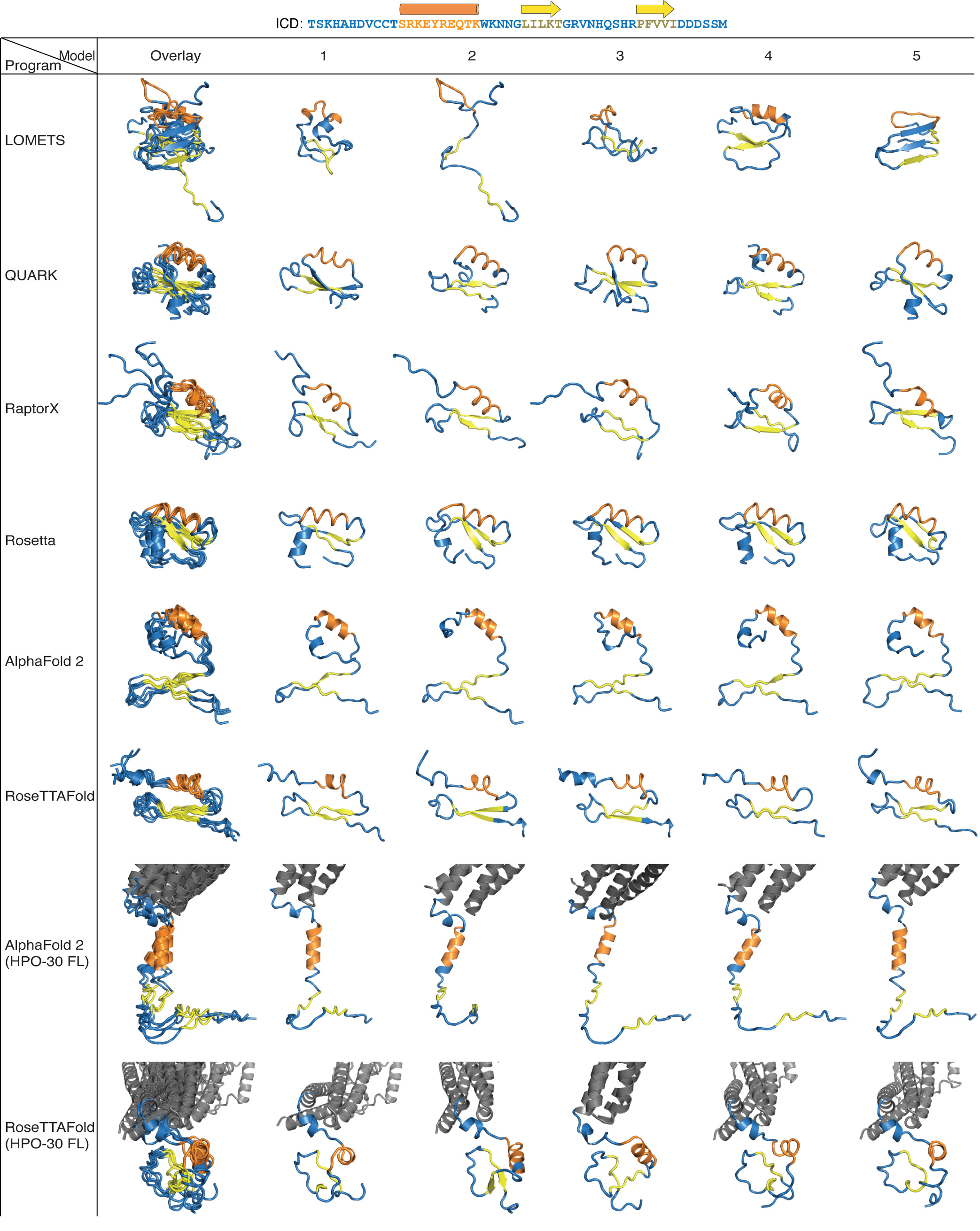

Supplement: Figure 1—figure supplement 2—source data 1. [file elife-88492-fig1-figsupp2-data1.zip › Figure 1 - figure supplement 2 - source data 1/Figure 1 - figure supplement 2 - unedited.jpg]

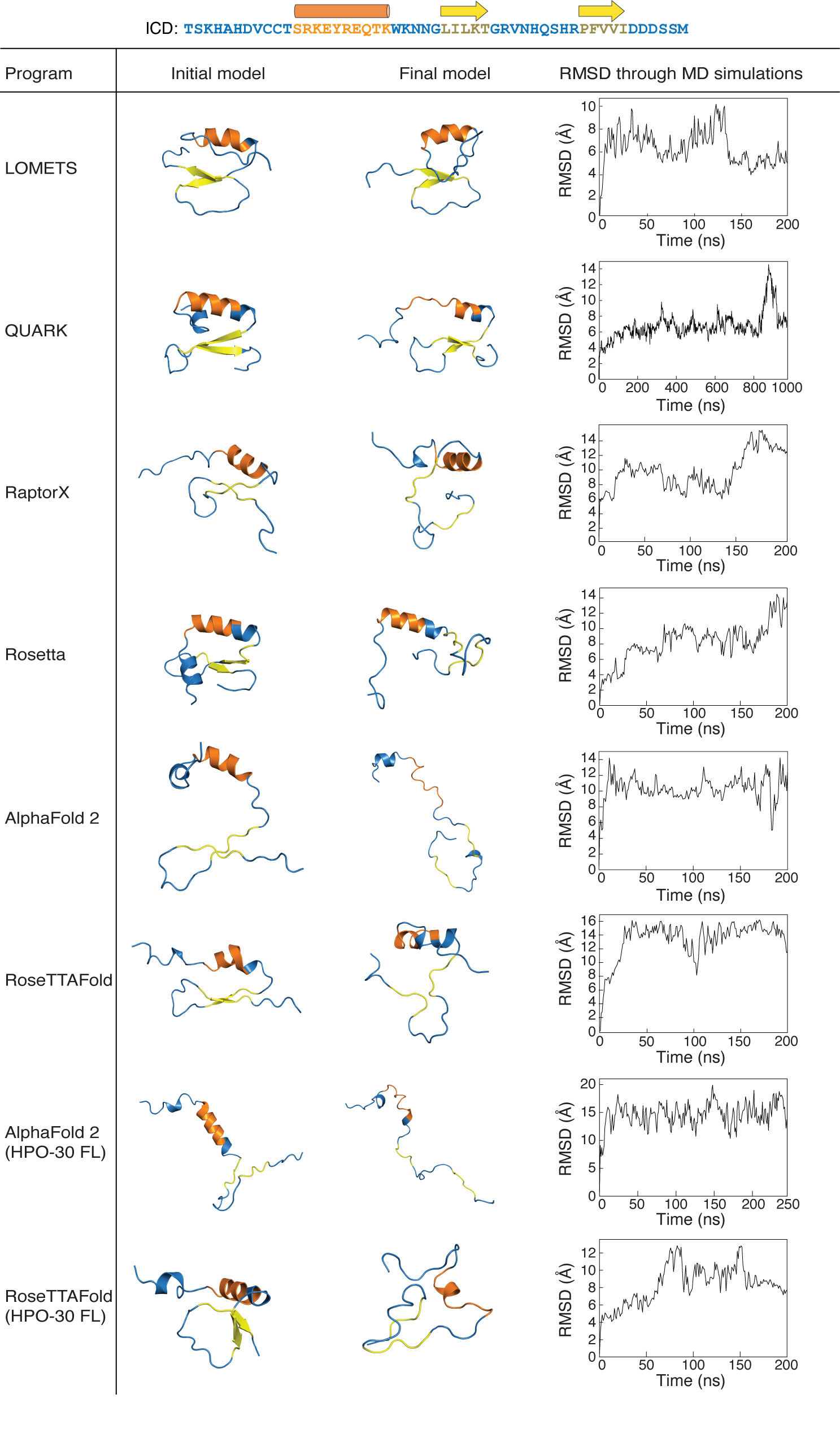

Supplement: Figure 1—figure supplement 3—source data 1. [file elife-88492-fig1-figsupp3-data1.zip › Figure 1 - figure supplement 3 - source data 1/Figure 1 - figure supplement 3 - unedited.jpg]

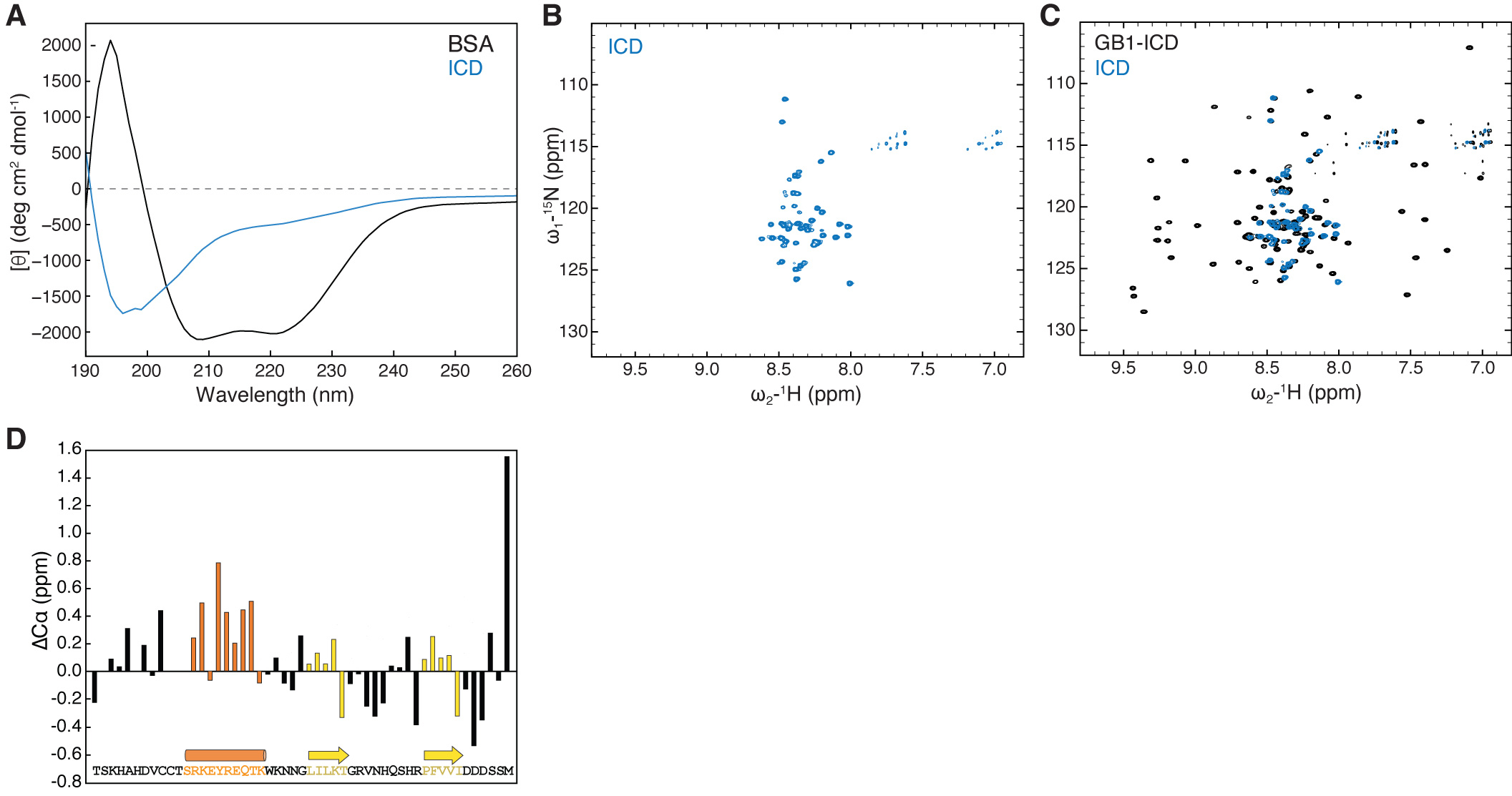

Supplement: Figure 2—source data 1. [file elife-88492-fig2-data1.zip › Figure 2 - source data 1/Figure 2 - unedited.jpg]

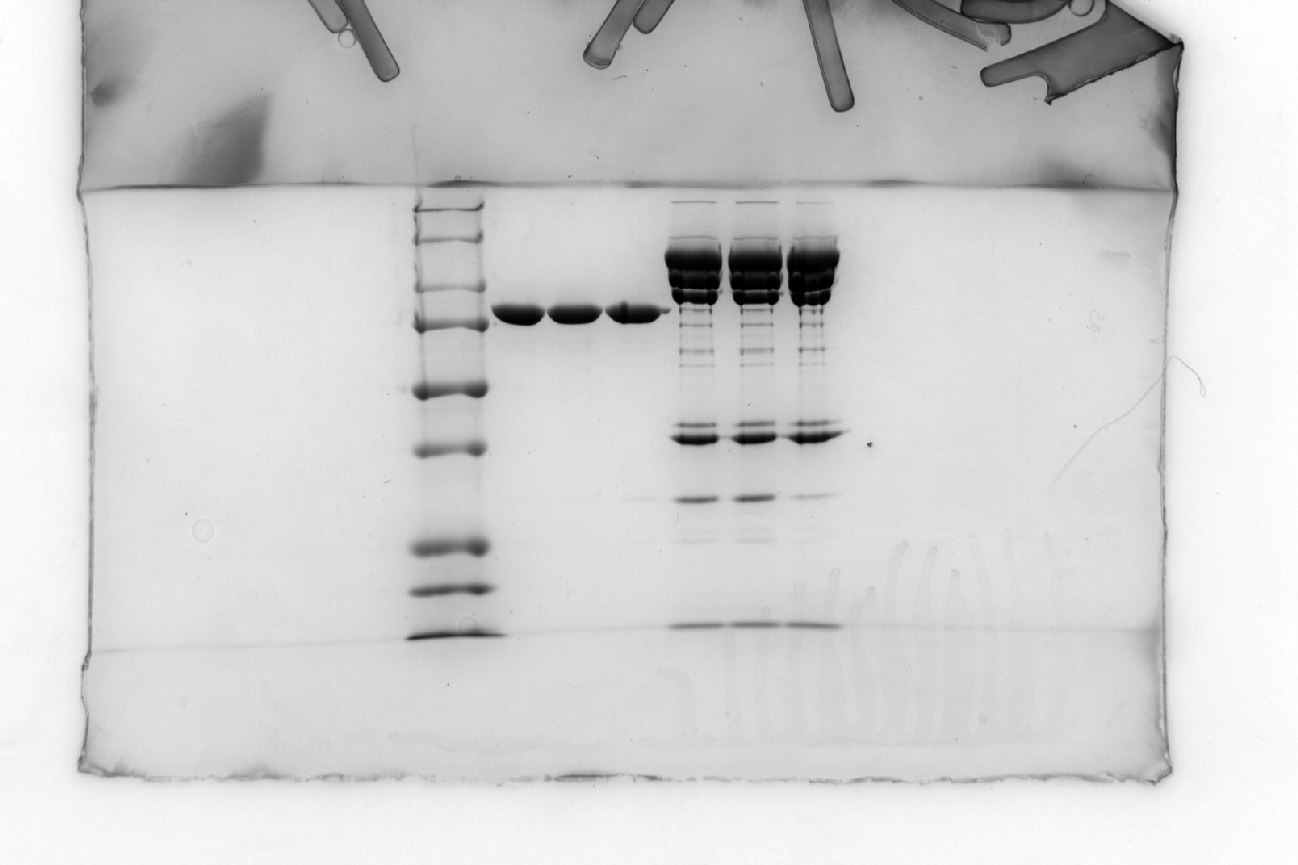

Supplement: Figure 2—figure supplement 1—source data 2. [file elife-88492-fig2-figsupp1-data2.zip › Figure 2 - figure supplement 1 - source data 2/Figure 2B right raw image.jpg]

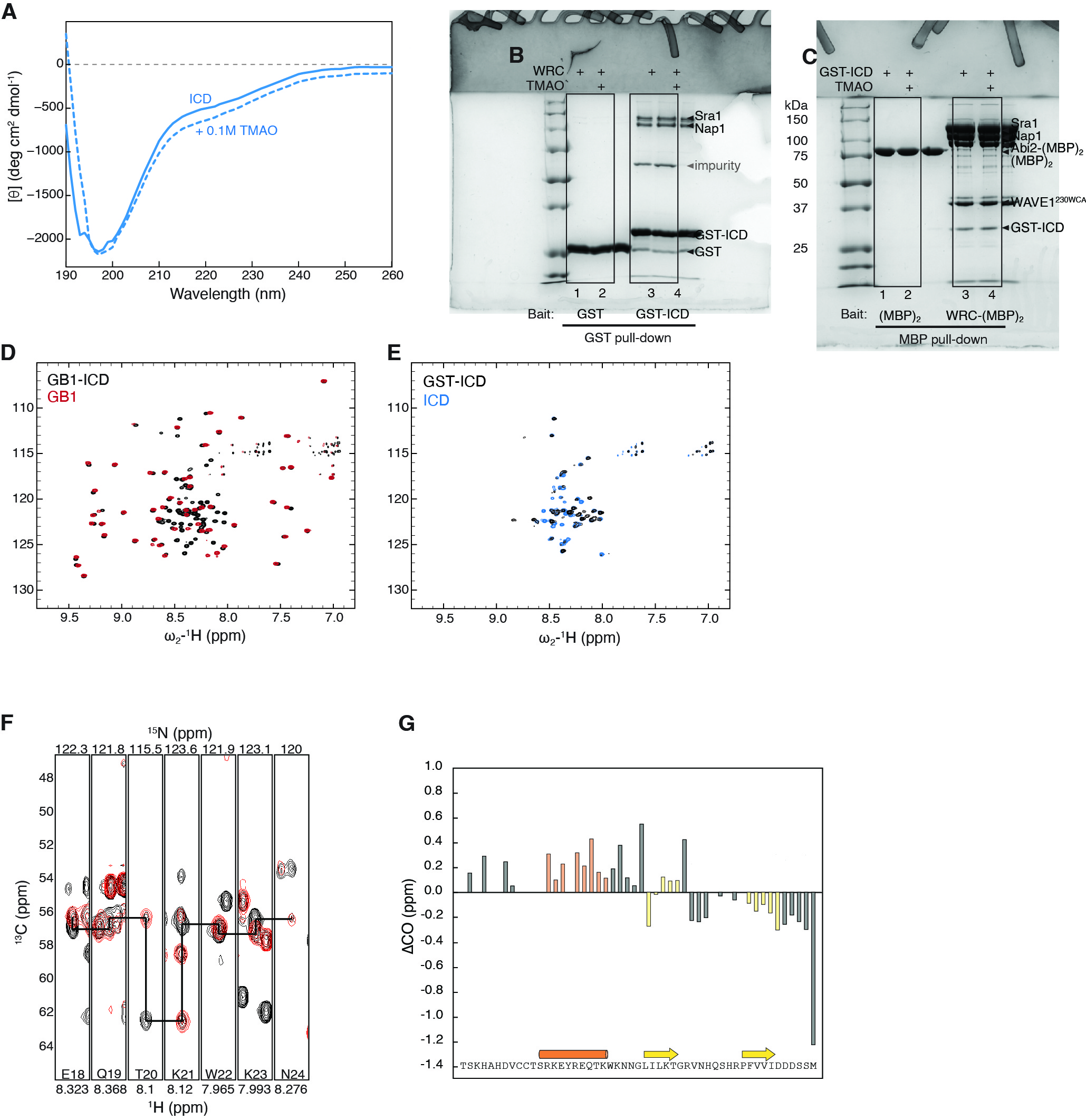

Supplement: Figure 2—figure supplement 1—source data 2. [file elife-88492-fig2-figsupp1-data2.zip › Figure 2 - figure supplement 1 - source data 2/Figure 2 - figure supplement 1 unedited.jpg]

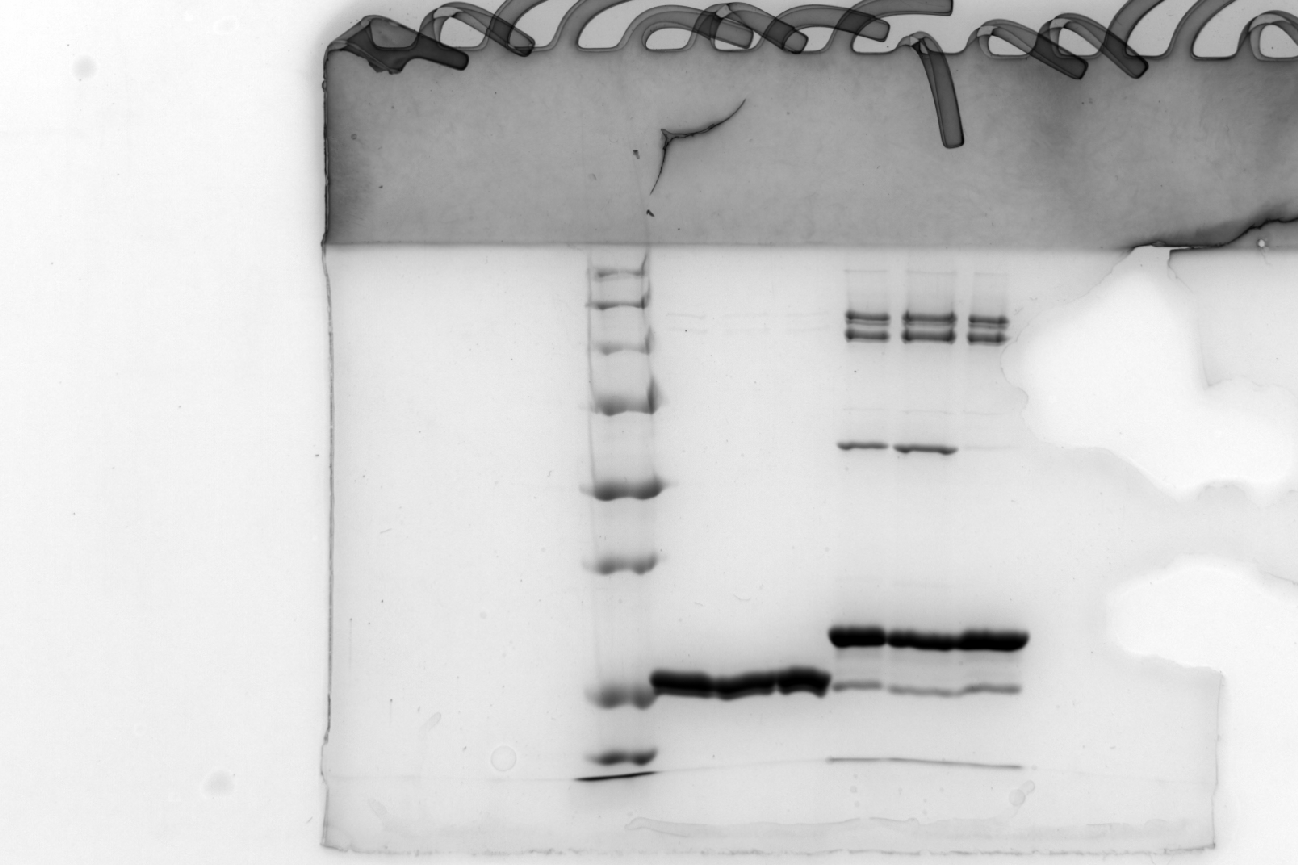

Supplement: Figure 2—figure supplement 1—source data 2. [file elife-88492-fig2-figsupp1-data2.zip › Figure 2 - figure supplement 1 - source data 2/Figure 2B left raw image.jpg]

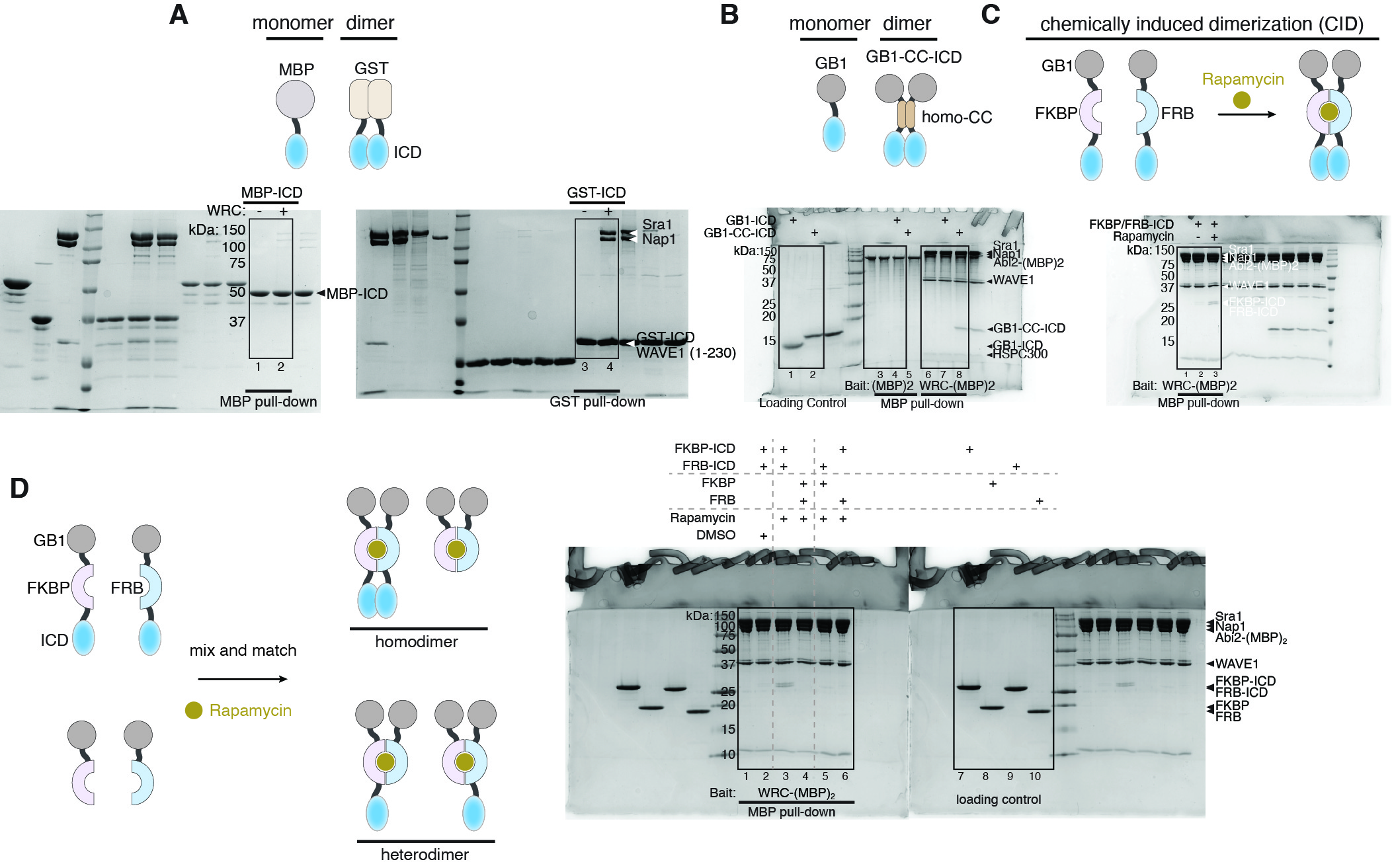

Supplement: Figure 3—source data 1. [file elife-88492-fig3-data1.zip › Figure 3 - source data 1/Figure 3 unedited.jpg]

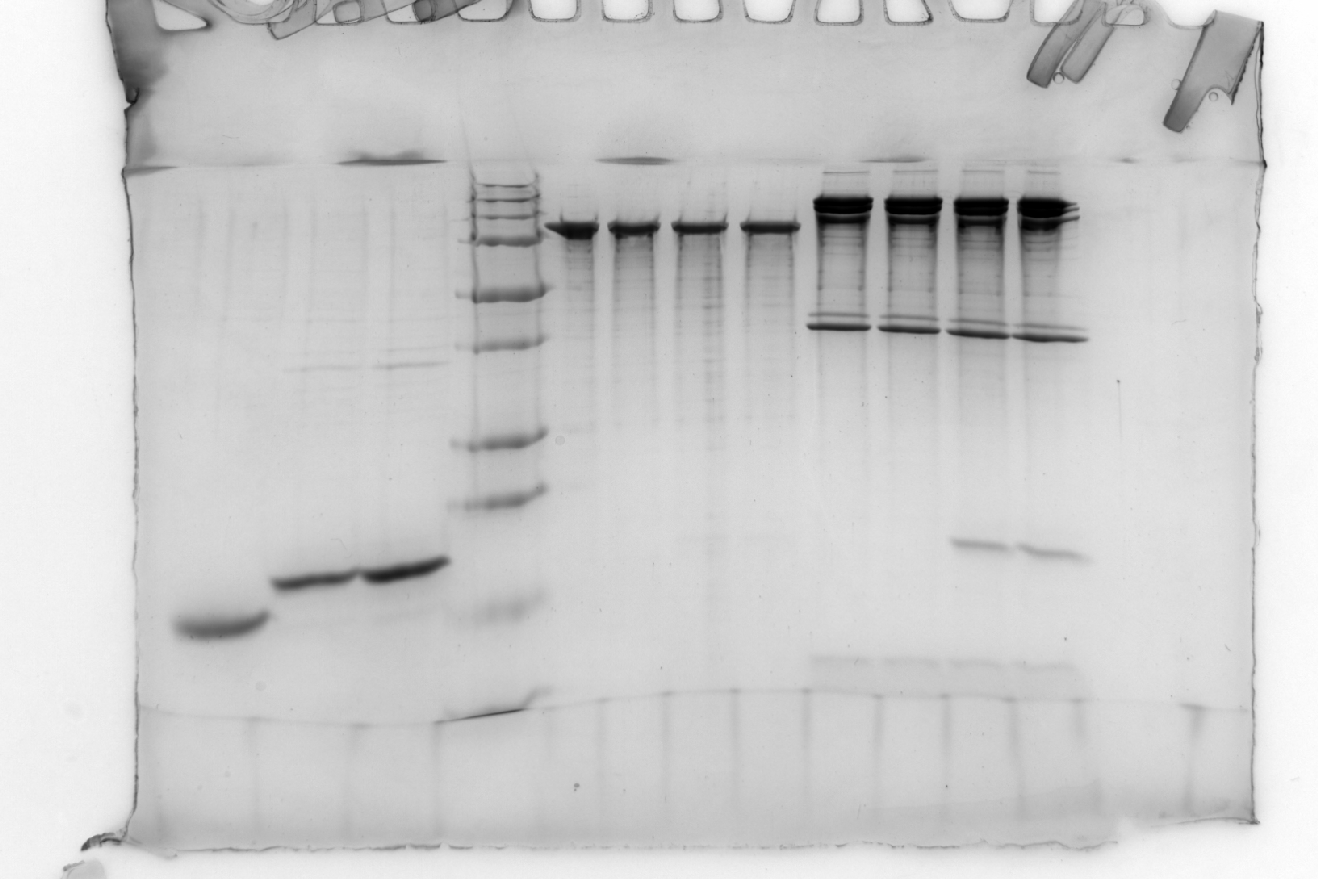

Supplement: Figure 3—source data 1. [file elife-88492-fig3-data1.zip › Figure 3 - source data 1/Figure 3B Raw image.jpg]

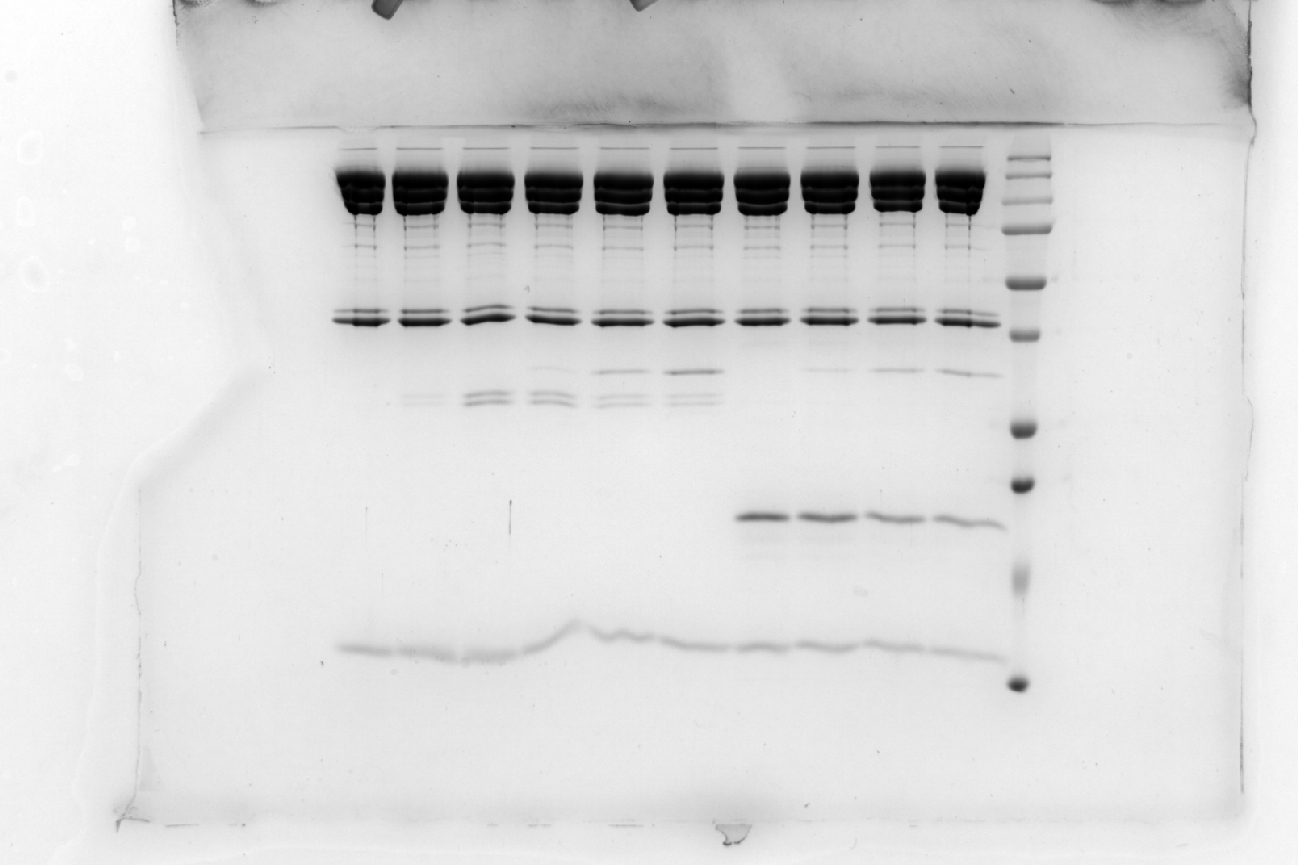

Supplement: Figure 3—source data 1. [file elife-88492-fig3-data1.zip › Figure 3 - source data 1/Figure 3C raw image.jpg]

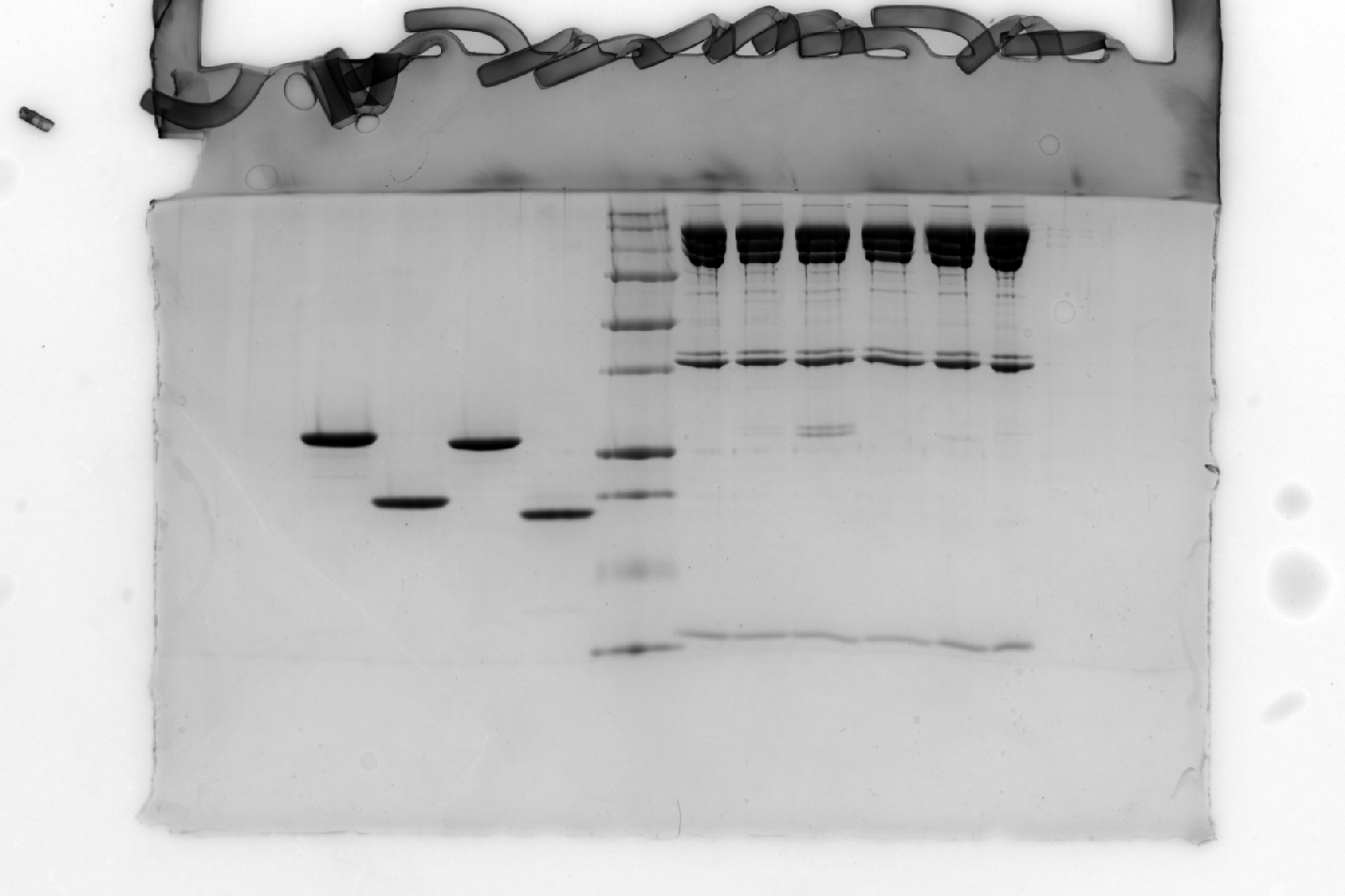

Supplement: Figure 3—source data 1. [file elife-88492-fig3-data1.zip › Figure 3 - source data 1/Figure 3D raw image.jpg]

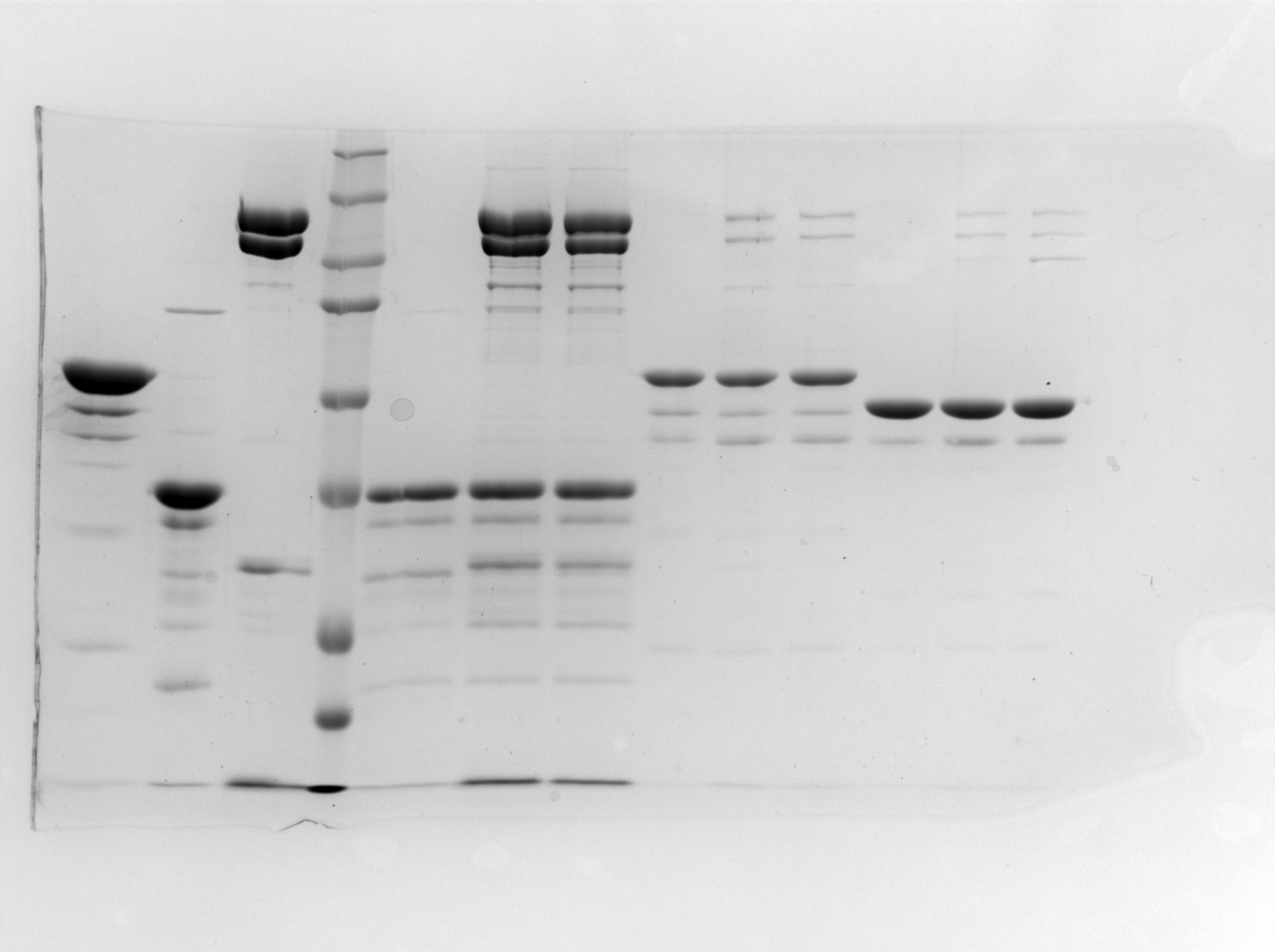

Supplement: Figure 3—source data 1. [file elife-88492-fig3-data1.zip › Figure 3 - source data 1/Figure 3A Left raw image.jpg]

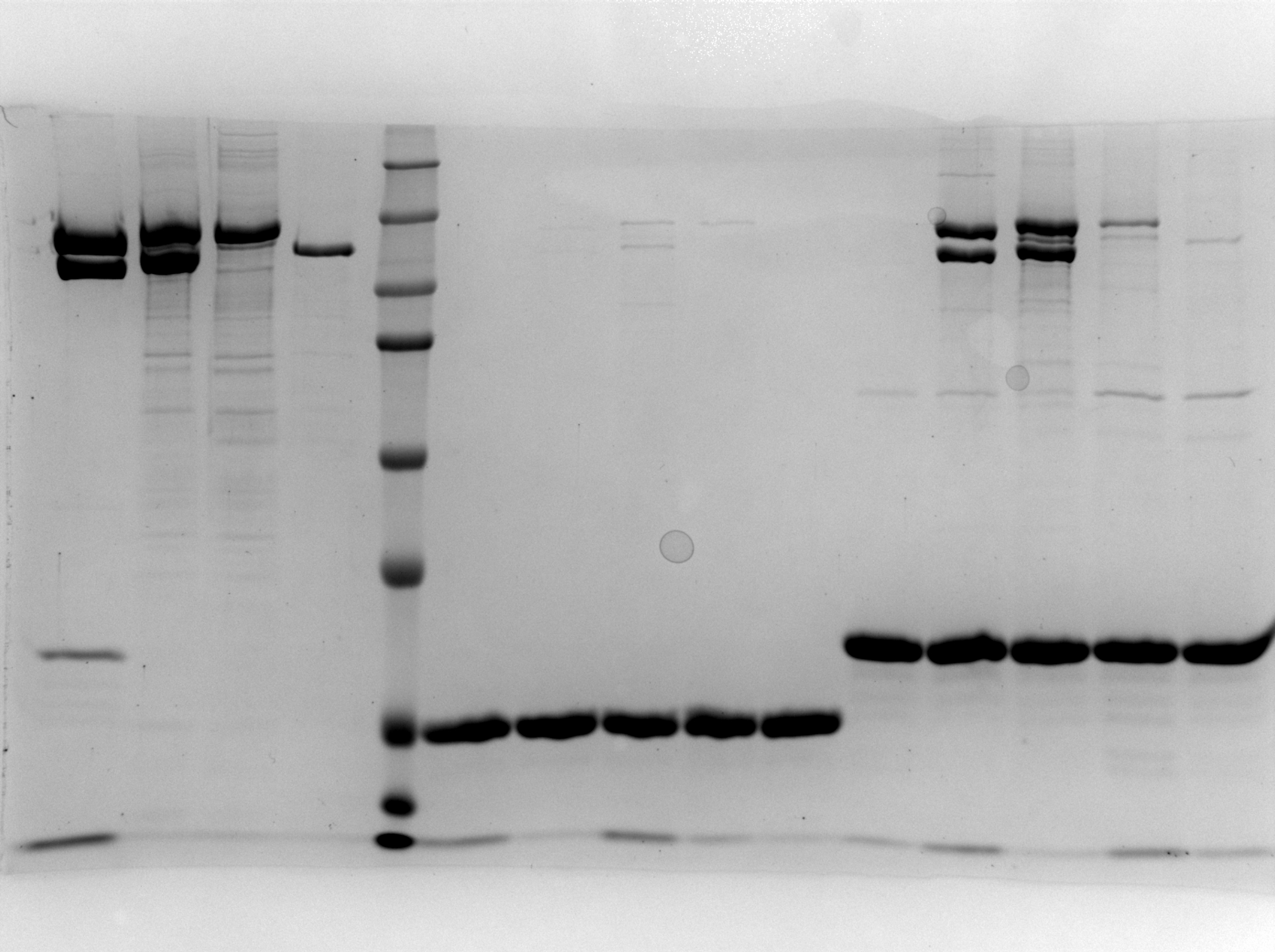

Supplement: Figure 3—source data 1. [file elife-88492-fig3-data1.zip › Figure 3 - source data 1/Figure 3A Right raw image.jpg]

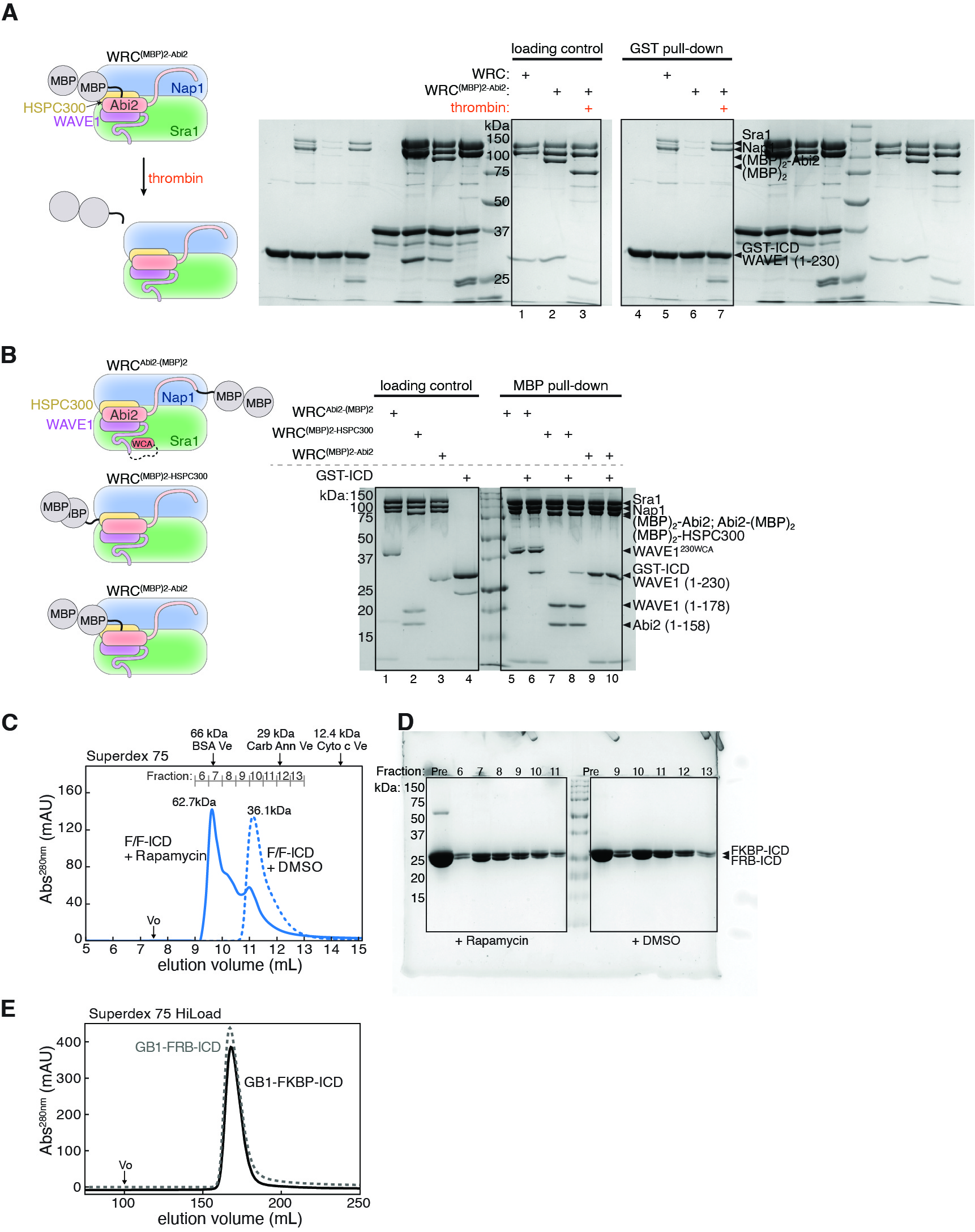

Supplement: Figure 3—figure supplement 1—source data 1. [file elife-88492-fig3-figsupp1-data1.zip › Figure 3 - figure supplement 1 - source data 1/Figure 3ΓÇöFigure supplement 1 Unedited.jpg]

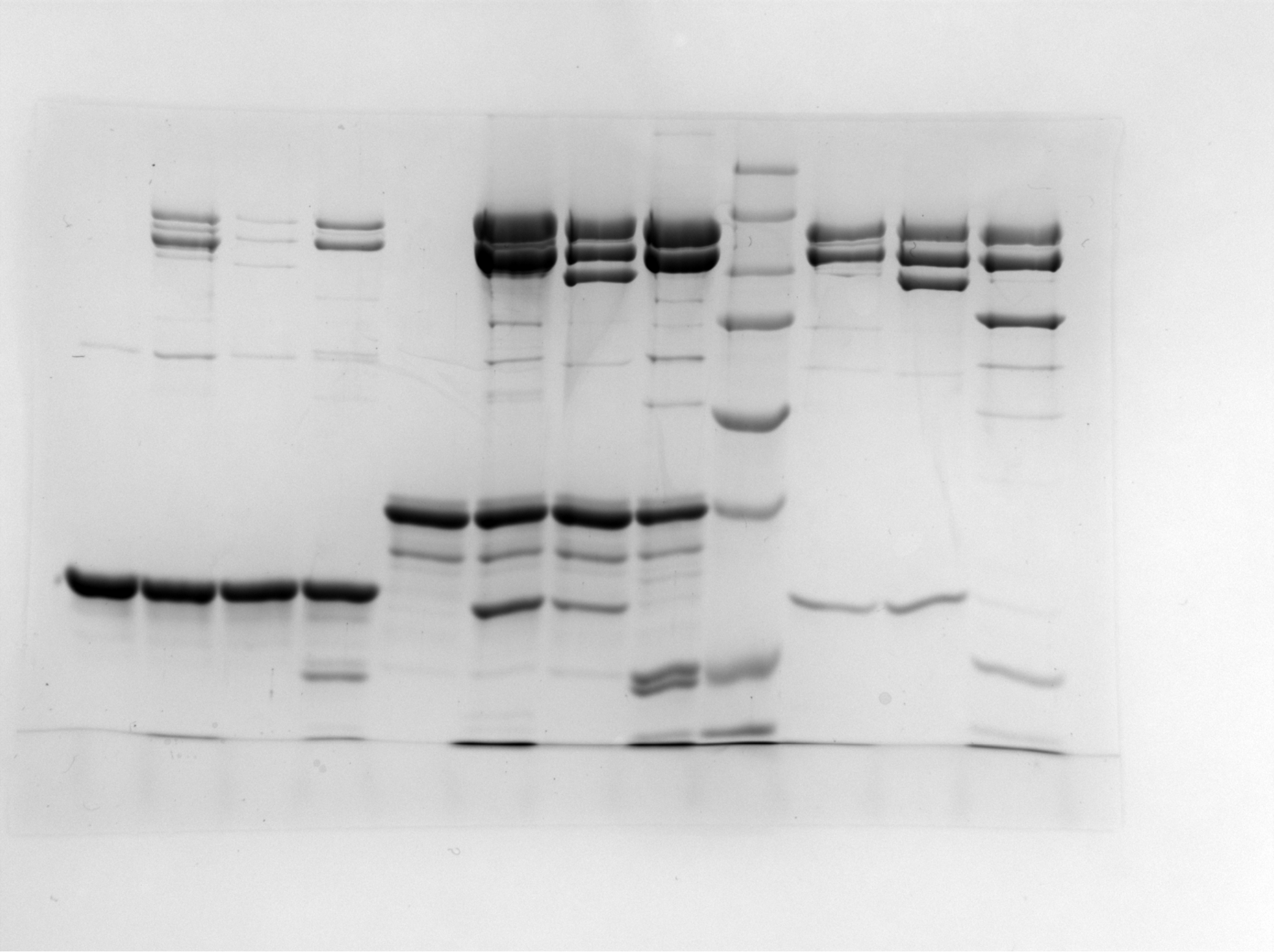

Supplement: Figure 3—figure supplement 1—source data 1. [file elife-88492-fig3-figsupp1-data1.zip › Figure 3 - figure supplement 1 - source data 1/Figure 3ΓÇöFigure supplement 1A raw image.jpg]

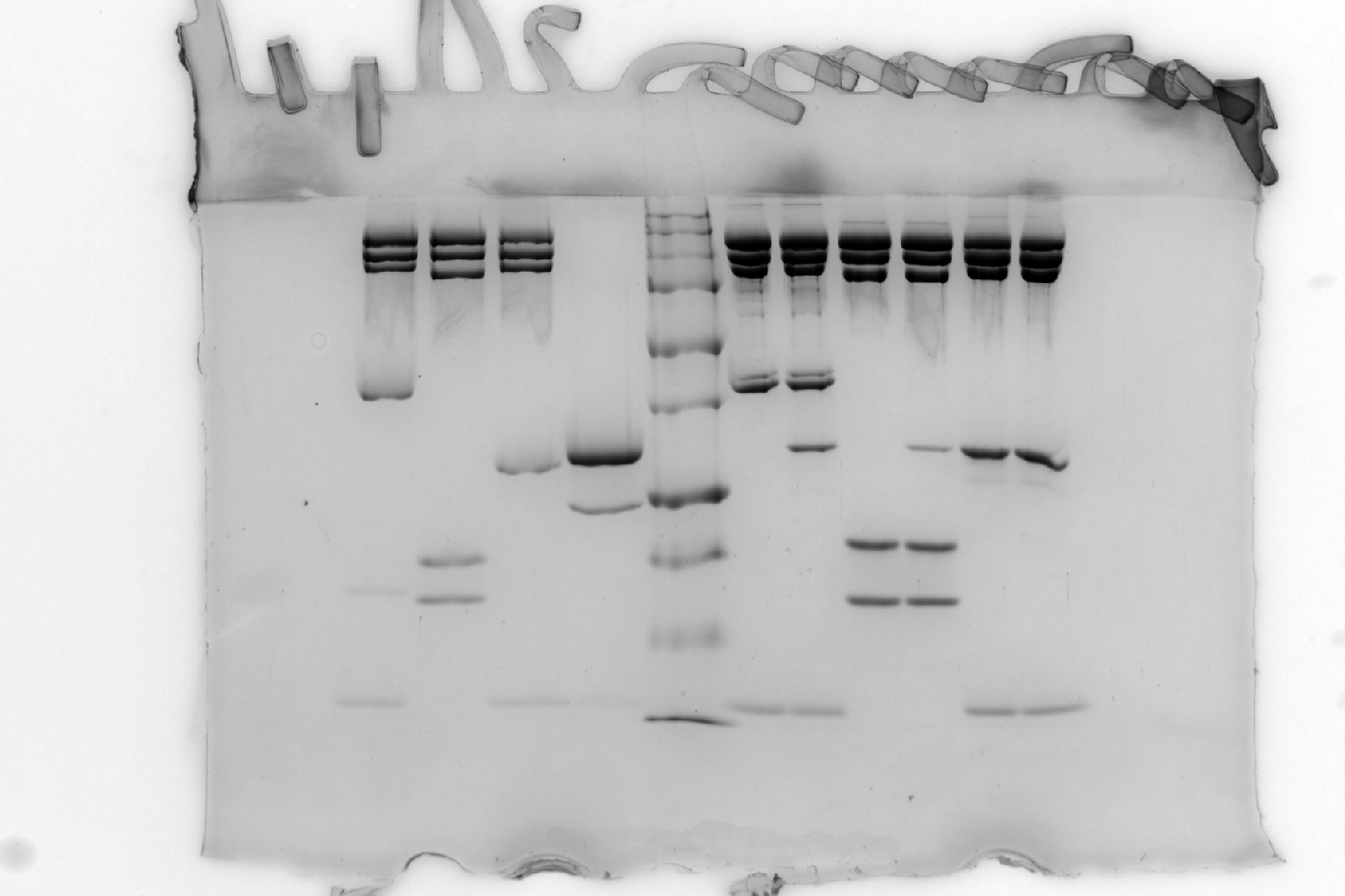

Supplement: Figure 3—figure supplement 1—source data 1. [file elife-88492-fig3-figsupp1-data1.zip › Figure 3 - figure supplement 1 - source data 1/Figure 3ΓÇöFigure supplement 1B raw image.jpg]

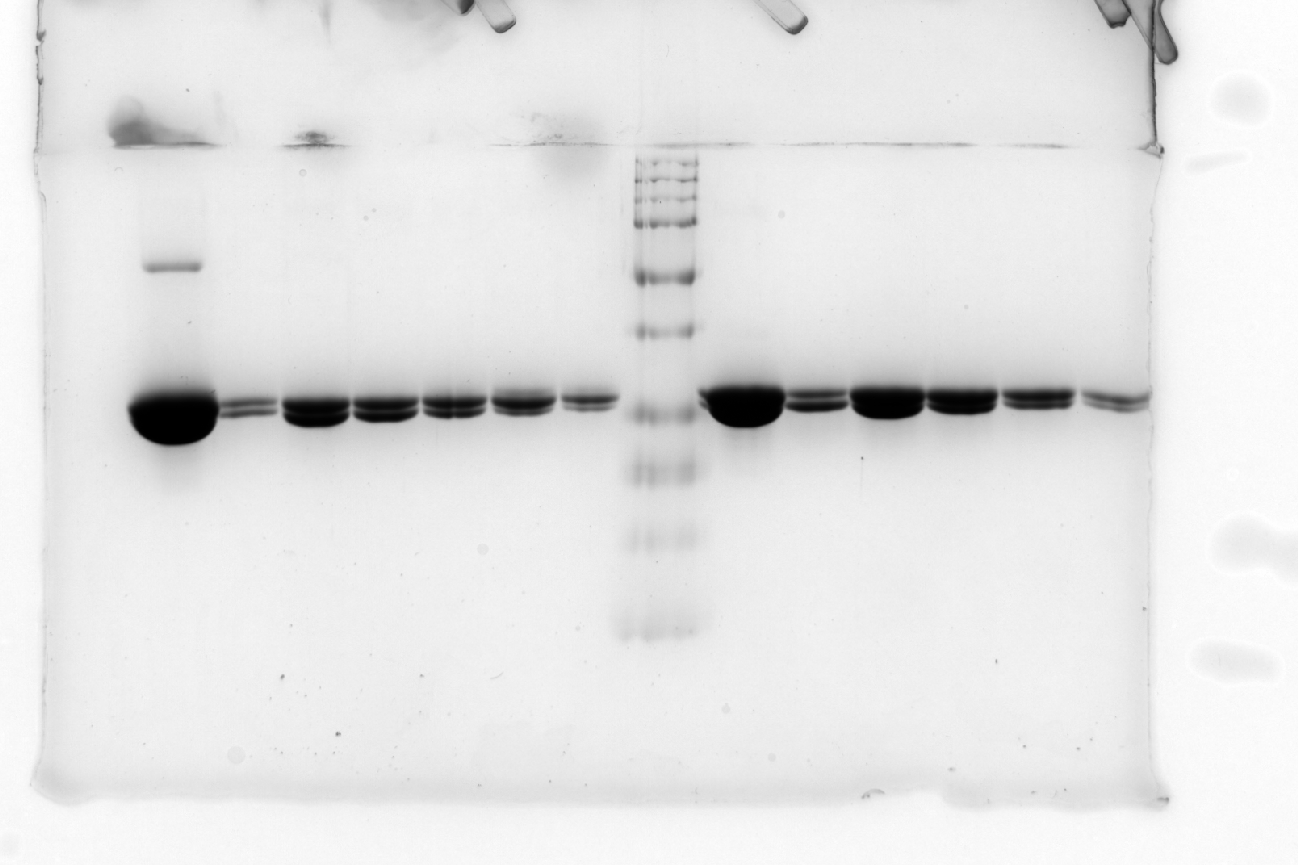

Supplement: Figure 3—figure supplement 1—source data 1. [file elife-88492-fig3-figsupp1-data1.zip › Figure 3 - figure supplement 1 - source data 1/Figure 3ΓÇöFigure supplement 1D raw image.jpg]

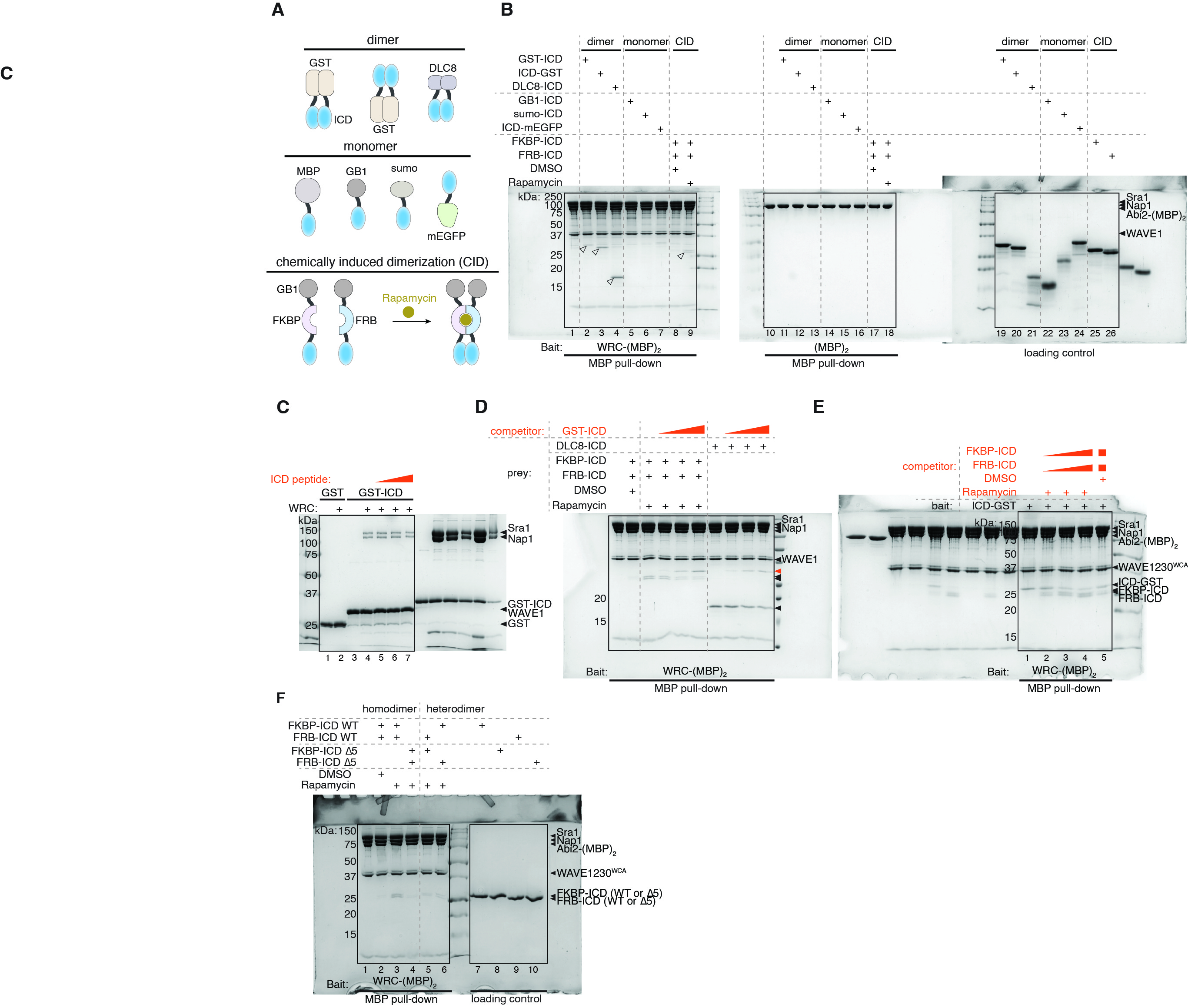

Supplement: Figure 3—figure supplement 2—source data 1. [file elife-88492-fig3-figsupp2-data1.zip › Figure 3 - figure supplement 2 - source data 1/Figure 3 - figure supplement 2 unedited.jpg]

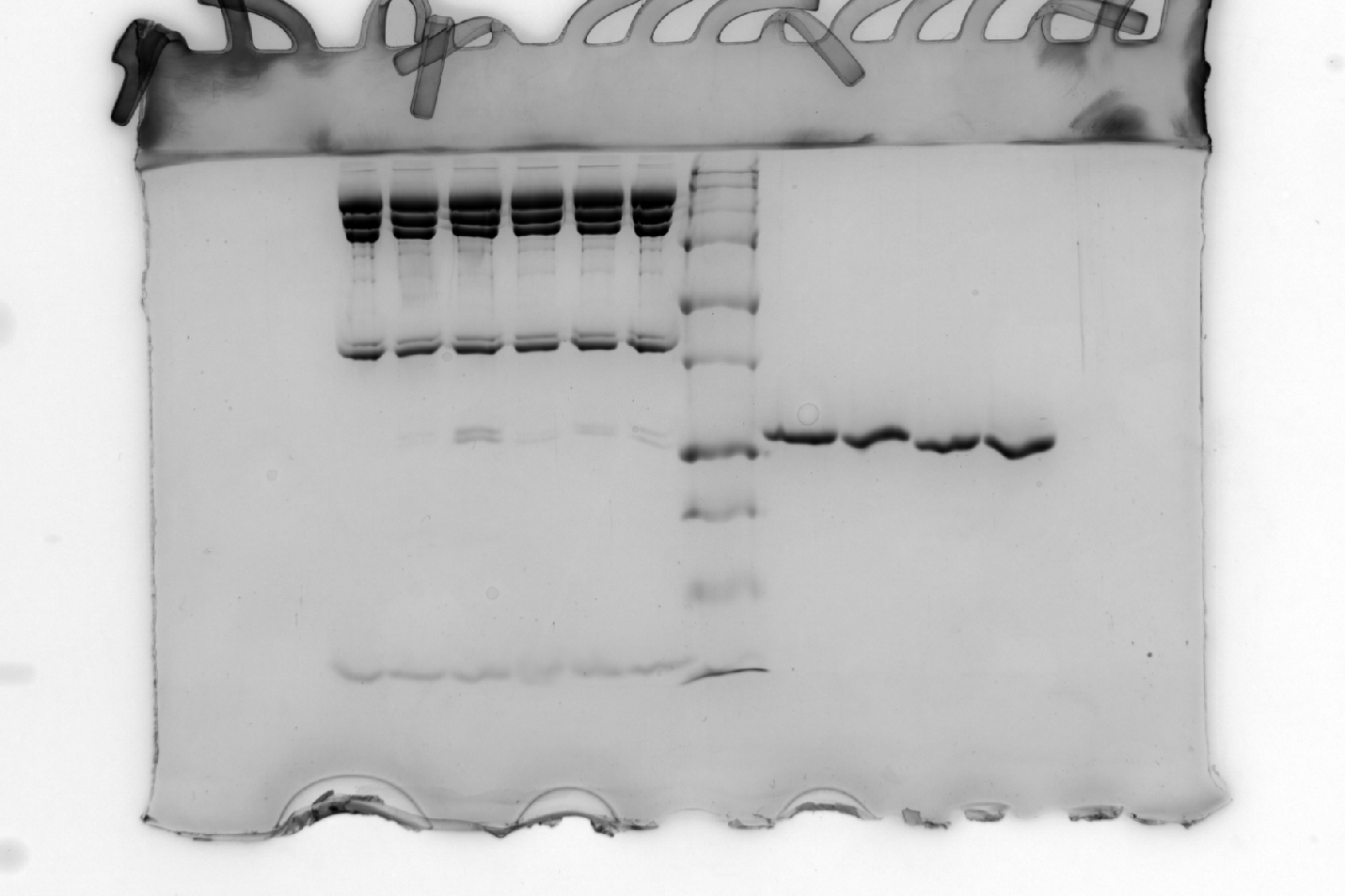

Supplement: Figure 3—figure supplement 2—source data 1. [file elife-88492-fig3-figsupp2-data1.zip › Figure 3 - figure supplement 2 - source data 1/Figure 3ΓÇöFigure supplement 2F raw image.jpg]

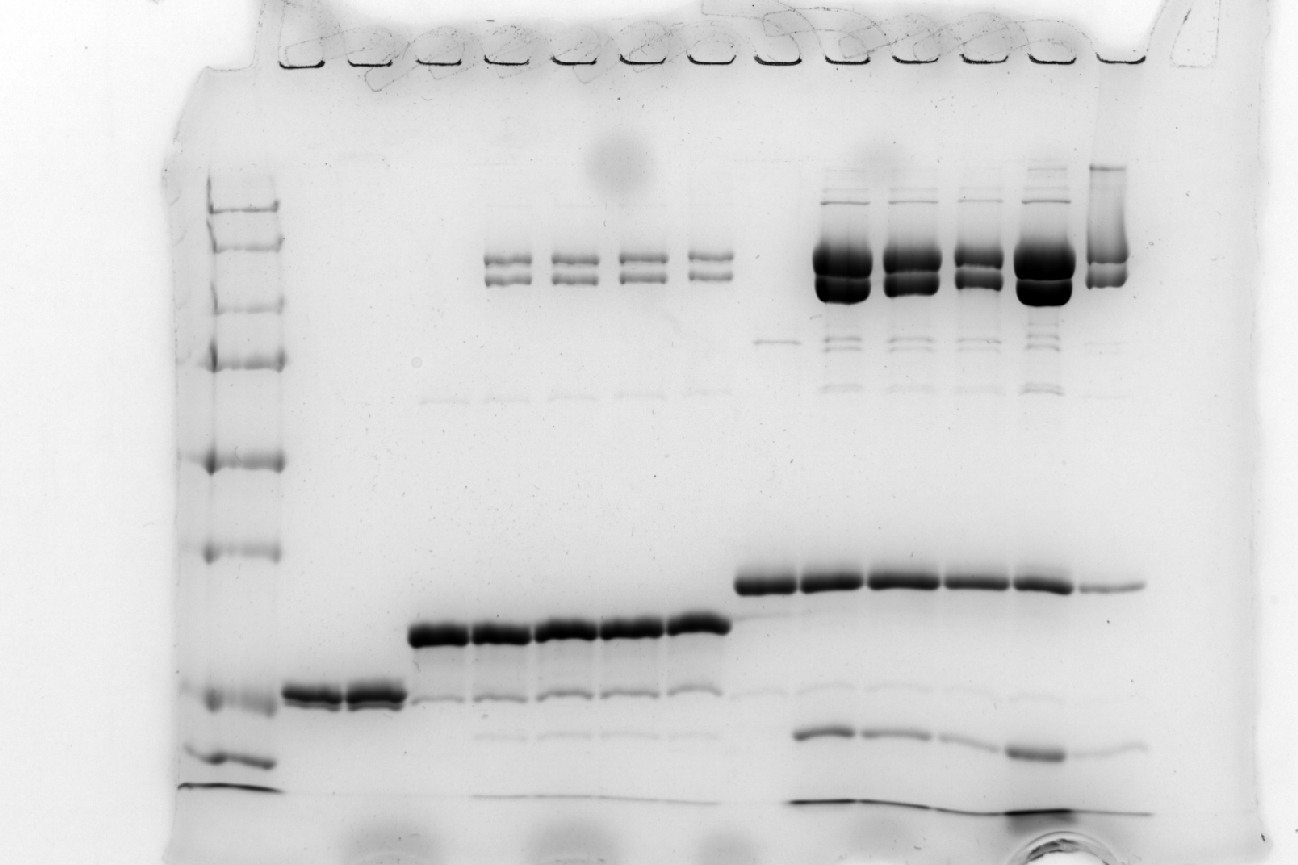

Supplement: Figure 3—figure supplement 2—source data 1. [file elife-88492-fig3-figsupp2-data1.zip › Figure 3 - figure supplement 2 - source data 1/Figure 3ΓÇöFigure supplement 2C raw image.jpg]

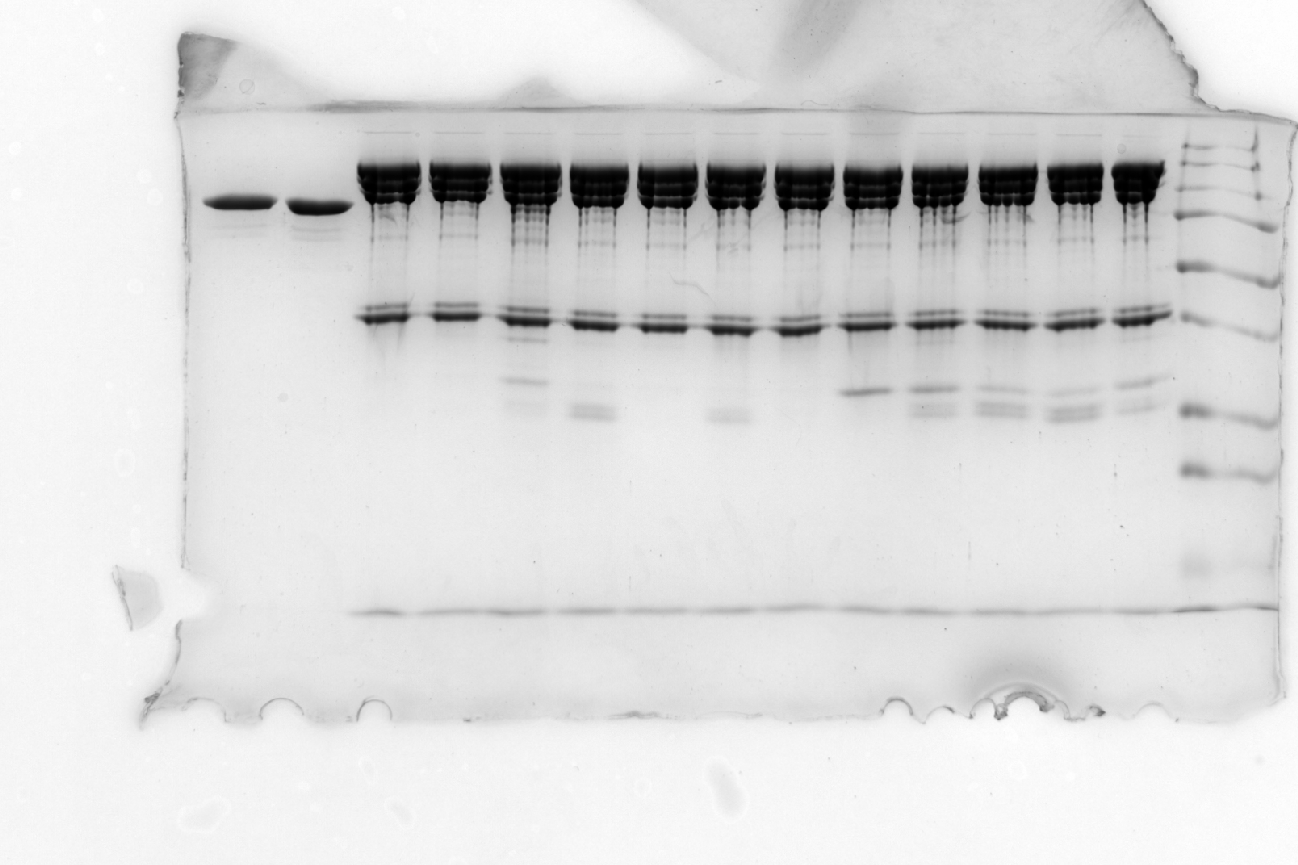

Supplement: Figure 3—figure supplement 2—source data 1. [file elife-88492-fig3-figsupp2-data1.zip › Figure 3 - figure supplement 2 - source data 1/Figure 3ΓÇöFigure supplement 2D and E raw image.jpg]

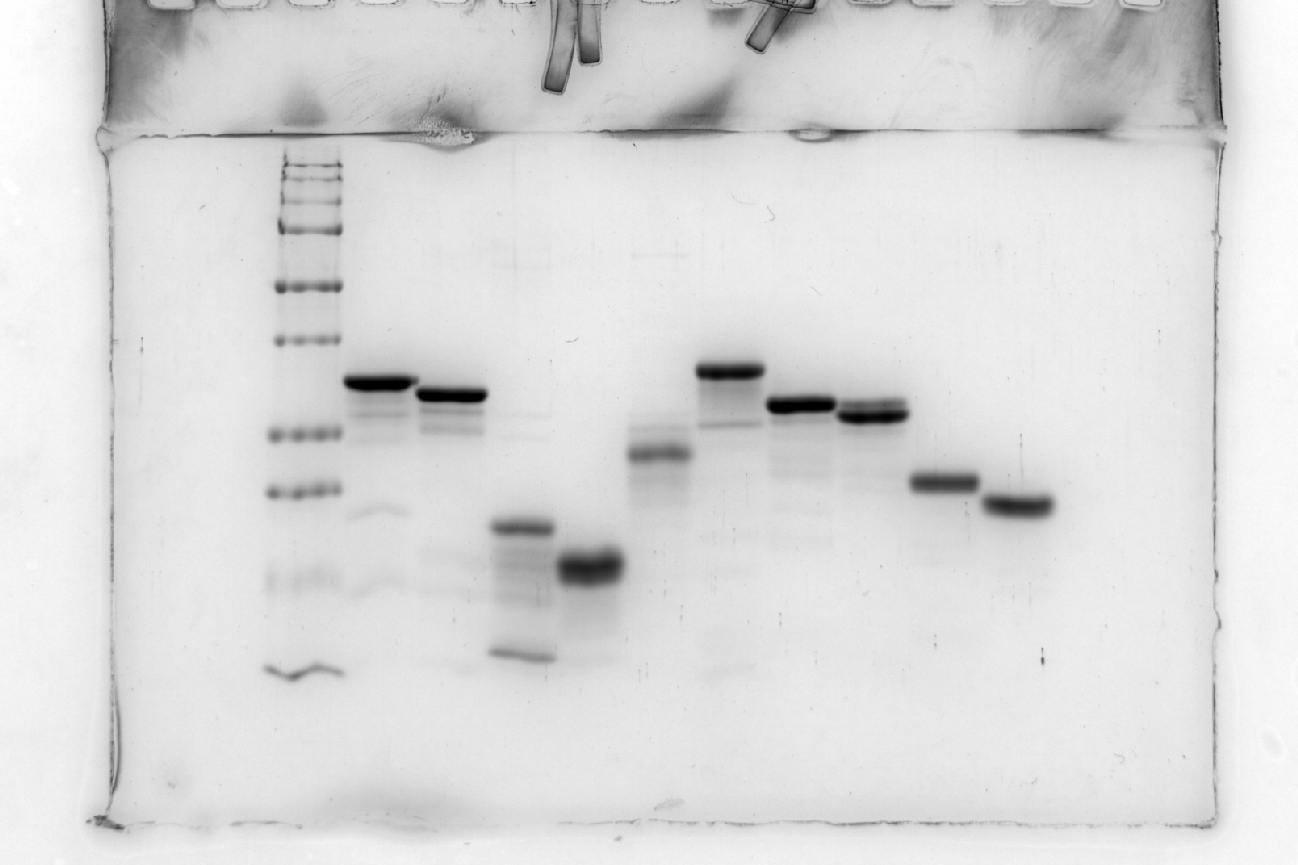

Supplement: Figure 3—figure supplement 2—source data 1. [file elife-88492-fig3-figsupp2-data1.zip › Figure 3 - figure supplement 2 - source data 1/Figure 3- figure supplement 2B Right raw image.jpg]

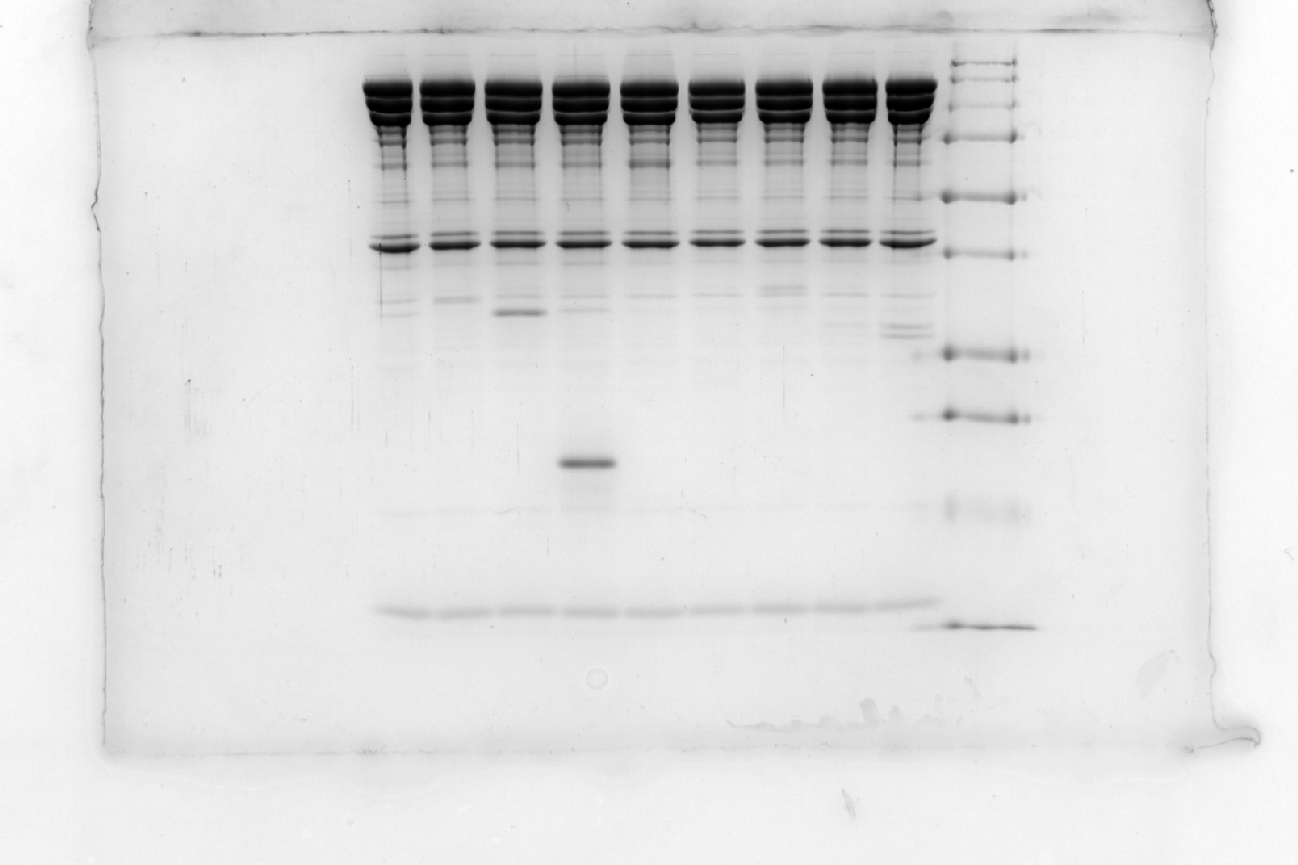

Supplement: Figure 3—figure supplement 2—source data 1. [file elife-88492-fig3-figsupp2-data1.zip › Figure 3 - figure supplement 2 - source data 1/Figure 3 - figure supplement 2B Left raw image.jpg]

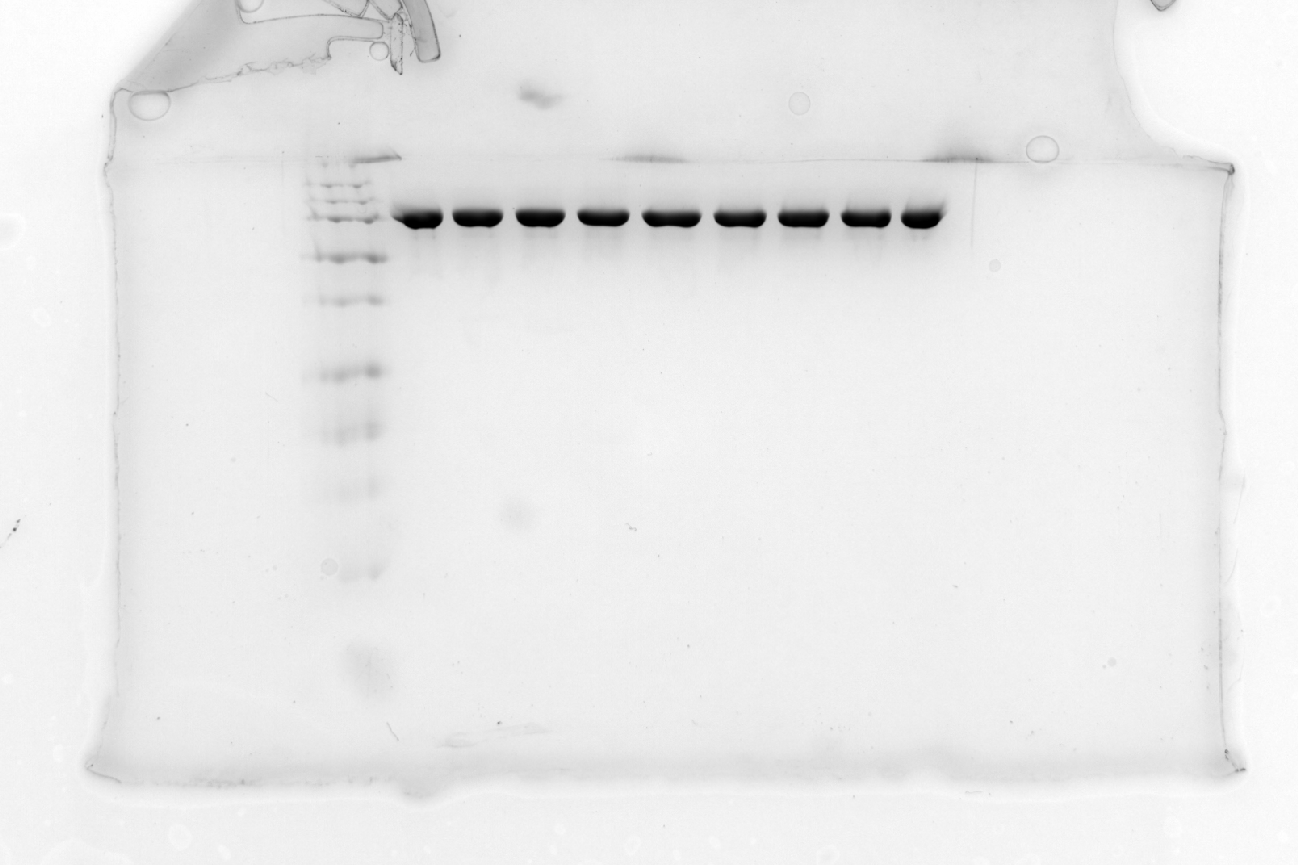

Supplement: Figure 3—figure supplement 2—source data 1. [file elife-88492-fig3-figsupp2-data1.zip › Figure 3 - figure supplement 2 - source data 1/Figure 3- figure supplement 2B Middle raw image.jpg]

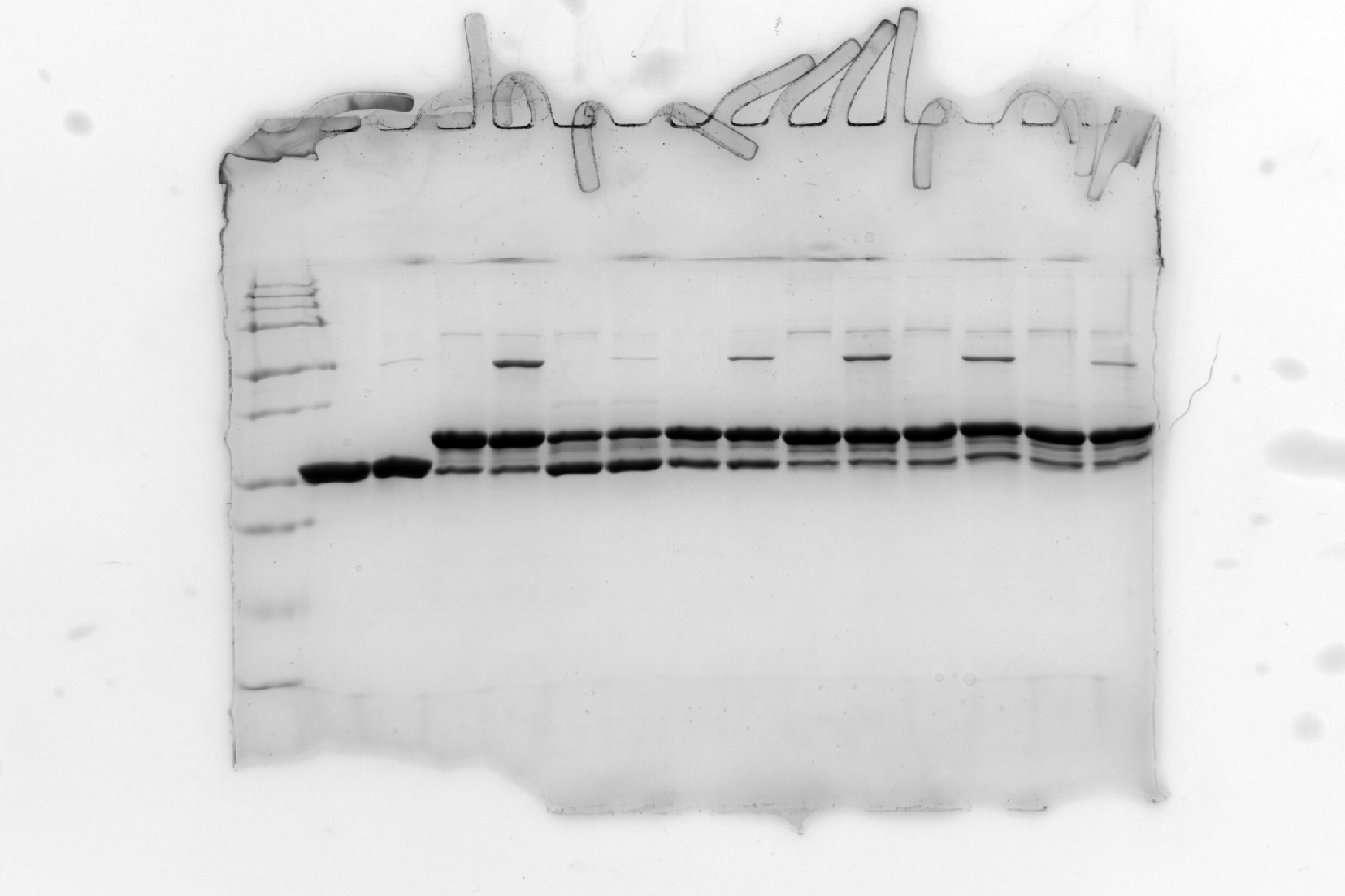

Supplement: Figure 4—source data 1. [file elife-88492-fig4-data1.zip › Figure 4 - source data 1/Figure 4B Middle Raw Gel Image.tif]

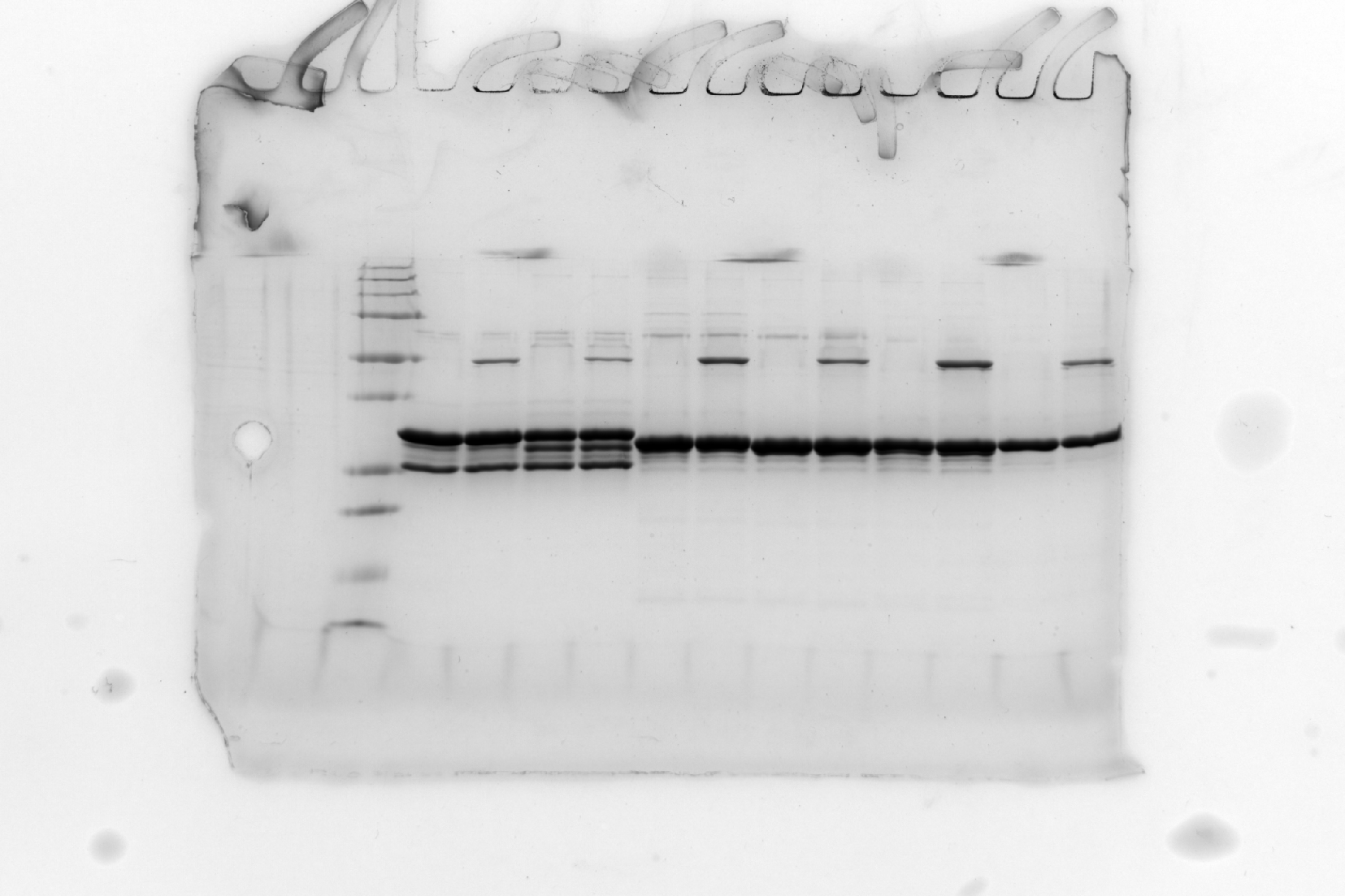

Supplement: Figure 4—source data 1. [file elife-88492-fig4-data1.zip › Figure 4 - source data 1/Figure 4B Right Raw Gel Image.tif]

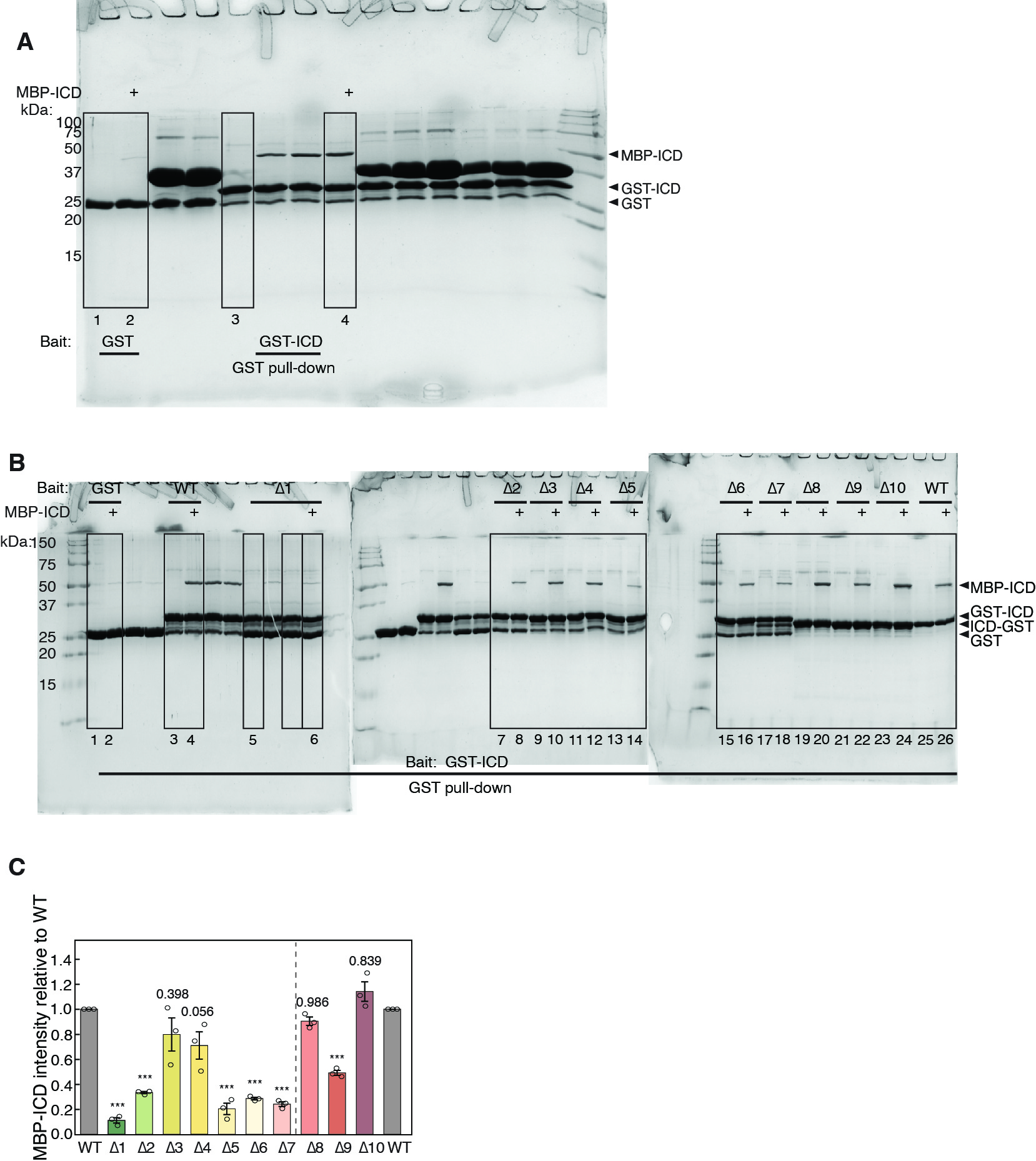

Supplement: Figure 4—source data 1. [file elife-88492-fig4-data1.zip › Figure 4 - source data 1/Figure 4 unedited.jpg]

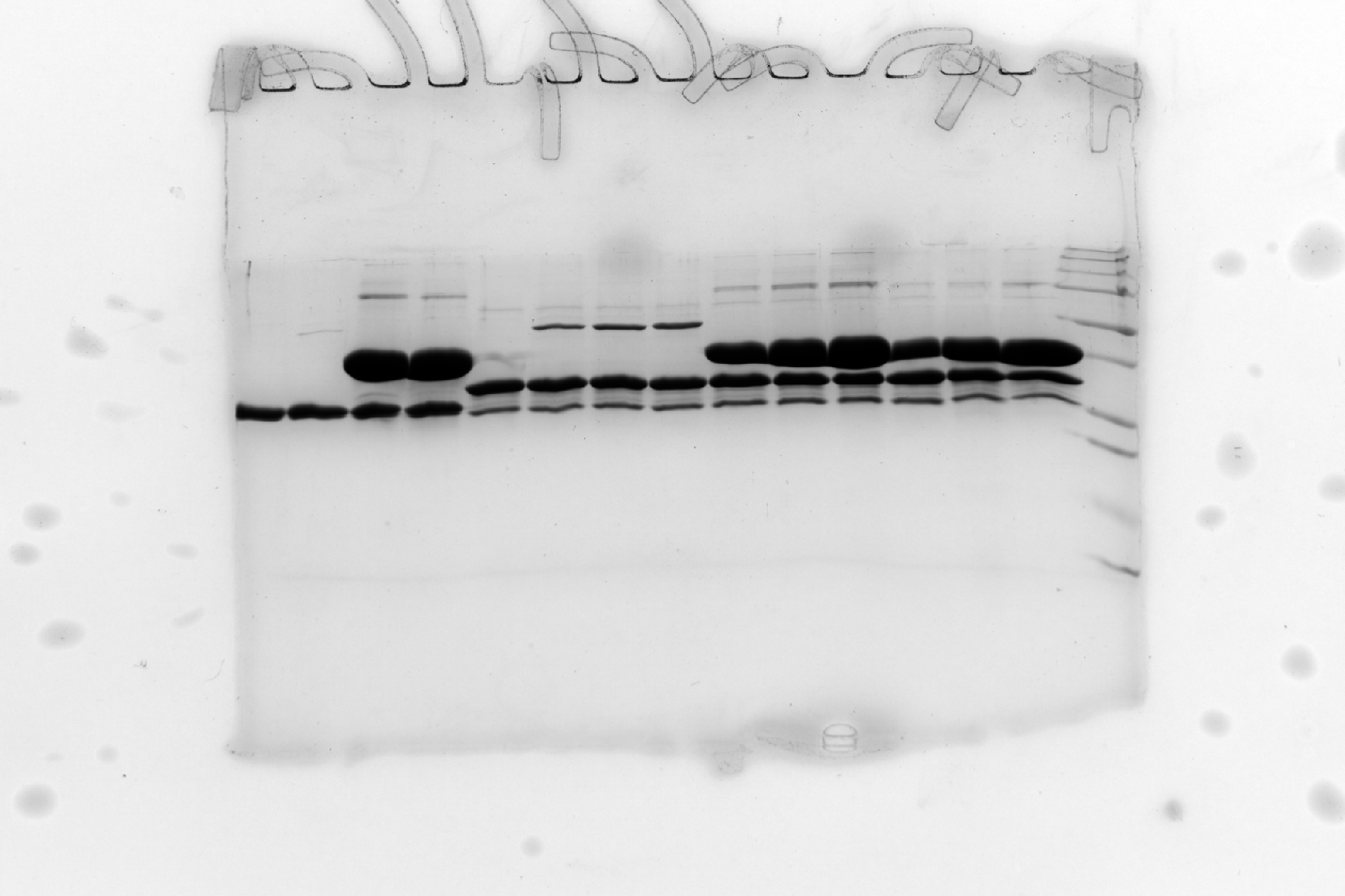

Supplement: Figure 4—source data 1. [file elife-88492-fig4-data1.zip › Figure 4 - source data 1/Figure 4A Raw Gel Image.tif]

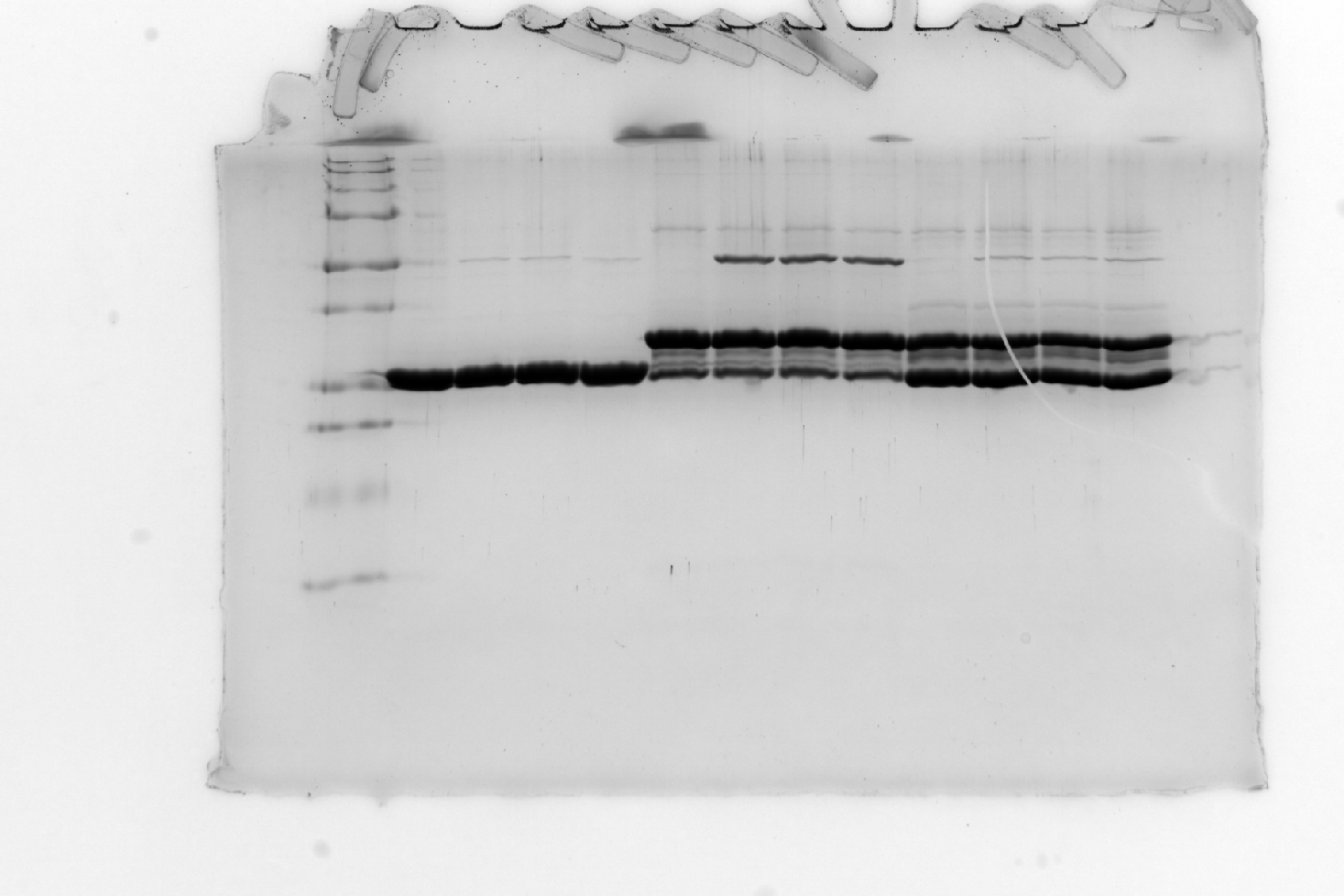

Supplement: Figure 4—source data 1. [file elife-88492-fig4-data1.zip › Figure 4 - source data 1/Figure 4B Left Raw Gel Image.tif]

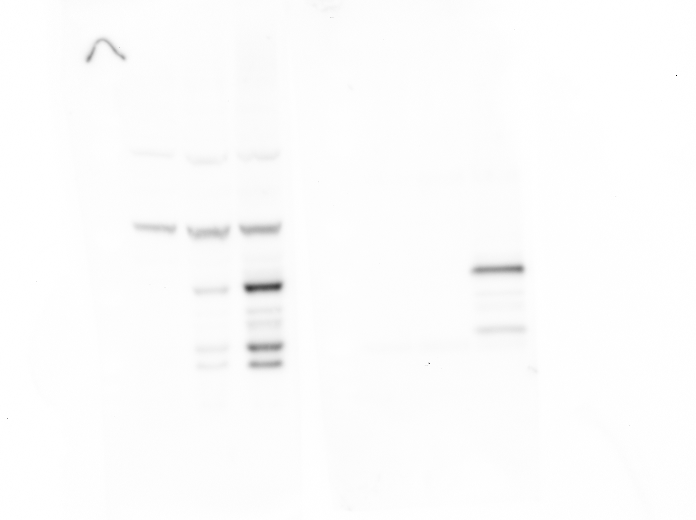

Supplement: Figure 4—figure supplement 1—source data 1. [file elife-88492-fig4-figsupp1-data1.zip › Figure 4 - figure supplement 1 - source data 1/Figure 4 - figure supplement 1C bottom raw image.tif]

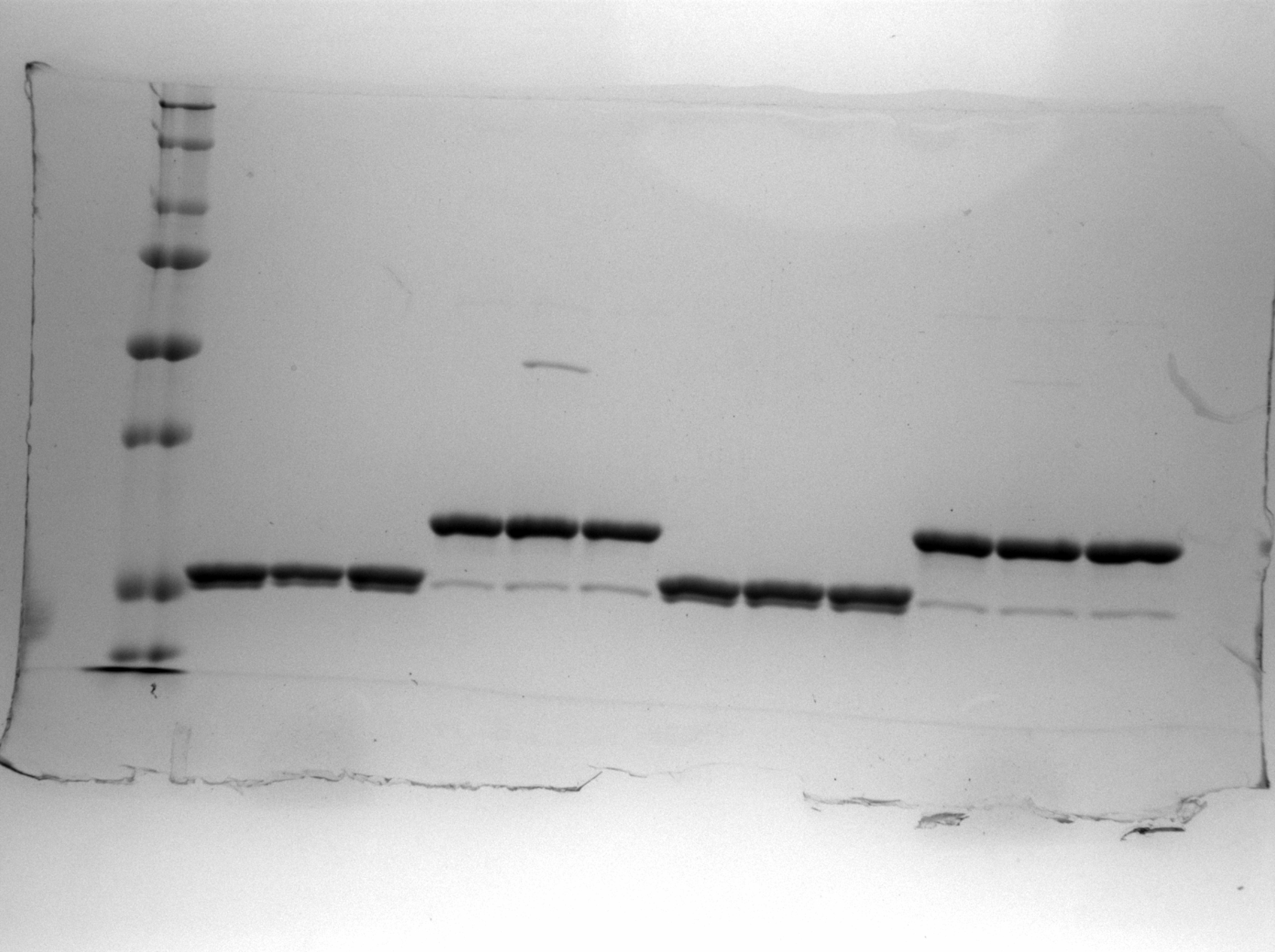

Supplement: Figure 4—figure supplement 1—source data 1. [file elife-88492-fig4-figsupp1-data1.zip › Figure 4 - figure supplement 1 - source data 1/Figure 4 - figure supplement 1A middle raw image.jpg]

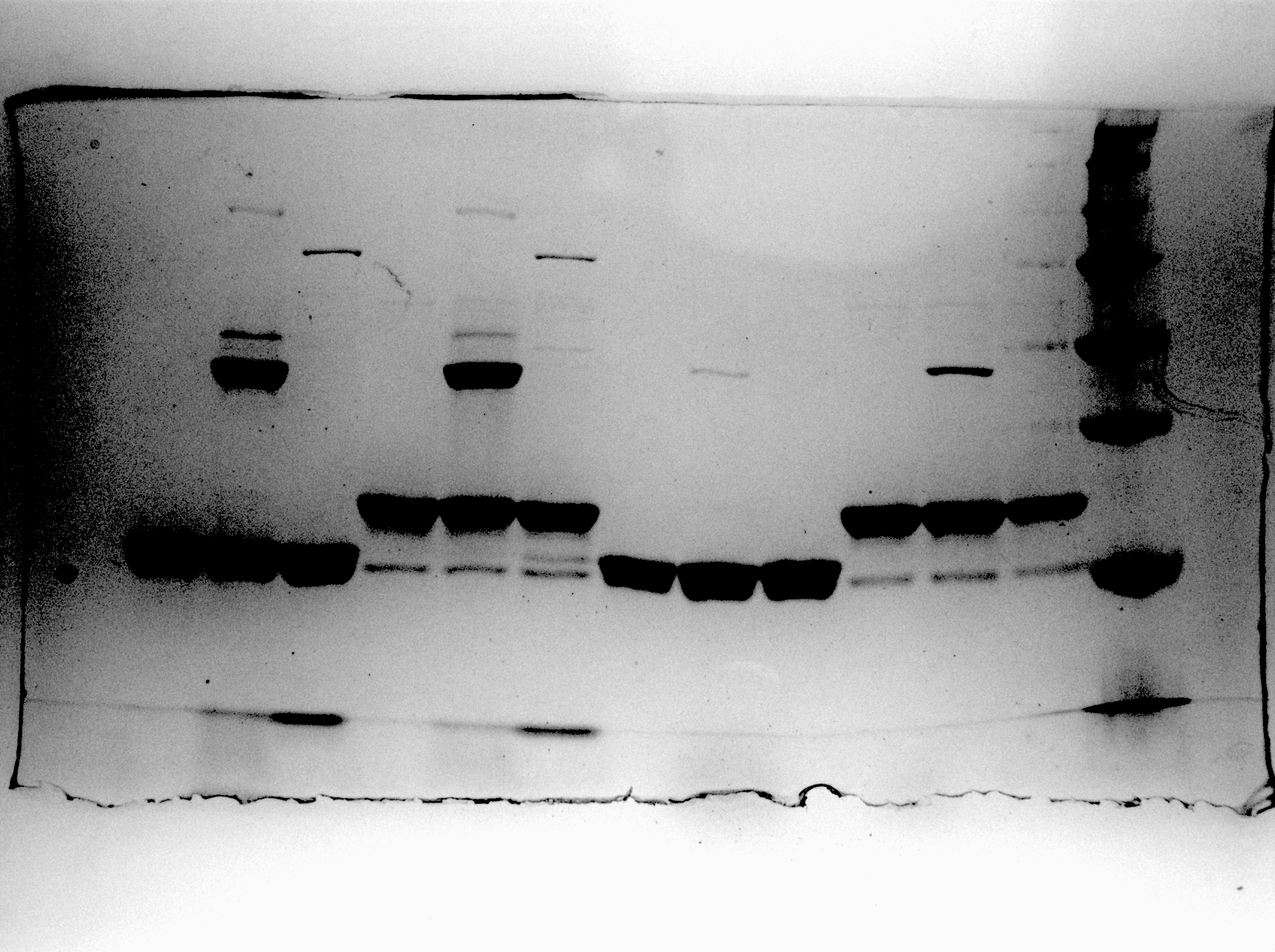

Supplement: Figure 4—figure supplement 1—source data 1. [file elife-88492-fig4-figsupp1-data1.zip › Figure 4 - figure supplement 1 - source data 1/Figure 4 - figure supplement 1B left raw image.tif]

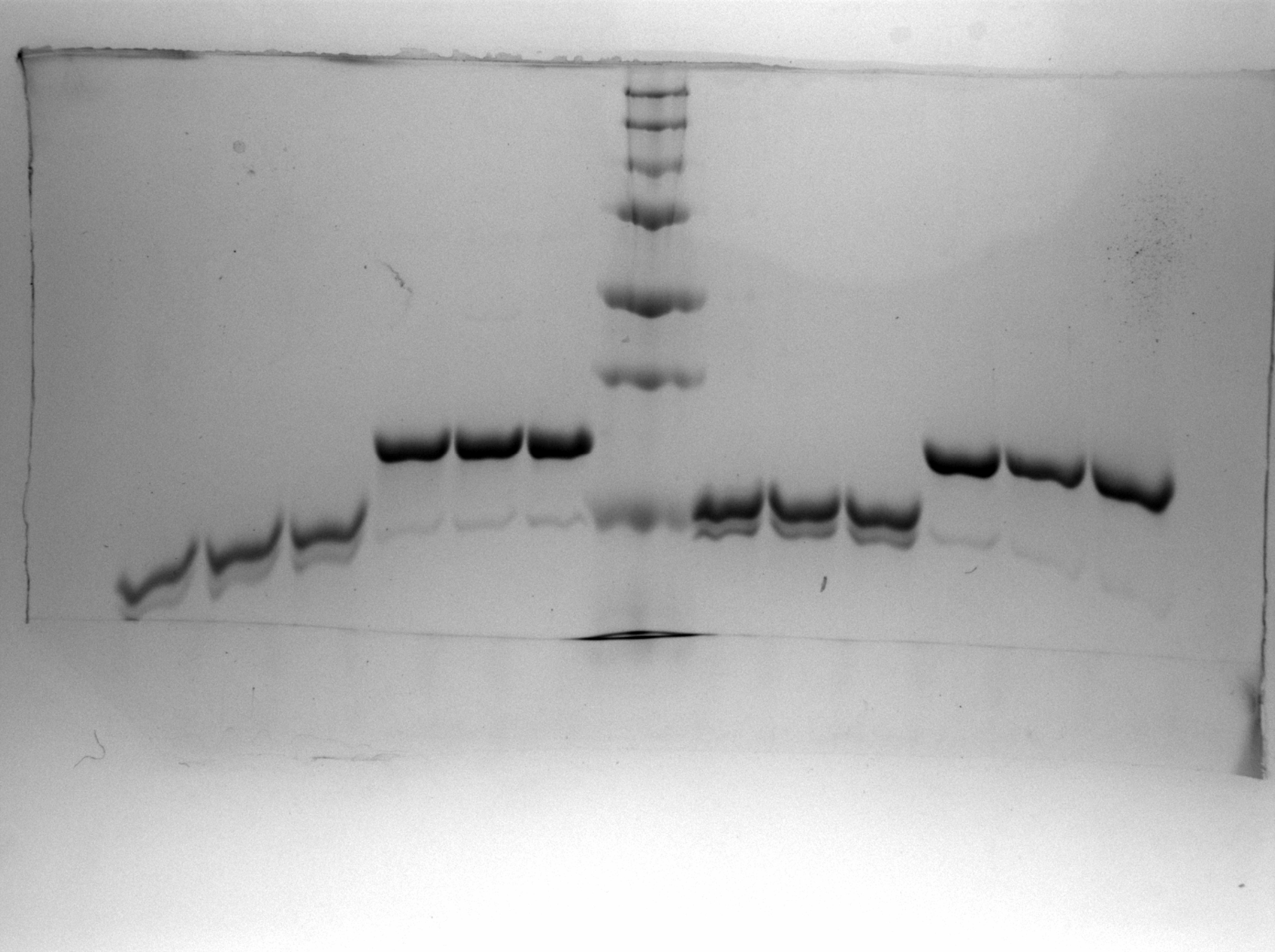

Supplement: Figure 4—figure supplement 1—source data 1. [file elife-88492-fig4-figsupp1-data1.zip › Figure 4 - figure supplement 1 - source data 1/Figure 4 - figure supplement 1A right raw image.jpg]

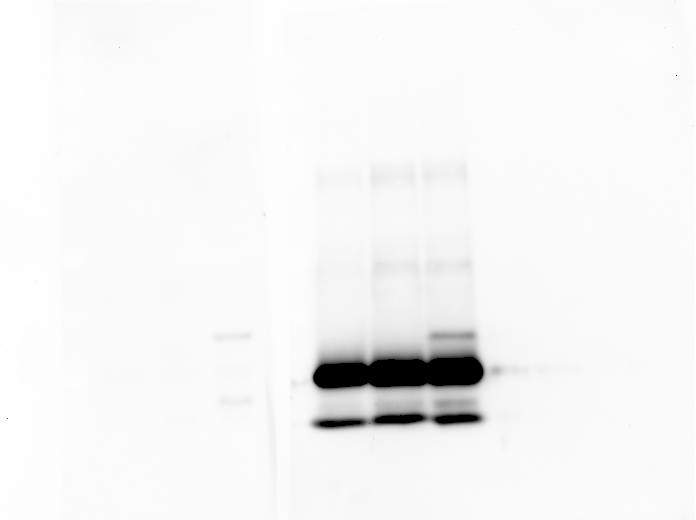

Supplement: Figure 4—figure supplement 1—source data 1. [file elife-88492-fig4-figsupp1-data1.zip › Figure 4 - figure supplement 1 - source data 1/Figure 4 - figure supplement 1C top raw image.tif]

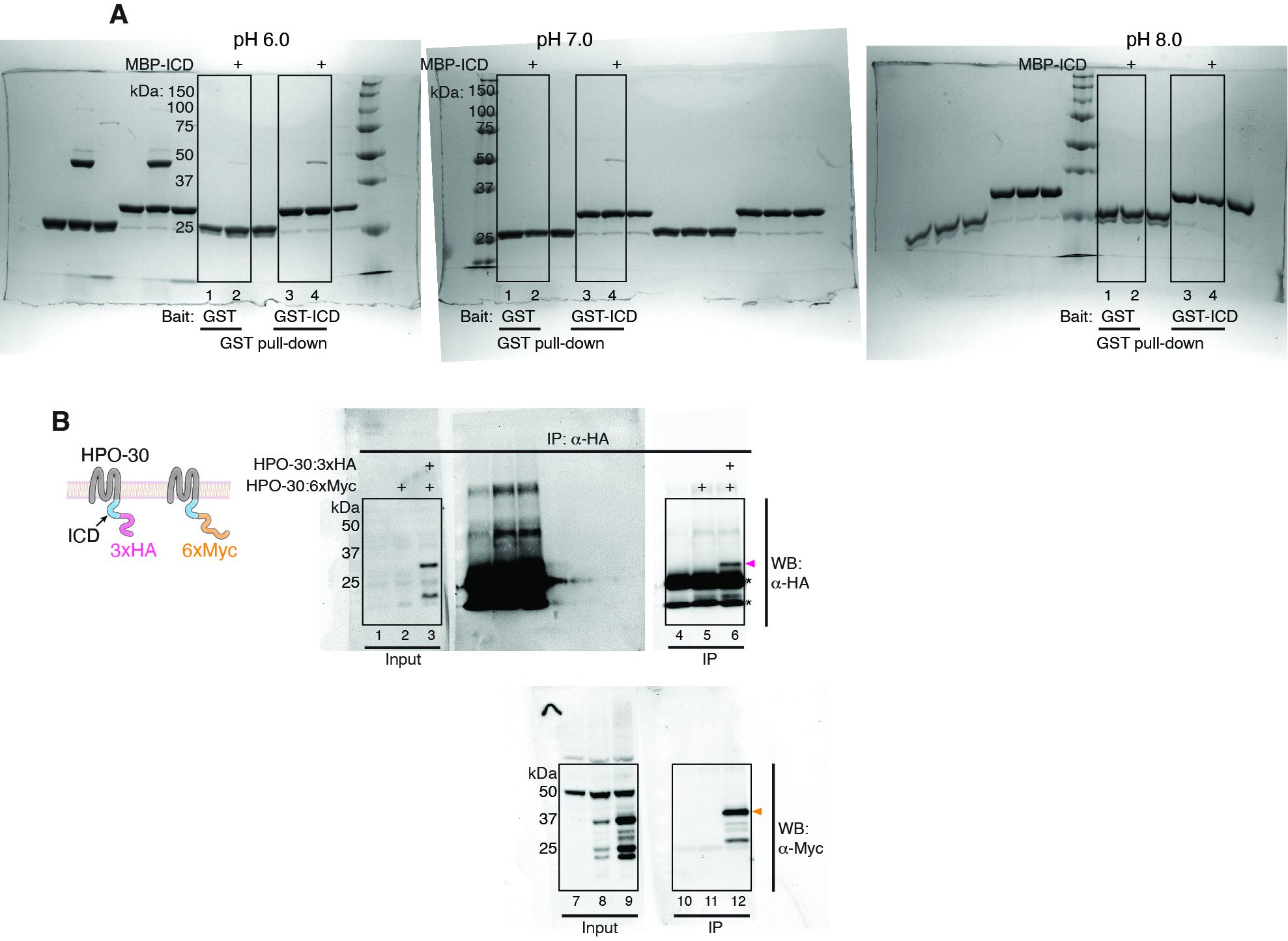

Supplement: Figure 4—figure supplement 1—source data 1. [file elife-88492-fig4-figsupp1-data1.zip › Figure 4 - figure supplement 1 - source data 1/Figure 4 - figure supplement 1 unedited.jpg]

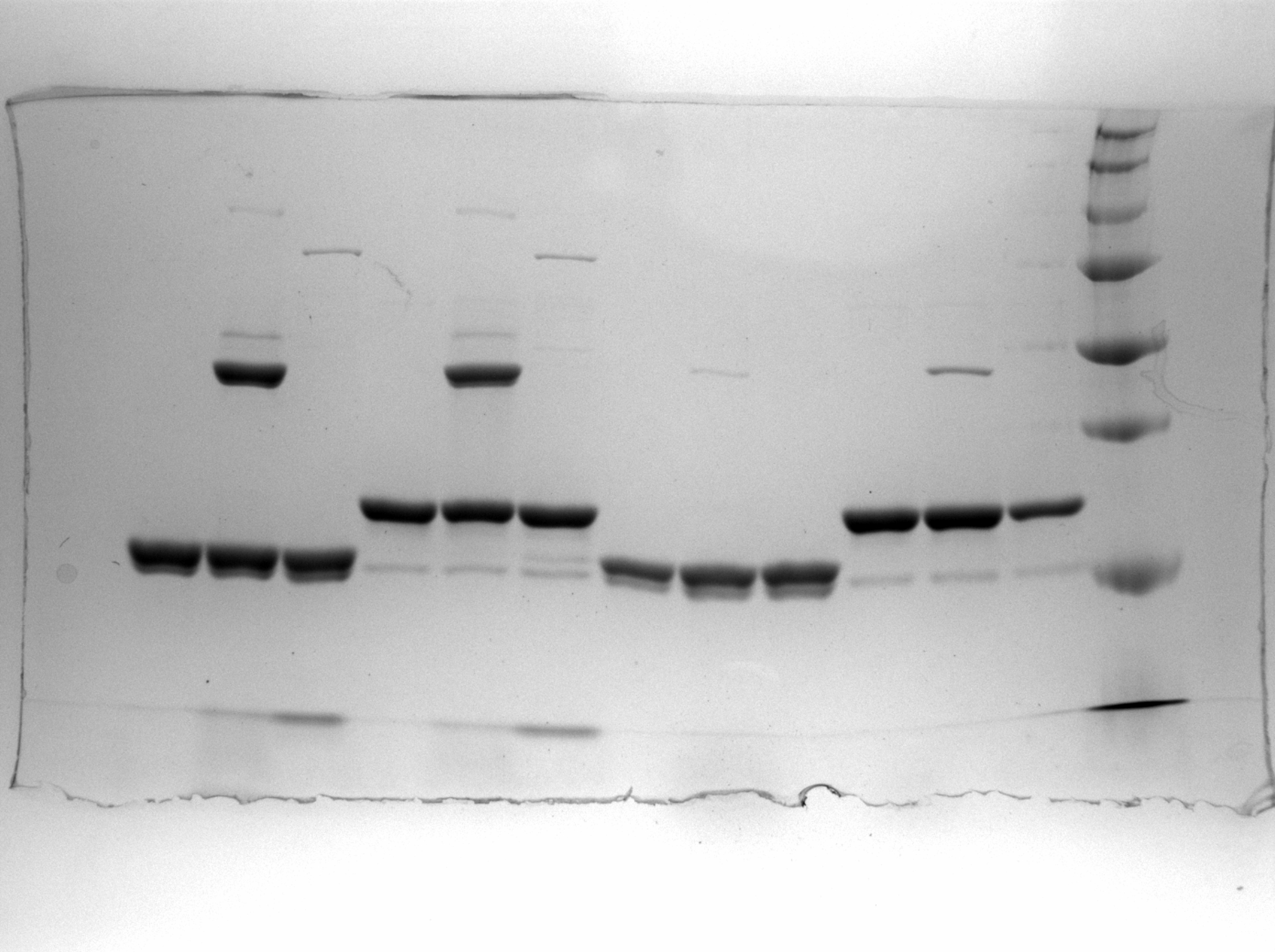

Supplement: Figure 4—figure supplement 1—source data 1. [file elife-88492-fig4-figsupp1-data1.zip › Figure 4 - figure supplement 1 - source data 1/Figure 4 - figure supplement 1A left raw image.jpg]

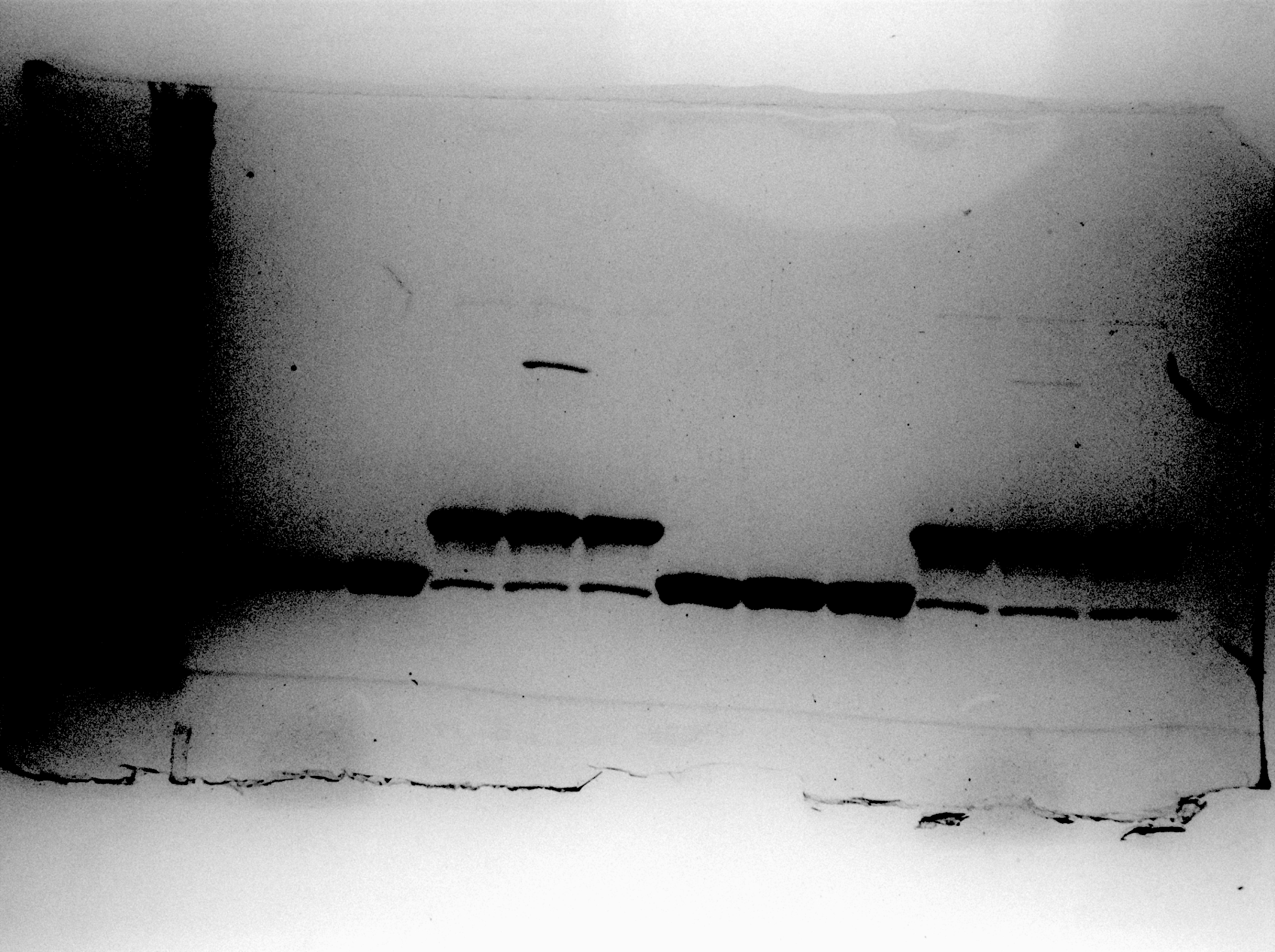

Supplement: Figure 4—figure supplement 1—source data 1. [file elife-88492-fig4-figsupp1-data1.zip › Figure 4 - figure supplement 1 - source data 1/Figure 4 - figure supplement 1B middle raw image.tif]

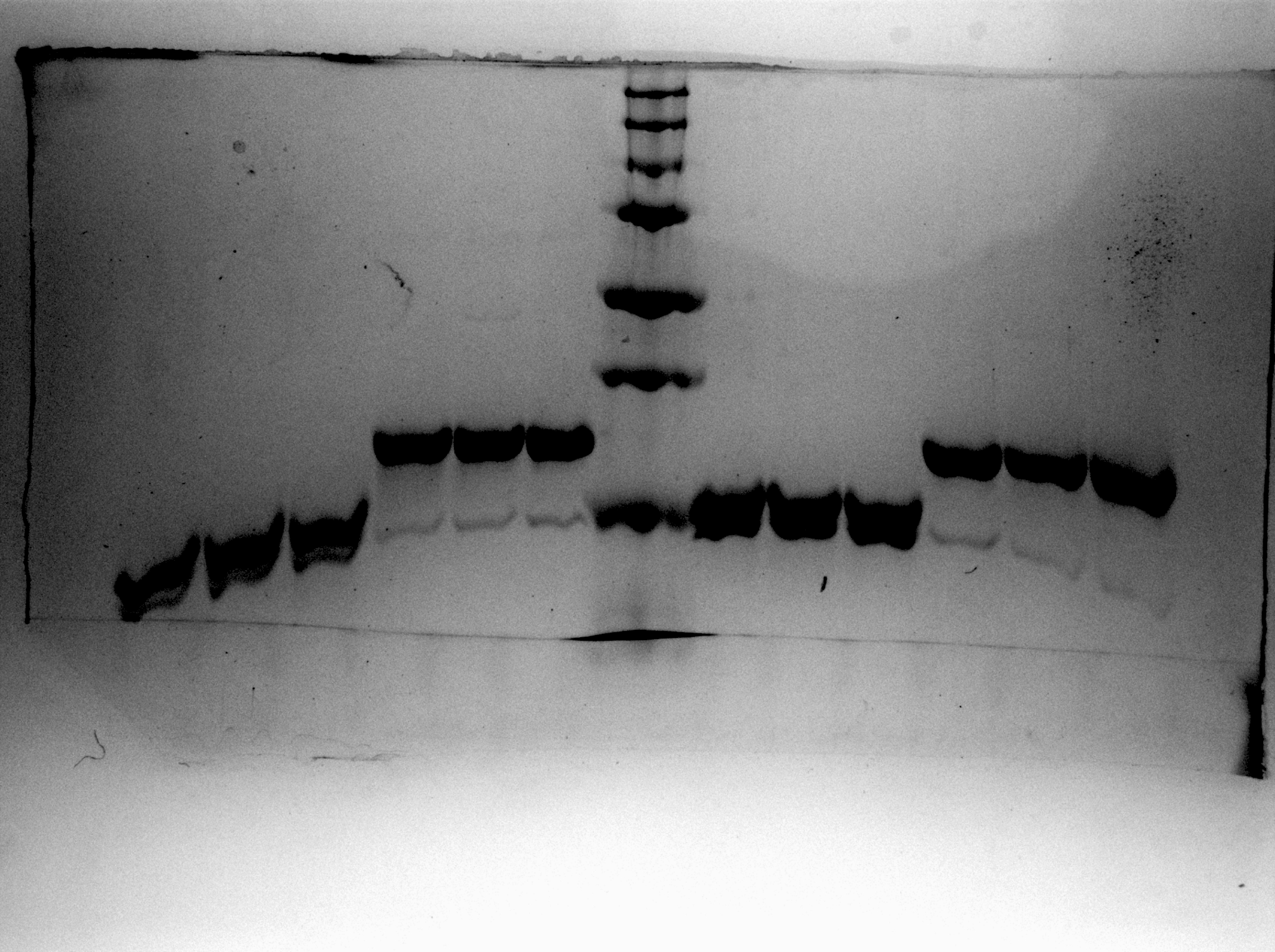

Supplement: Figure 4—figure supplement 1—source data 1. [file elife-88492-fig4-figsupp1-data1.zip › Figure 4 - figure supplement 1 - source data 1/Figure 4 - figure supplement 1B right raw image.tif]

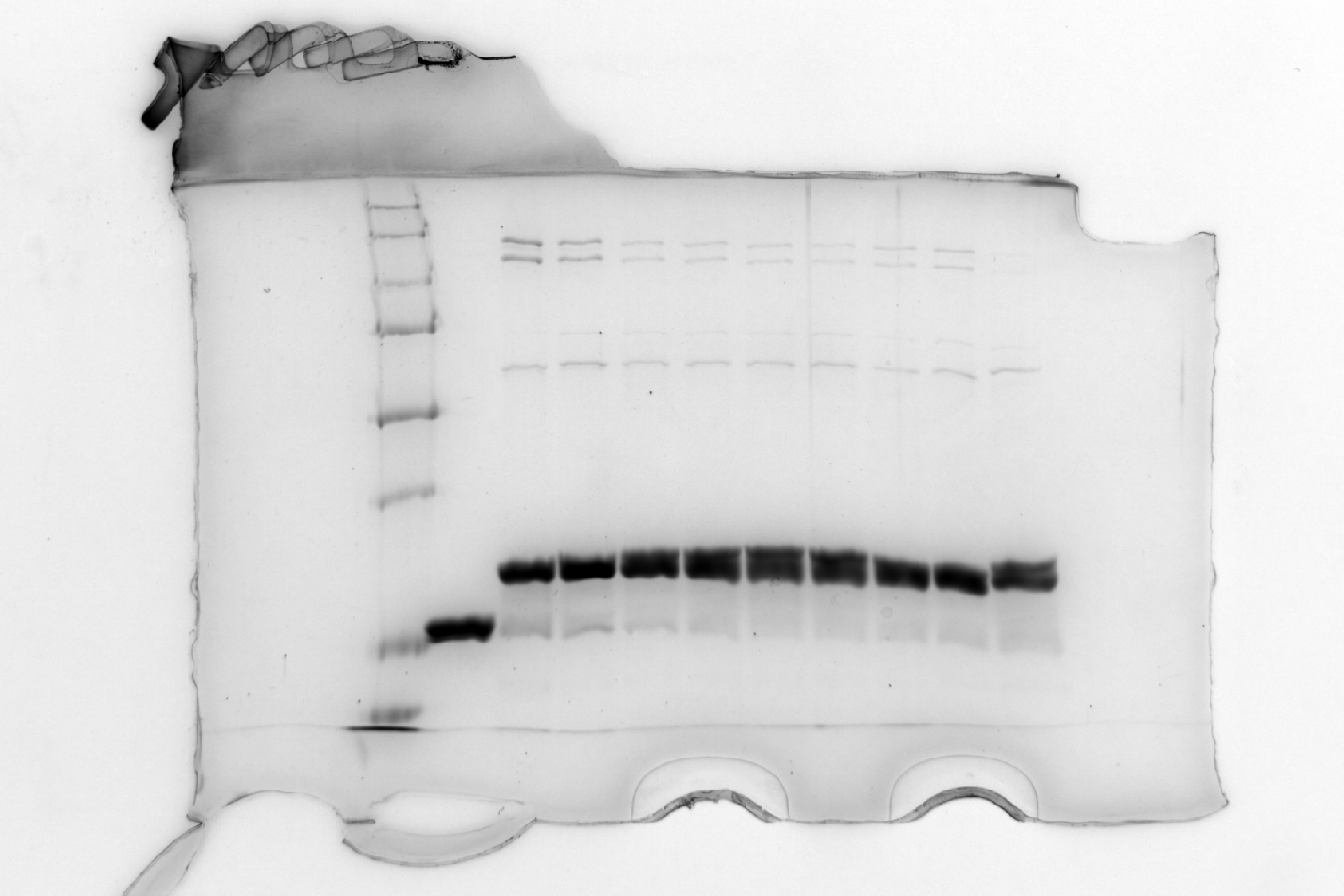

Supplement: Figure 4—figure supplement 2—source data 1. [file elife-88492-fig4-figsupp2-data1.zip › Figure 4 - figure supplement 2 - source data 1/Figure 4 - figure supplement 2C right raw image.tif]

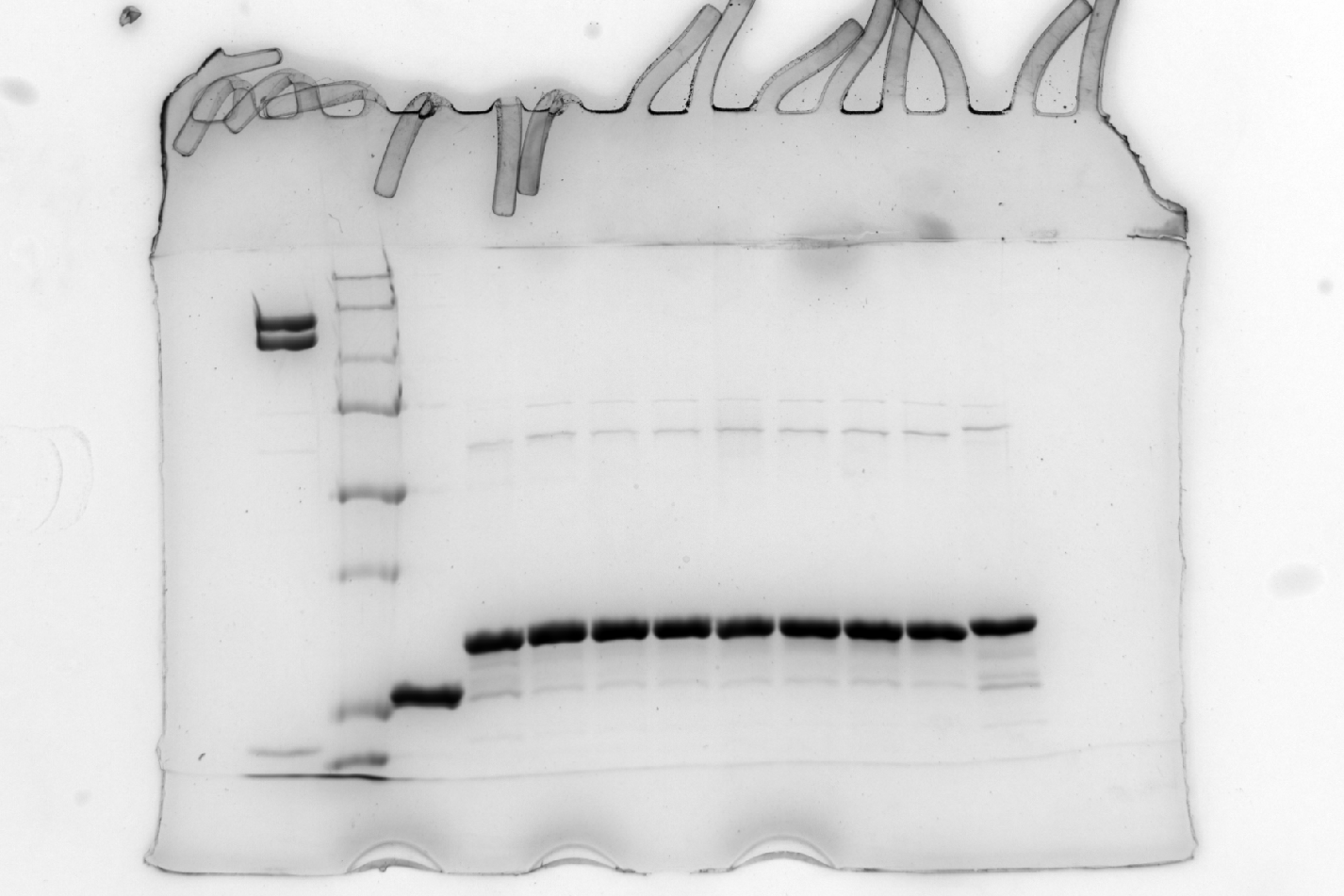

Supplement: Figure 4—figure supplement 2—source data 1. [file elife-88492-fig4-figsupp2-data1.zip › Figure 4 - figure supplement 2 - source data 1/Figure 4 - figure supplement 2C left raw image.tif]

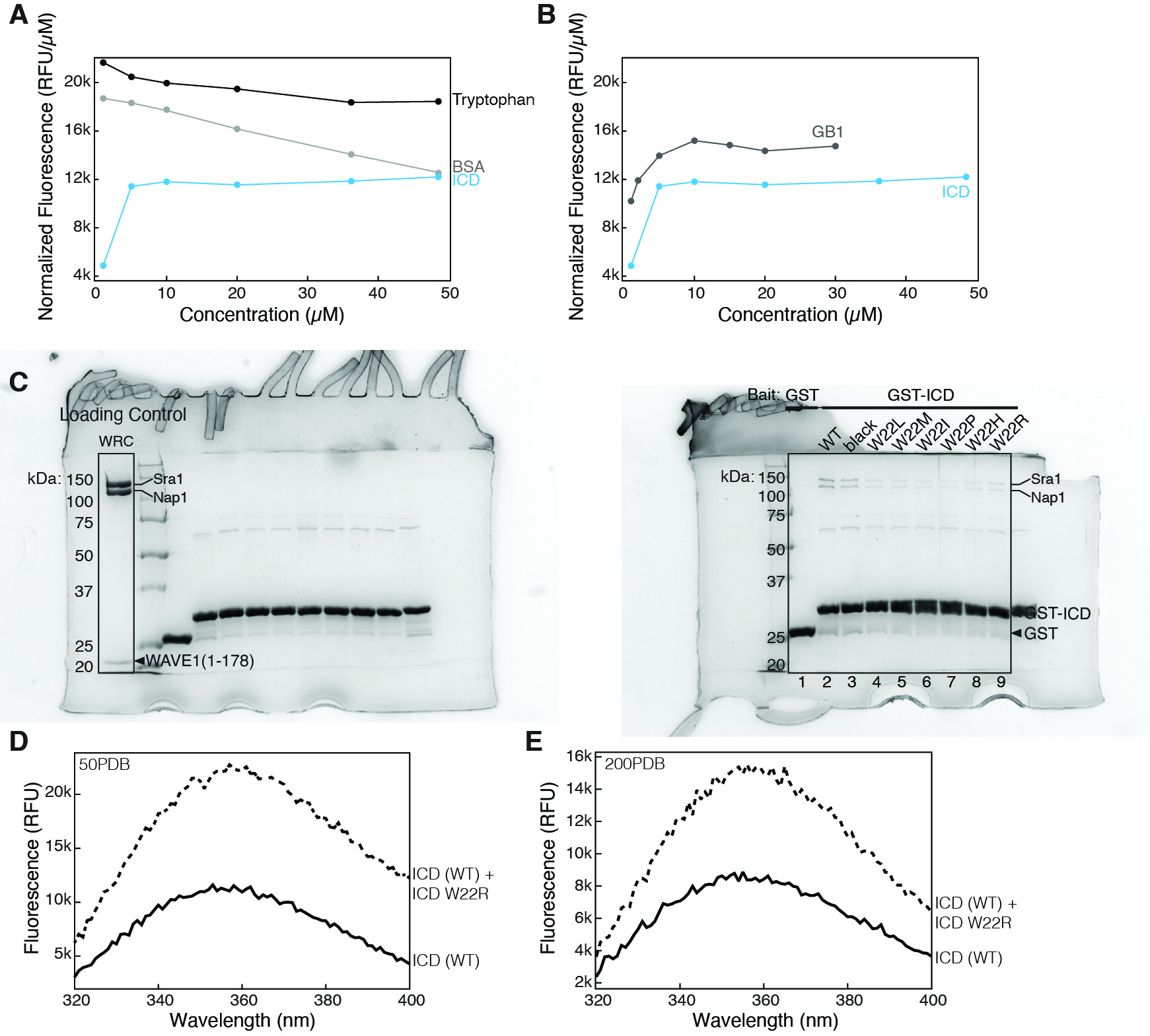

Supplement: Figure 4—figure supplement 2—source data 1. [file elife-88492-fig4-figsupp2-data1.zip › Figure 4 - figure supplement 2 - source data 1/Figure 4 - figure supplement 2 unedited.jpg]

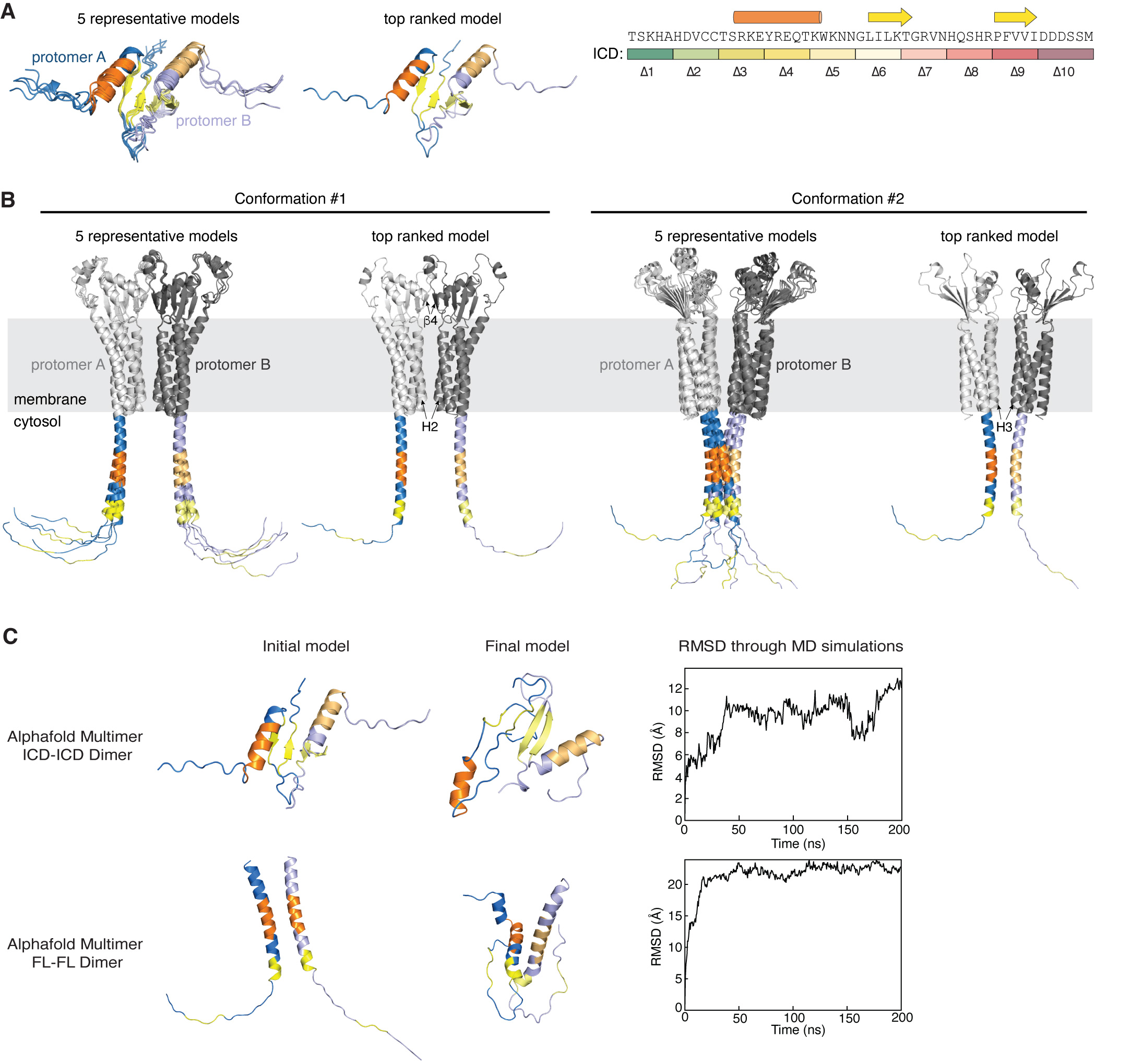

Supplement: Figure 4—figure supplement 3—source data 1. [file elife-88492-fig4-figsupp3-data1.zip › Figure 4 - figure supplement 3 - source data 1/Figure 4 - figure supplement 3 - unedited.jpg]

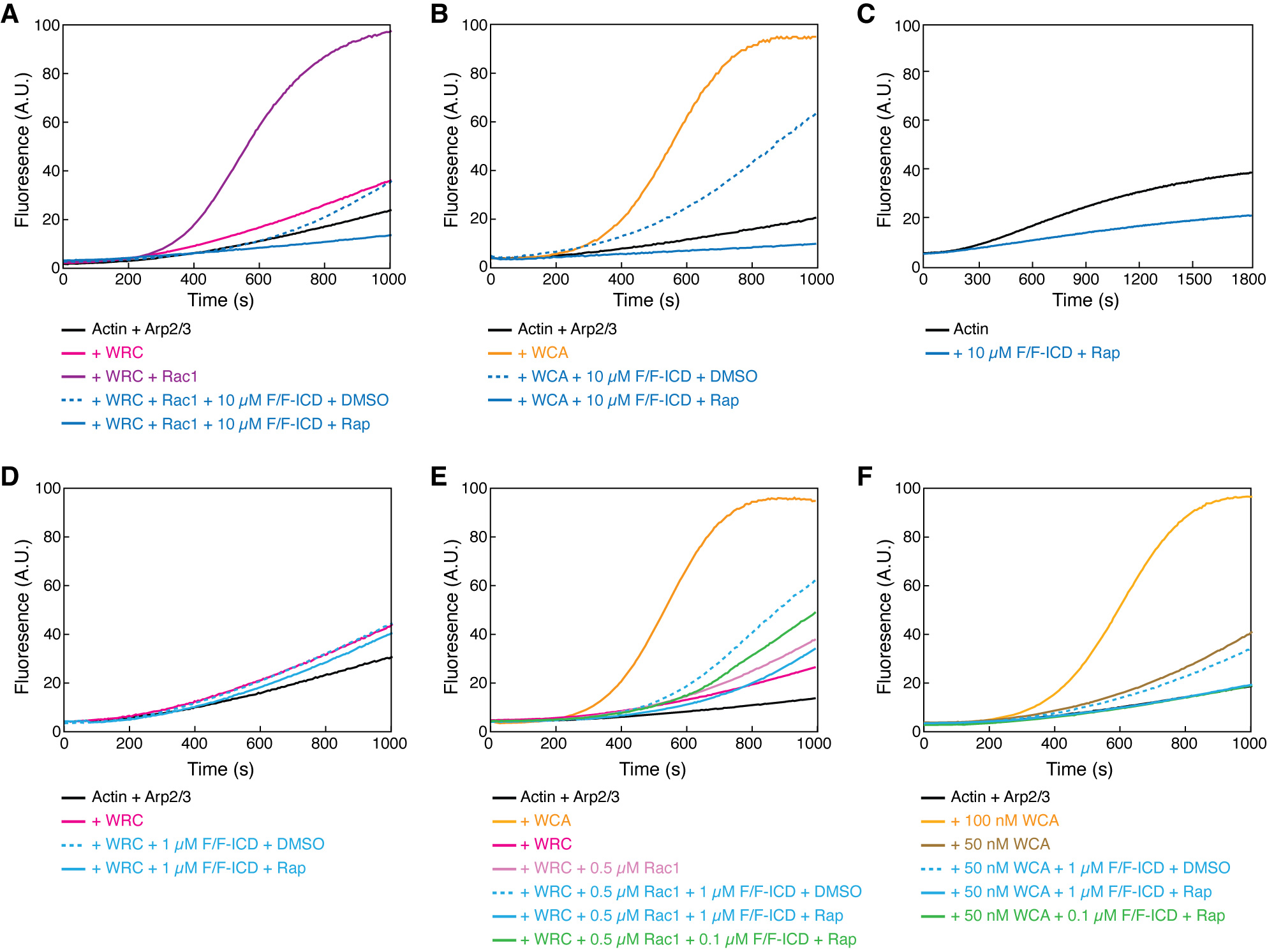

Supplement: Figure 5—source data 1. [file elife-88492-fig5-data1.zip › Figure 5 - source data 1/Figure 5 - unedited.jpg]

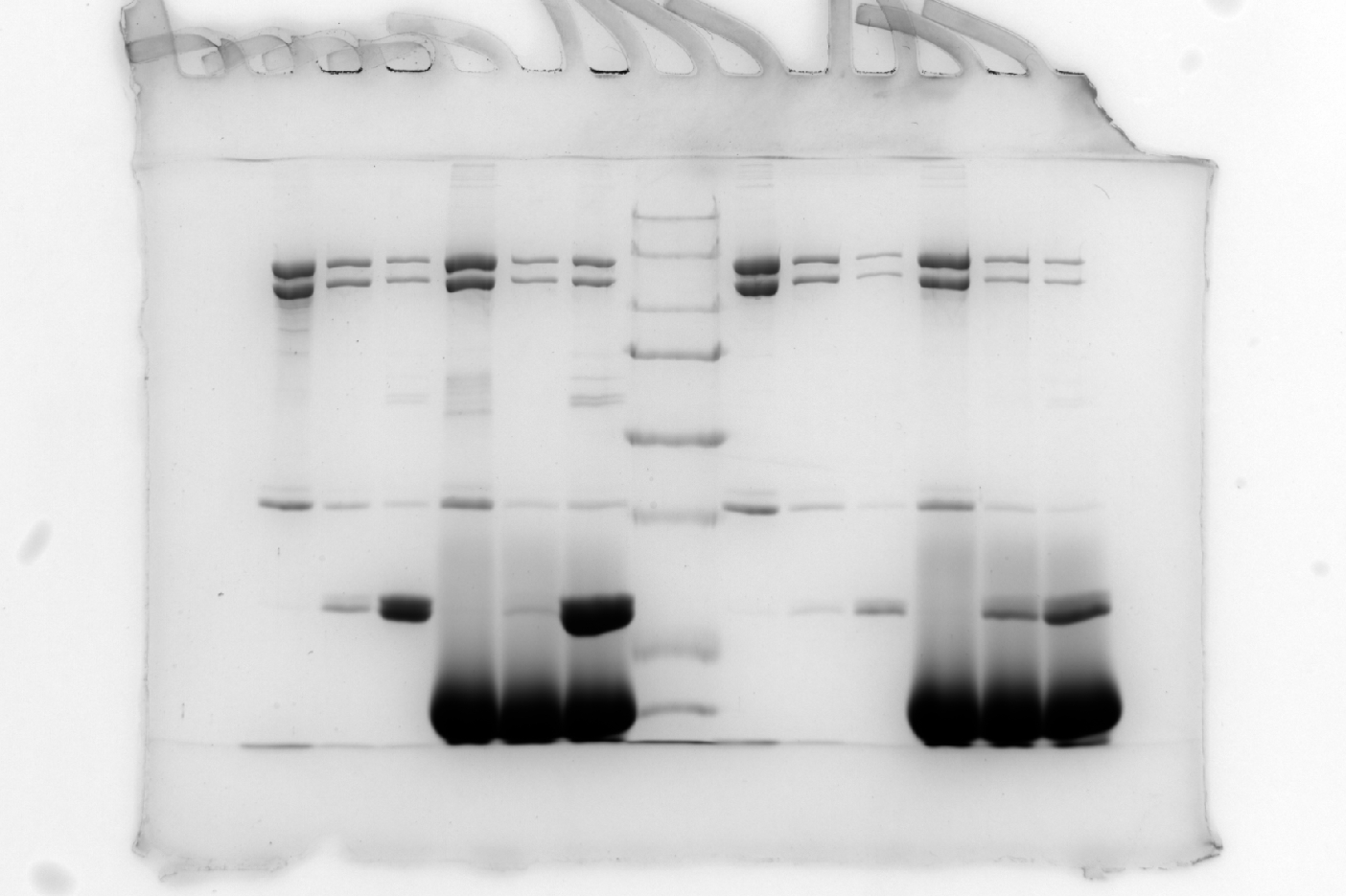

Supplement: Figure 5—figure supplement 1—source data 1. [file elife-88492-fig5-figsupp1-data1.zip › Figure 5 - figure supplement 1 - source data 1/Figure 5 - figure supplement 1G raw image.jpg]

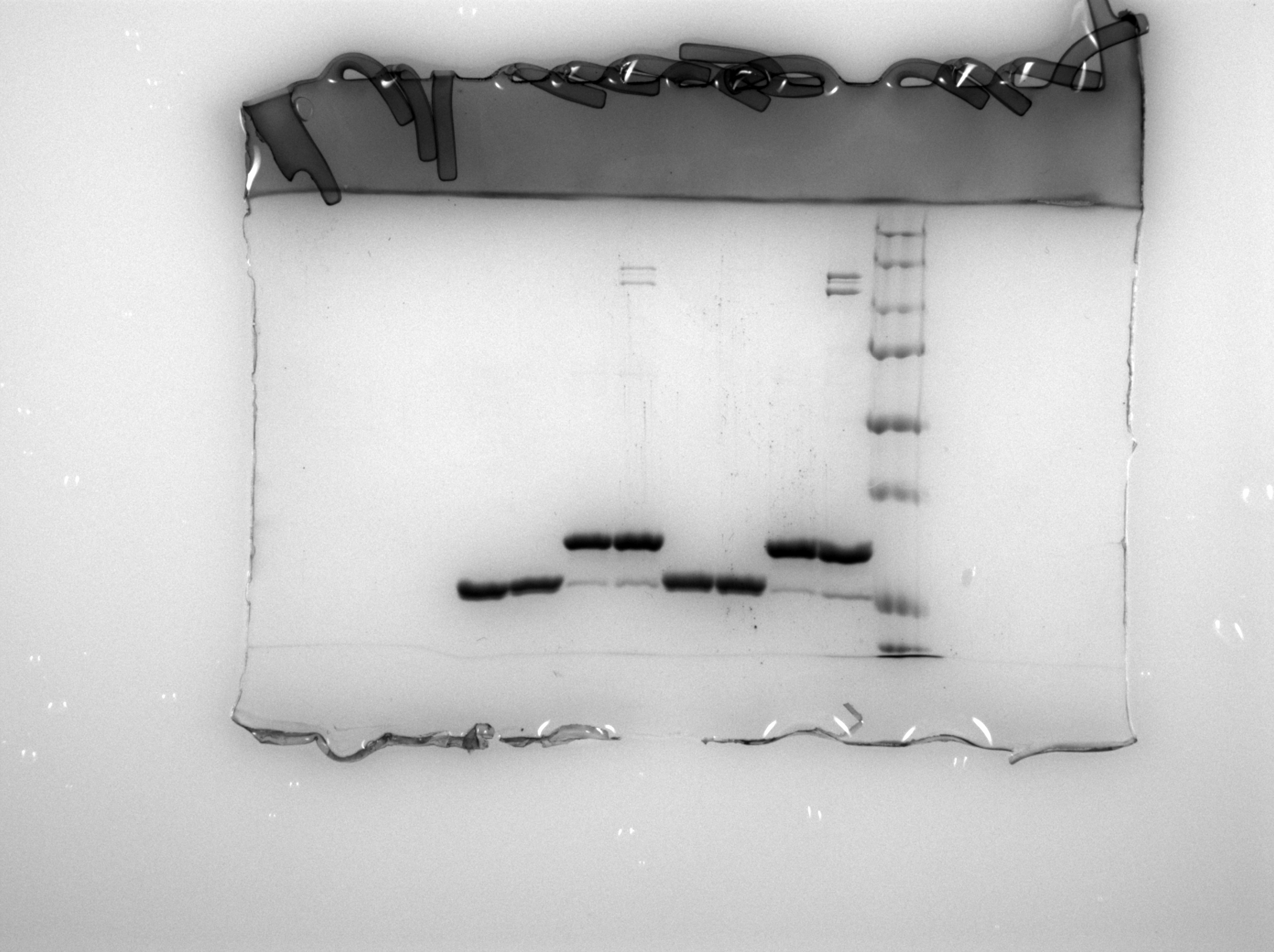

Supplement: Figure 5—figure supplement 1—source data 1. [file elife-88492-fig5-figsupp1-data1.zip › Figure 5 - figure supplement 1 - source data 1/Figure 5 - figure Supplement 1A right raw image.jpg]

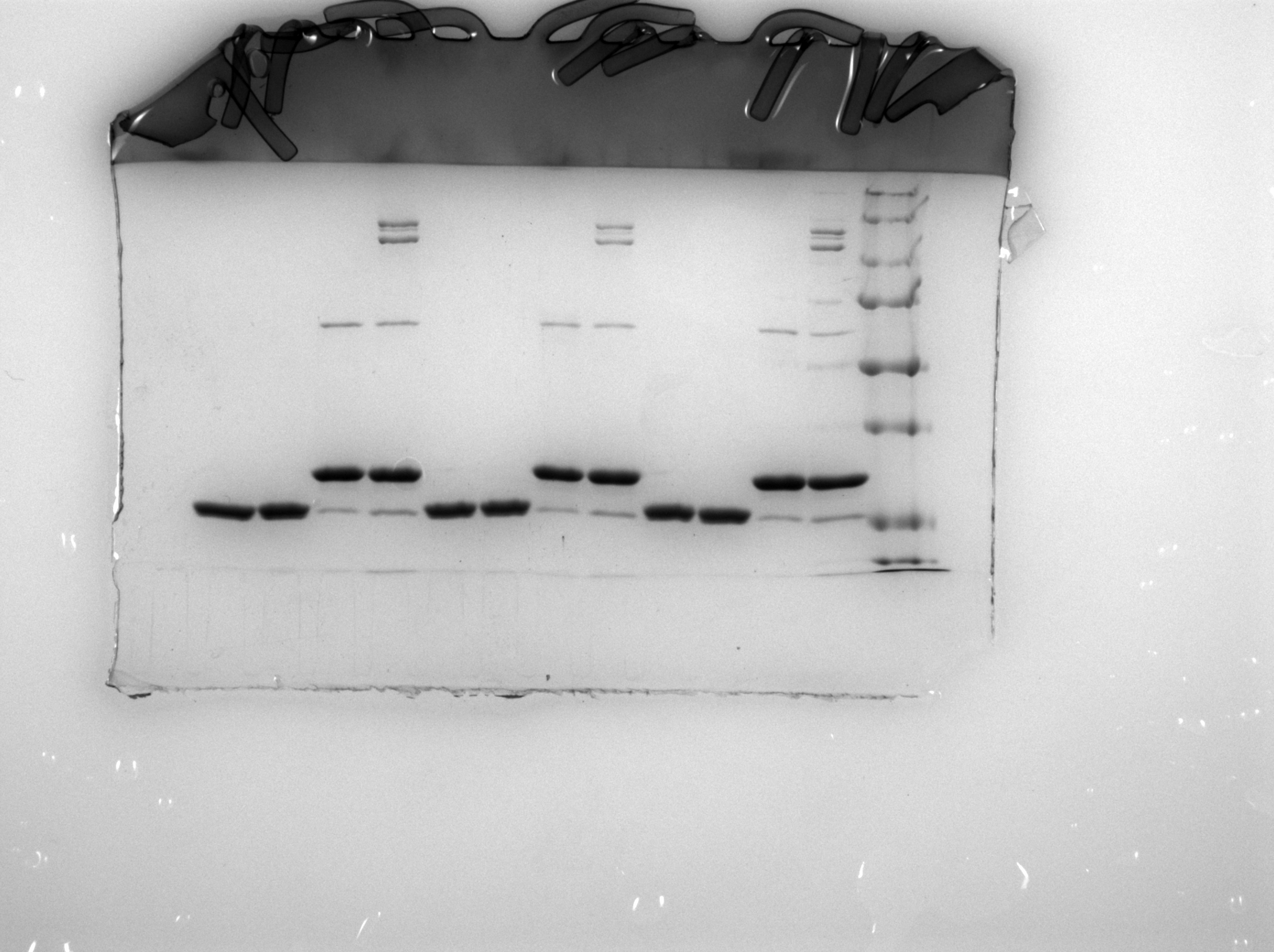

Supplement: Figure 5—figure supplement 1—source data 1. [file elife-88492-fig5-figsupp1-data1.zip › Figure 5 - figure supplement 1 - source data 1/Figure 5 - figure Supplement 1A left raw image.jpg]

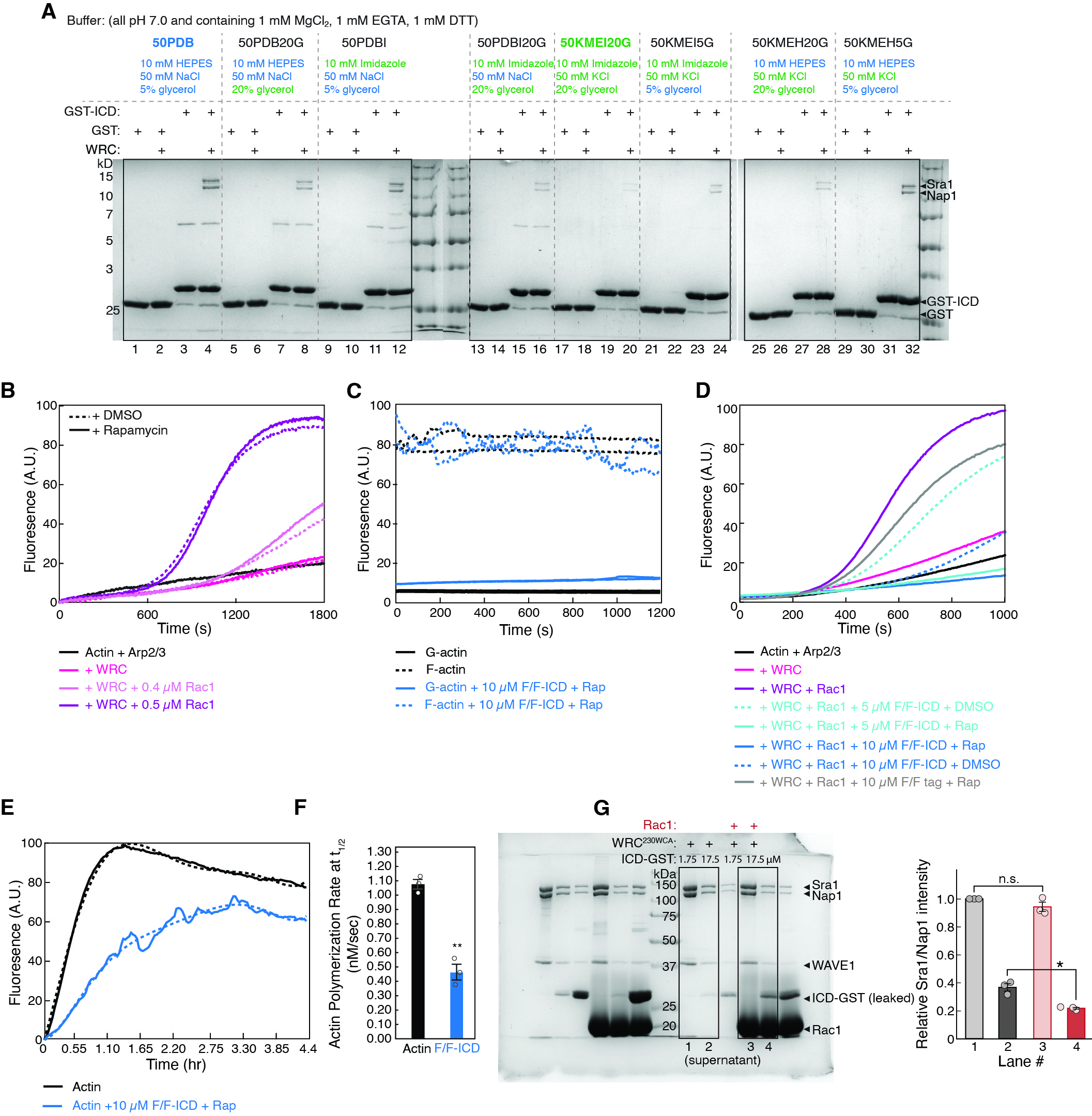

Supplement: Figure 5—figure supplement 1—source data 1. [file elife-88492-fig5-figsupp1-data1.zip › Figure 5 - figure supplement 1 - source data 1/Figure 5 - figure supplement 1 unedited.jpg]

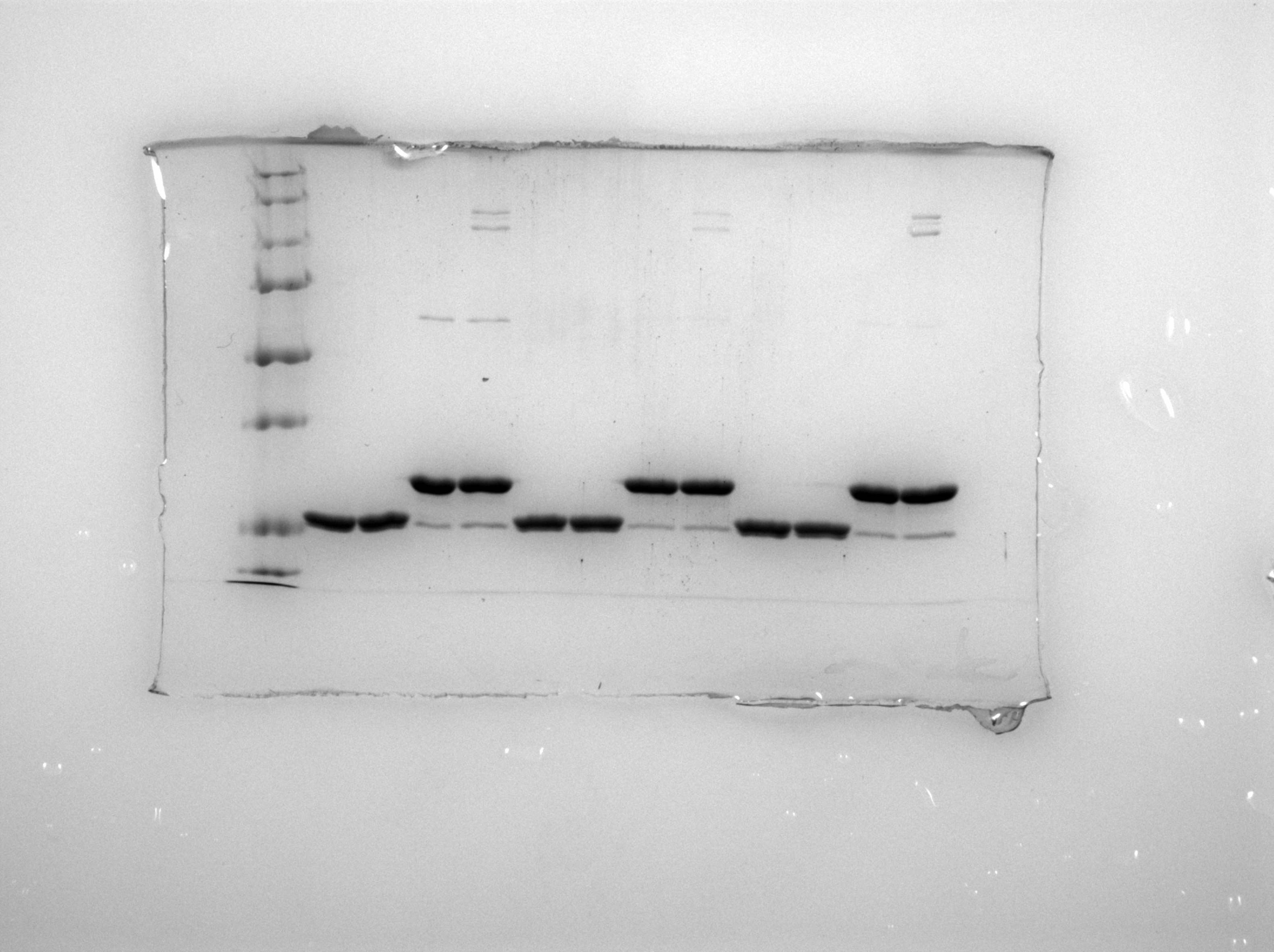

Supplement: Figure 5—figure supplement 1—source data 1. [file elife-88492-fig5-figsupp1-data1.zip › Figure 5 - figure supplement 1 - source data 1/Figure 5 - figure Supplement 1A middle raw image.jpg]

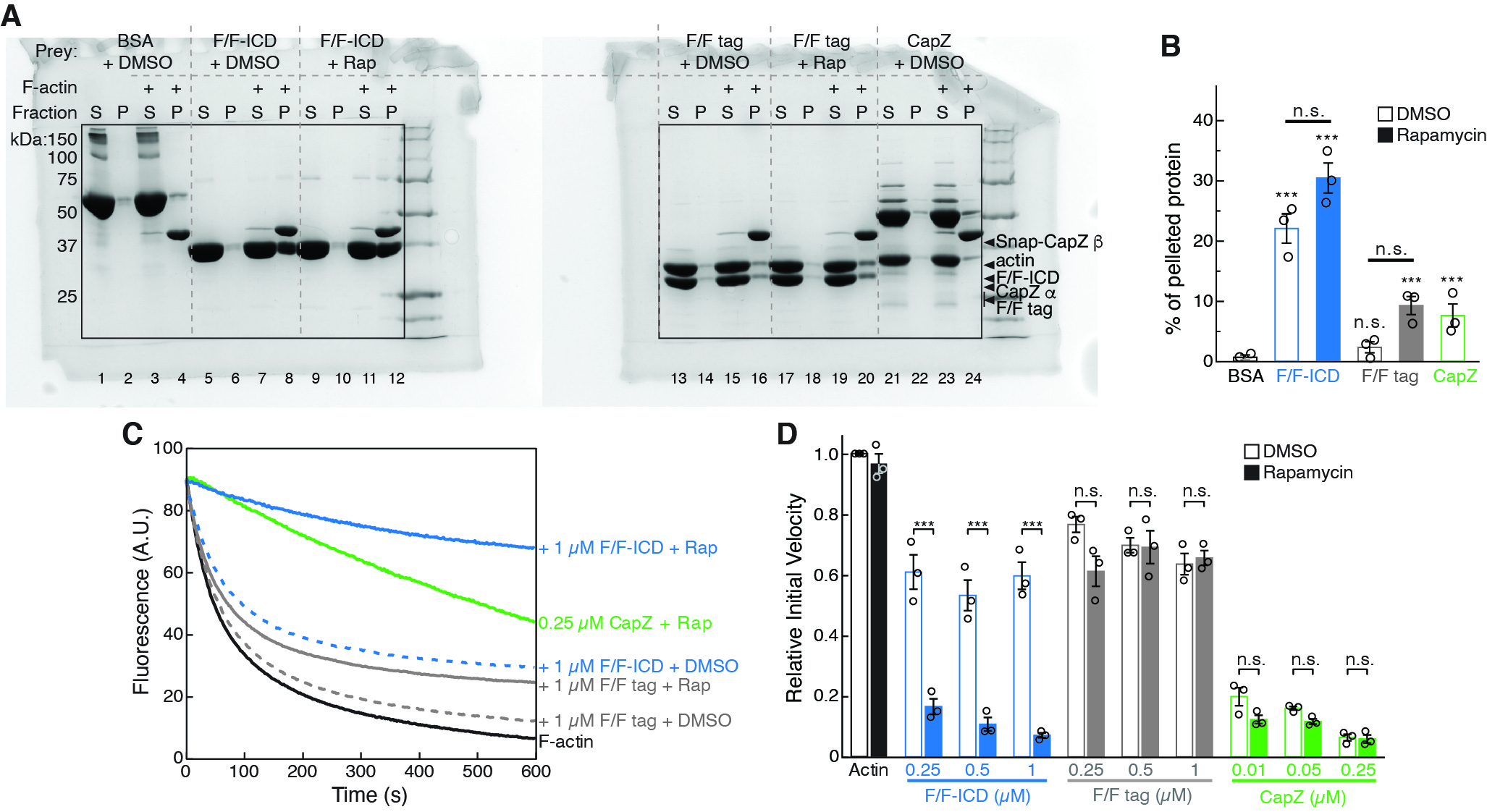

Supplement: Figure 6—source data 1. [file elife-88492-fig6-data1.zip › Figure 6 - source data 1/Figure 6 Unedited.jpg]

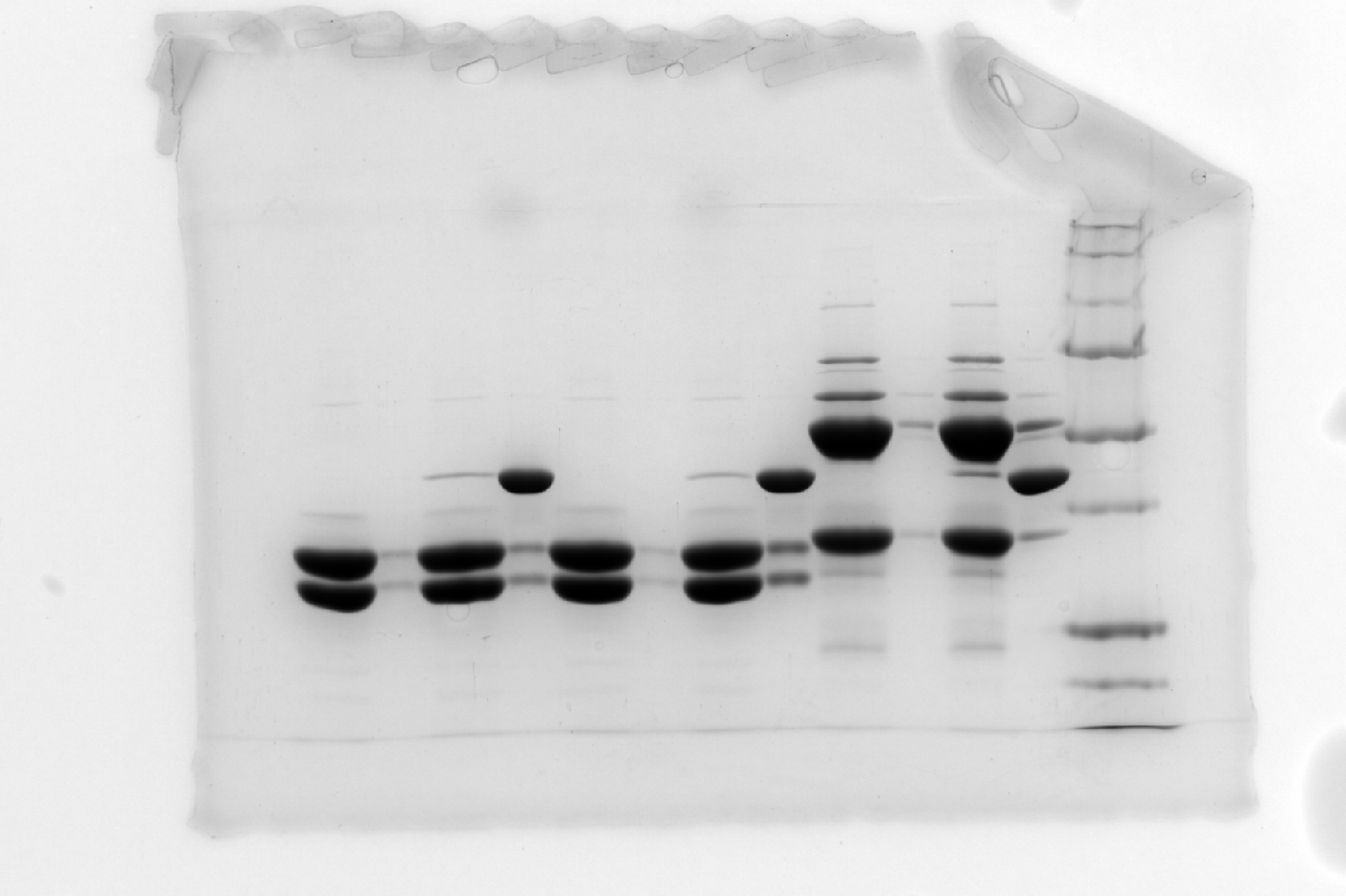

Supplement: Figure 6—source data 1. [file elife-88492-fig6-data1.zip › Figure 6 - source data 1/Figure 6A right raw image.jpg]

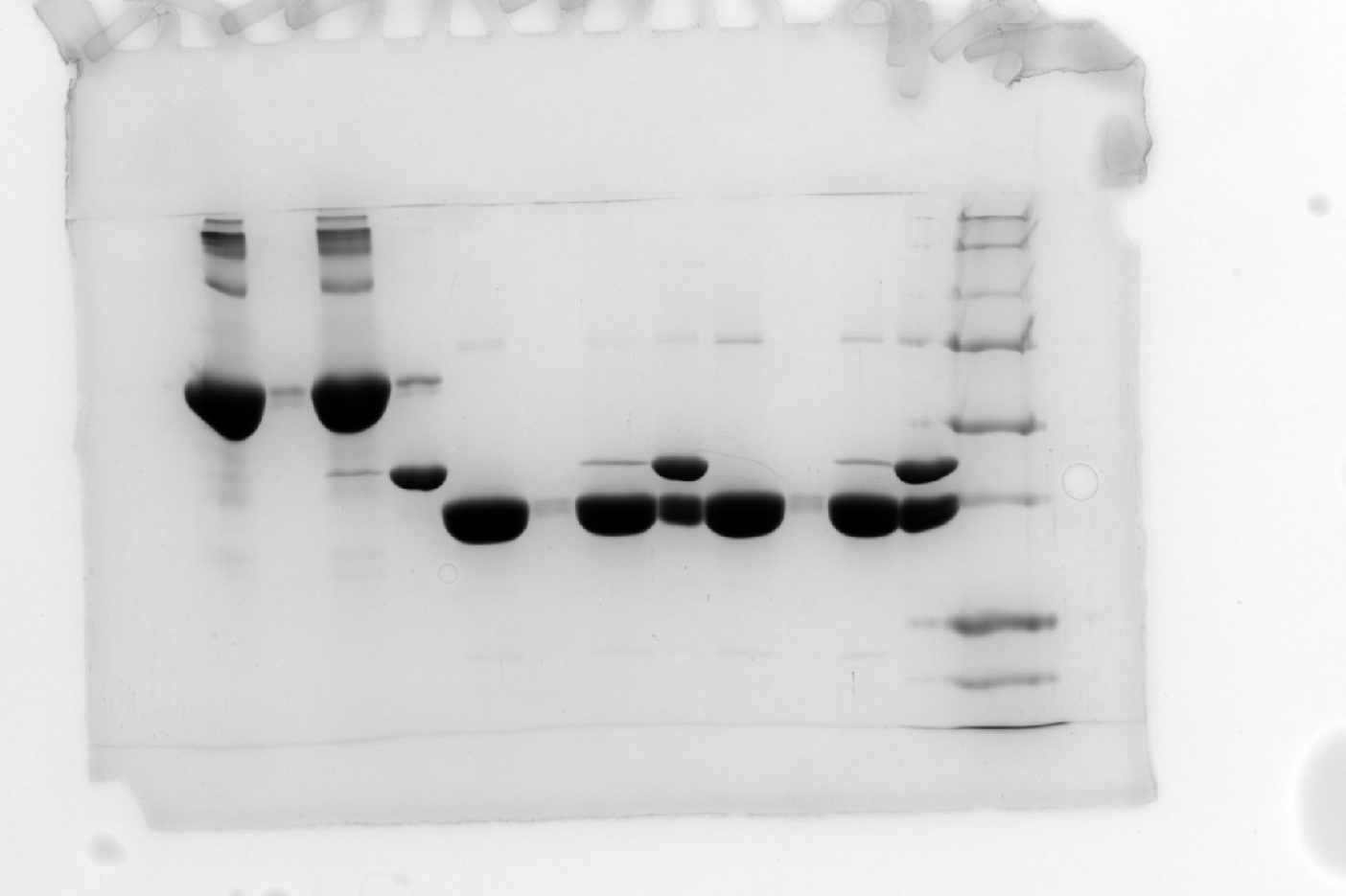

Supplement: Figure 6—source data 1. [file elife-88492-fig6-data1.zip › Figure 6 - source data 1/Figure 6A left raw image.jpg]

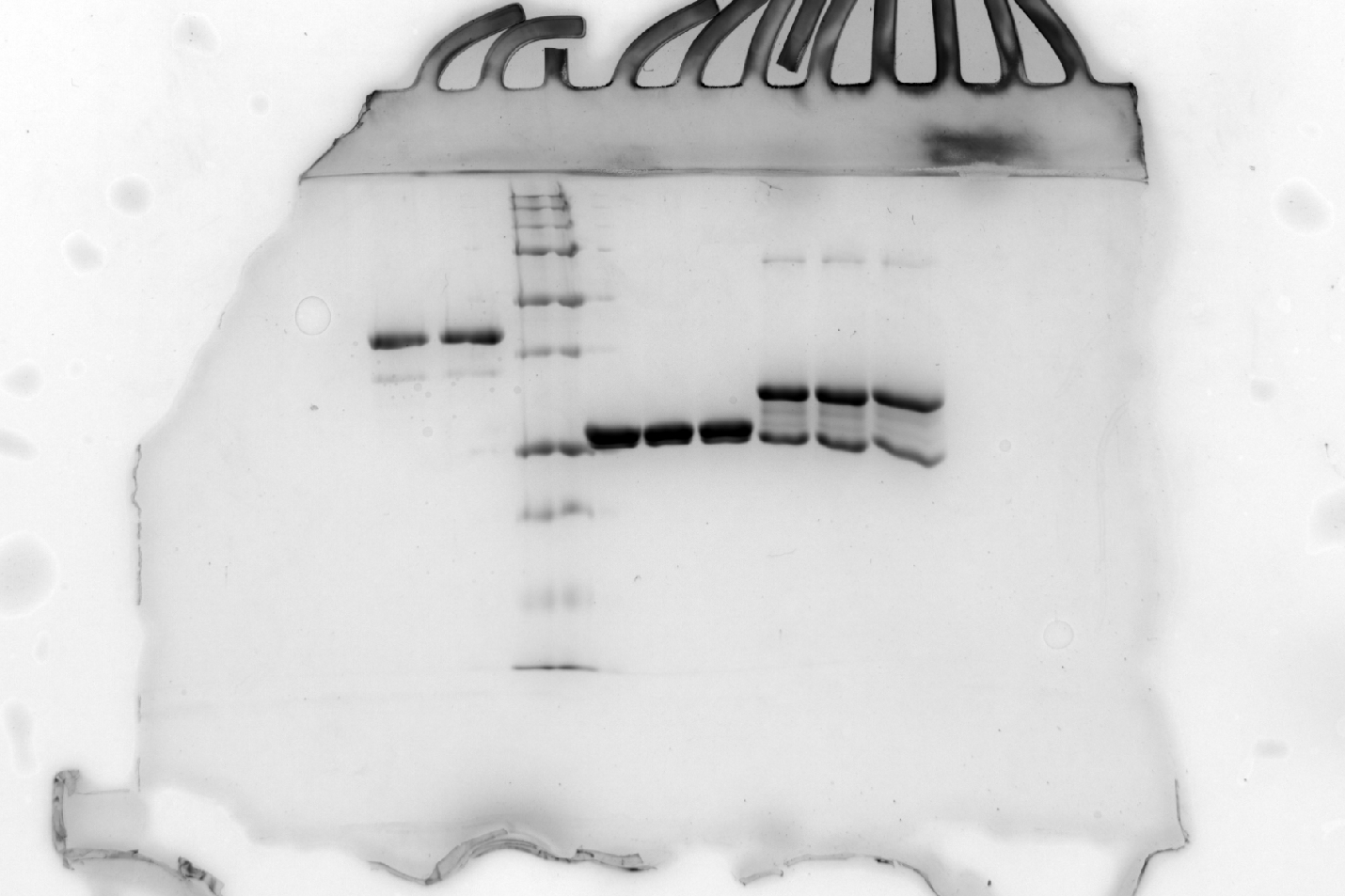

Supplement: Figure 6—figure supplement 1—source data 1. [file elife-88492-fig6-figsupp1-data1.zip › Figure 6 - figure supplement 1 - source data 1/Figure 6 - figure supplement 1A raw image.jpg]

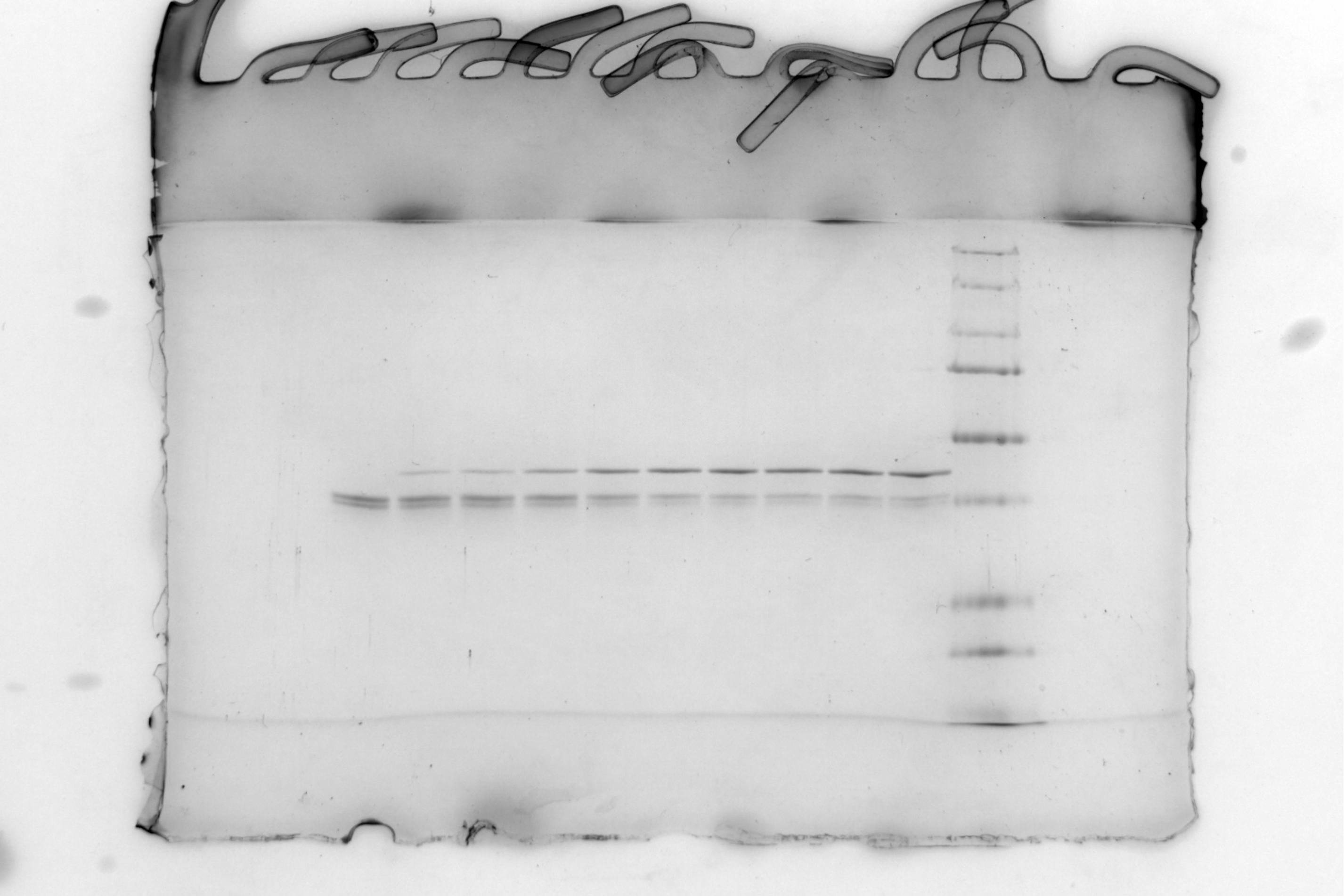

Supplement: Figure 6—figure supplement 1—source data 1. [file elife-88492-fig6-figsupp1-data1.zip › Figure 6 - figure supplement 1 - source data 1/Figure 6 - figure supplement 1C raw gel image.tif]

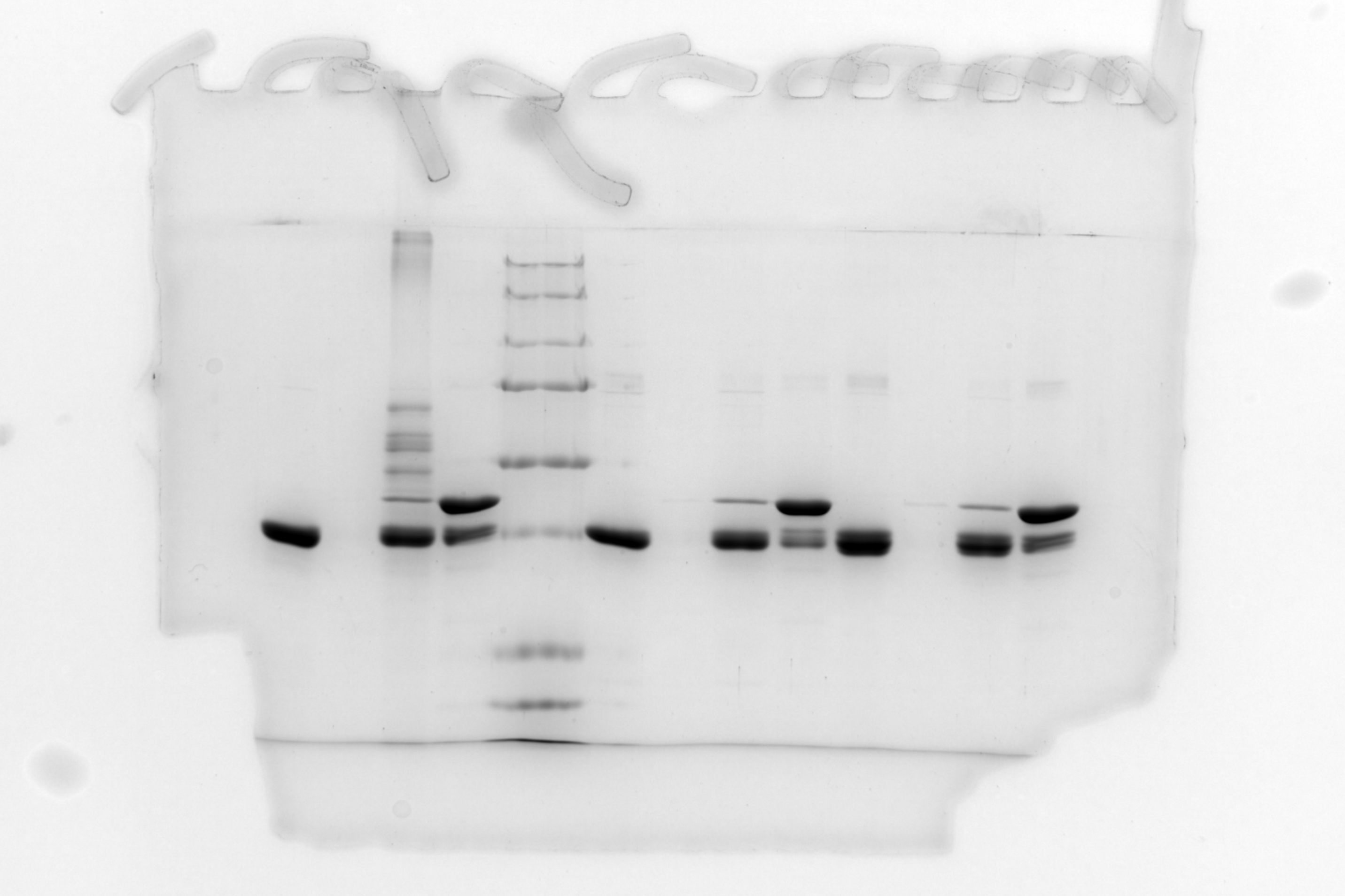

Supplement: Figure 6—figure supplement 1—source data 1. [file elife-88492-fig6-figsupp1-data1.zip › Figure 6 - figure supplement 1 - source data 1/Figure 6 - figure supplement 1D raw gel (lanes 13-24).tif]

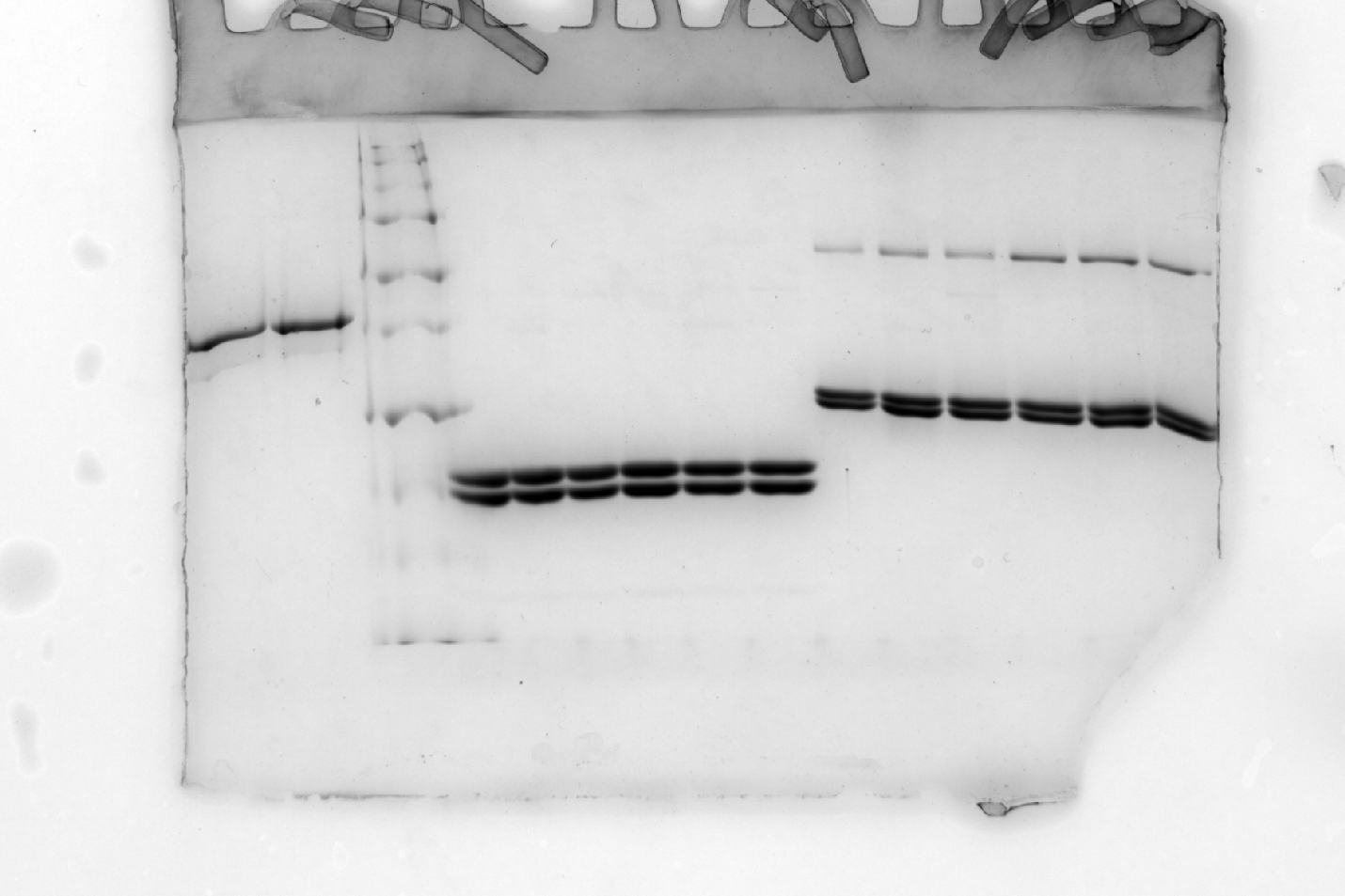

Supplement: Figure 6—figure supplement 1—source data 1. [file elife-88492-fig6-figsupp1-data1.zip › Figure 6 - figure supplement 1 - source data 1/Figure 6 - figure supplement 1B raw image.jpg]

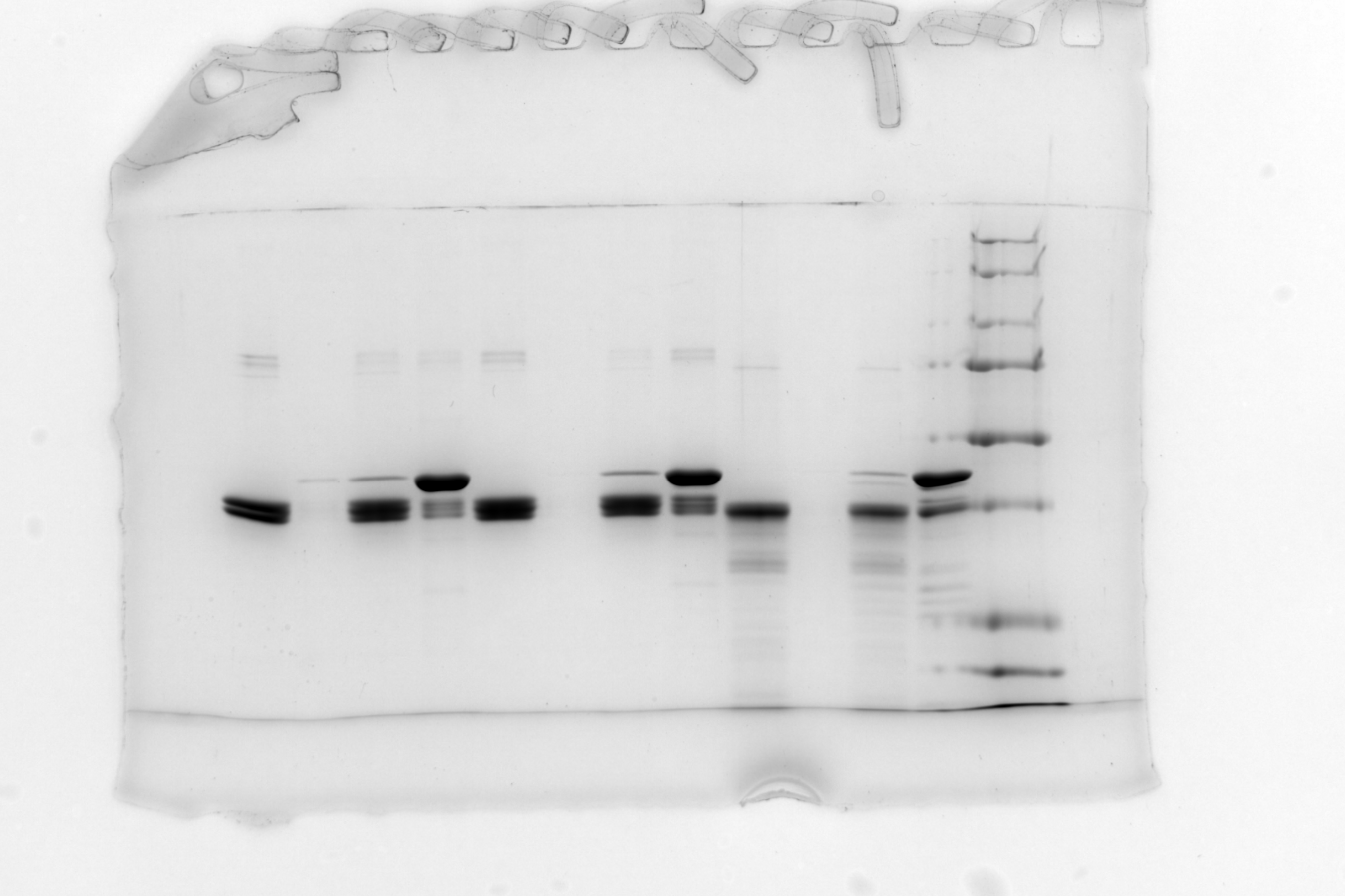

Supplement: Figure 6—figure supplement 1—source data 1. [file elife-88492-fig6-figsupp1-data1.zip › Figure 6 - figure supplement 1 - source data 1/Figure 6 - figure supplement 1D raw gel (lanes 37-48).tif]

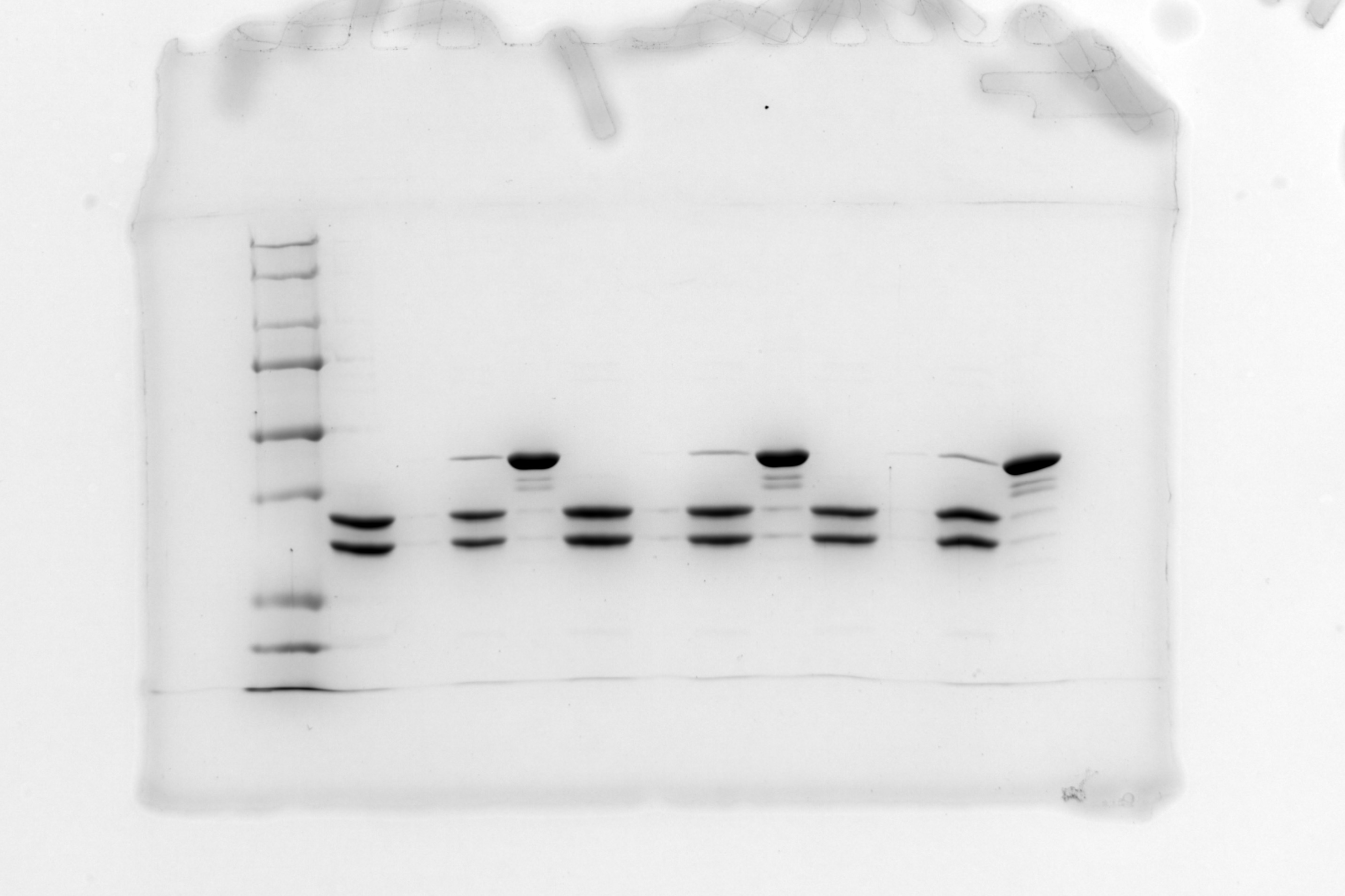

Supplement: Figure 6—figure supplement 1—source data 1. [file elife-88492-fig6-figsupp1-data1.zip › Figure 6 - figure supplement 1 - source data 1/Figure 6 - figure supplement 1D raw gel (lanes 49-52).tif]

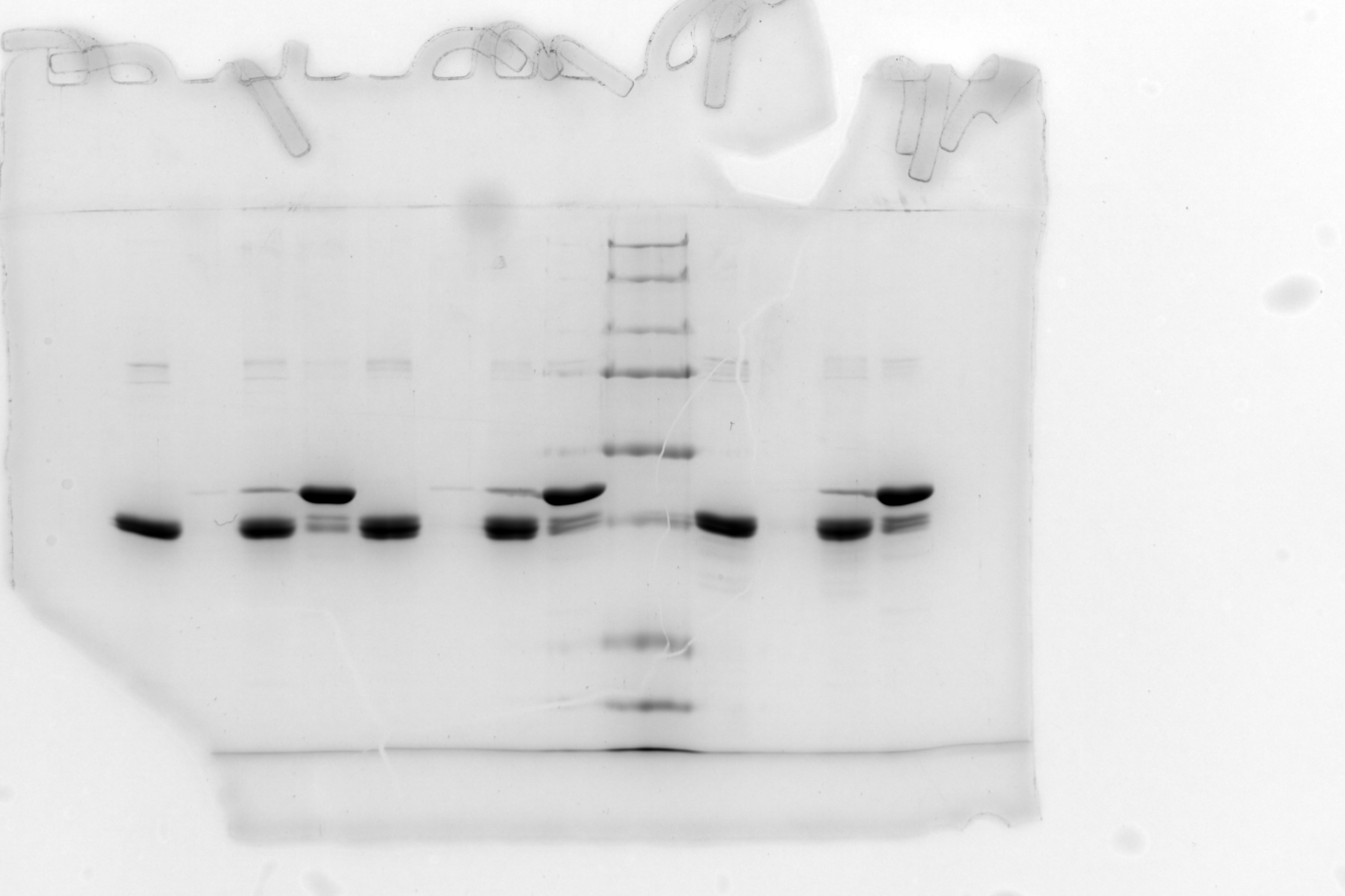

Supplement: Figure 6—figure supplement 1—source data 1. [file elife-88492-fig6-figsupp1-data1.zip › Figure 6 - figure supplement 1 - source data 1/Figure 6 - figure supplement 1D raw gel (lanes 25-36).tif]

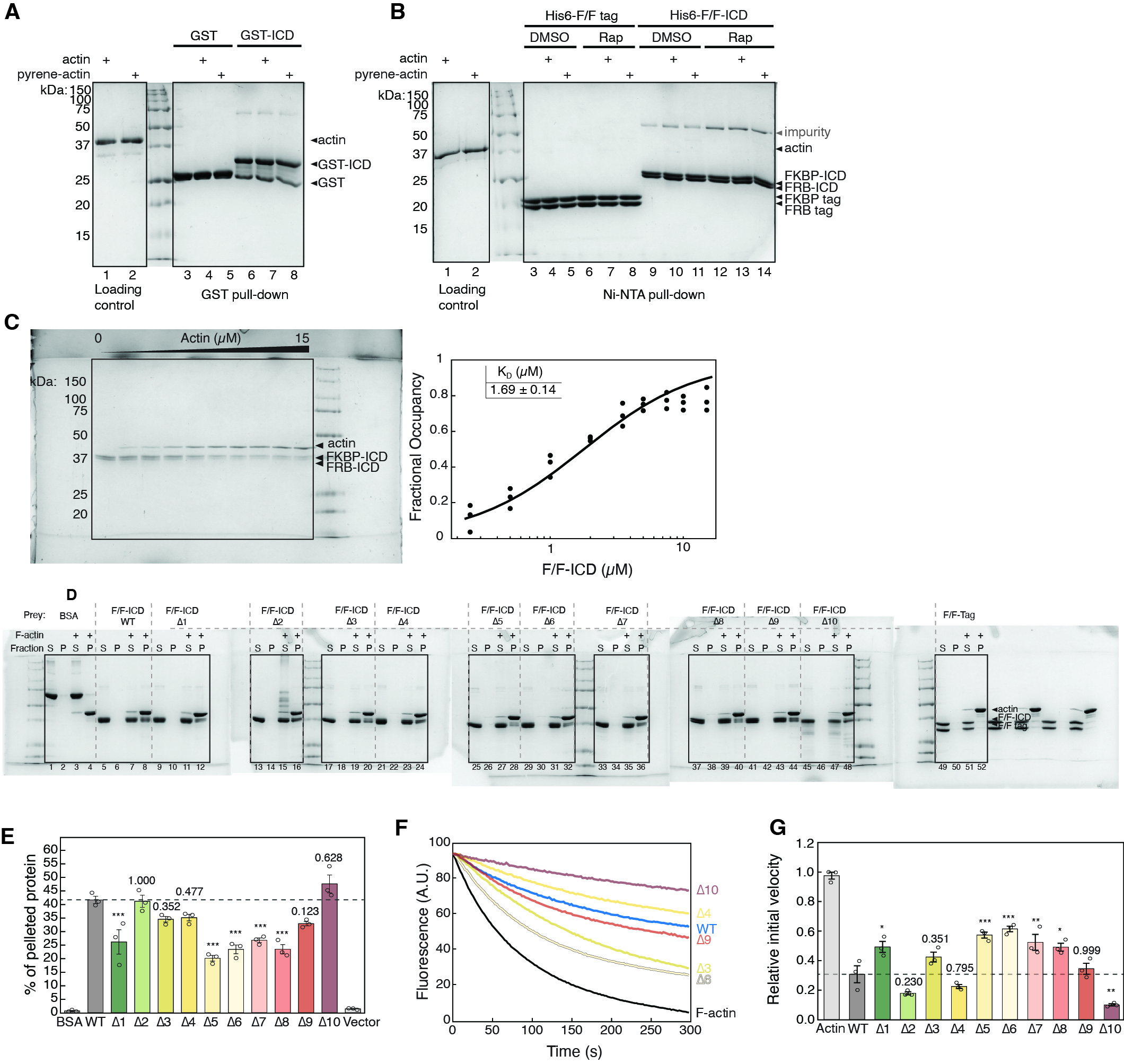

Supplement: Figure 6—figure supplement 1—source data 1. [file elife-88492-fig6-figsupp1-data1.zip › Figure 6 - figure supplement 1 - source data 1/Figure 6 - figure supplement 1 - unedited.jpg]

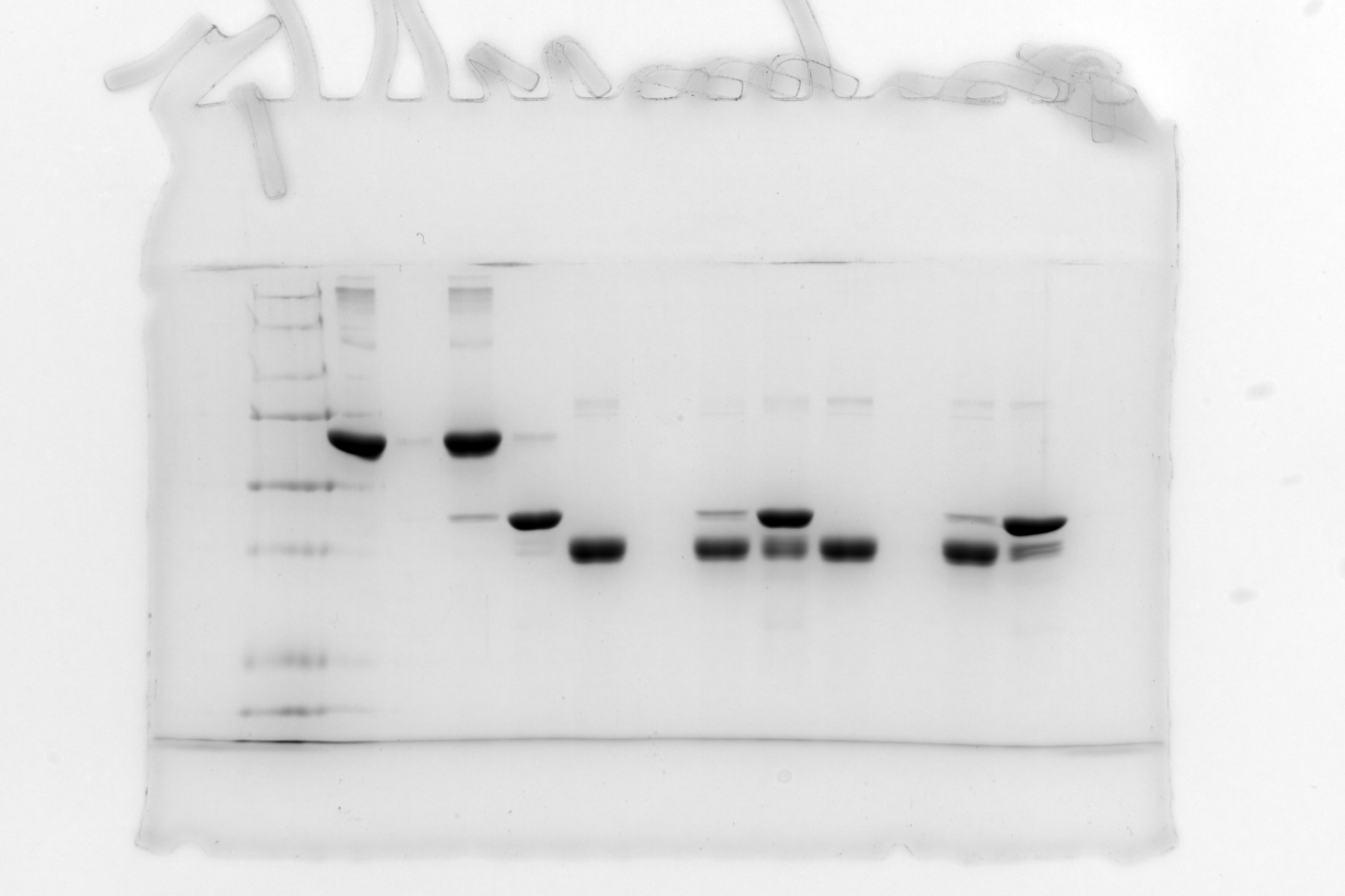

Supplement: Figure 6—figure supplement 1—source data 1. [file elife-88492-fig6-figsupp1-data1.zip › Figure 6 - figure supplement 1 - source data 1/Figure 6 - figure supplement 1D raw gel (lanes 1-12).tif]

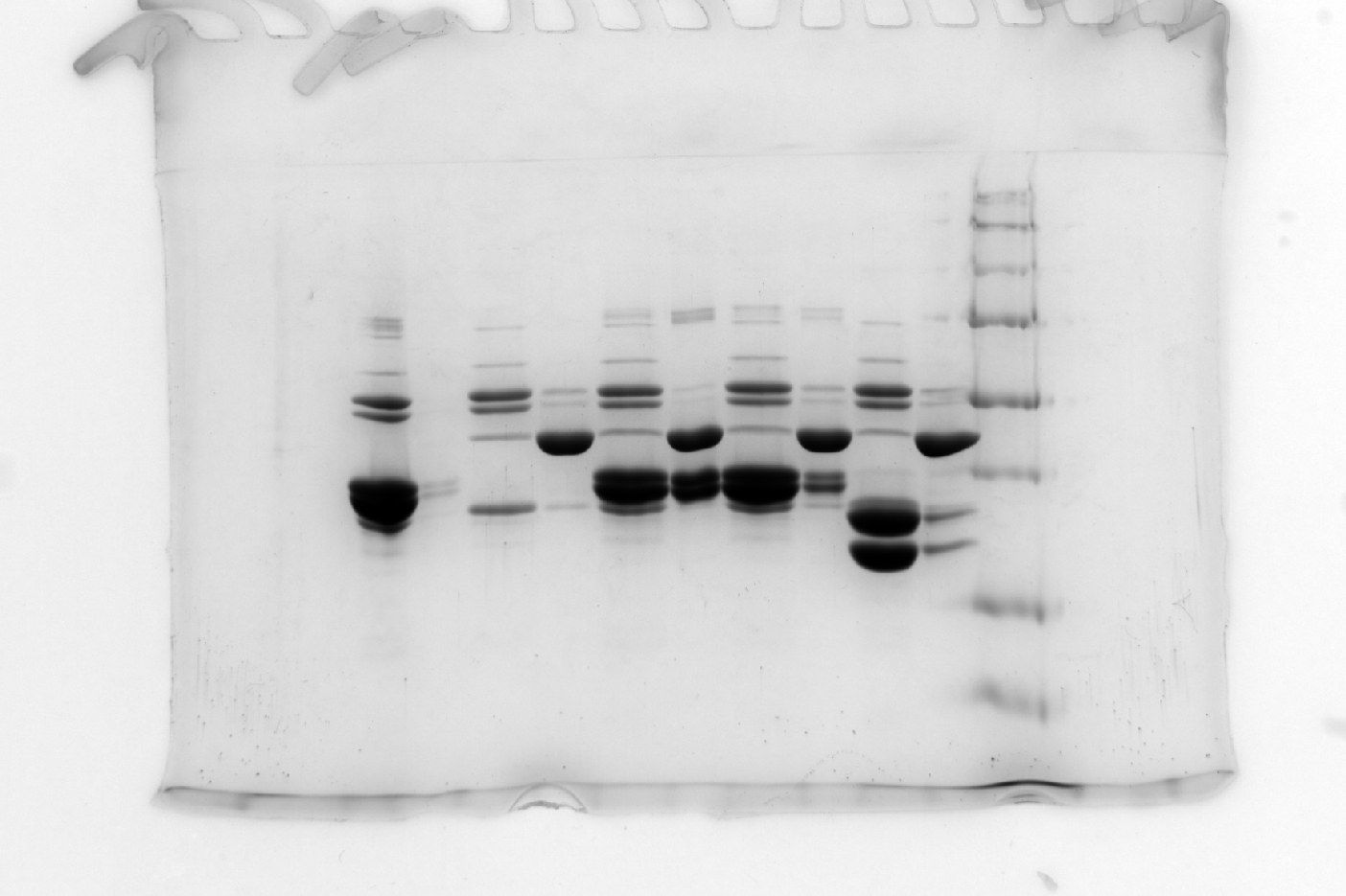

Supplement: Figure 7—source data 1. [file elife-88492-fig7-data1.zip › Figure 7 - source data 1/Figure 7D raw image.jpg]

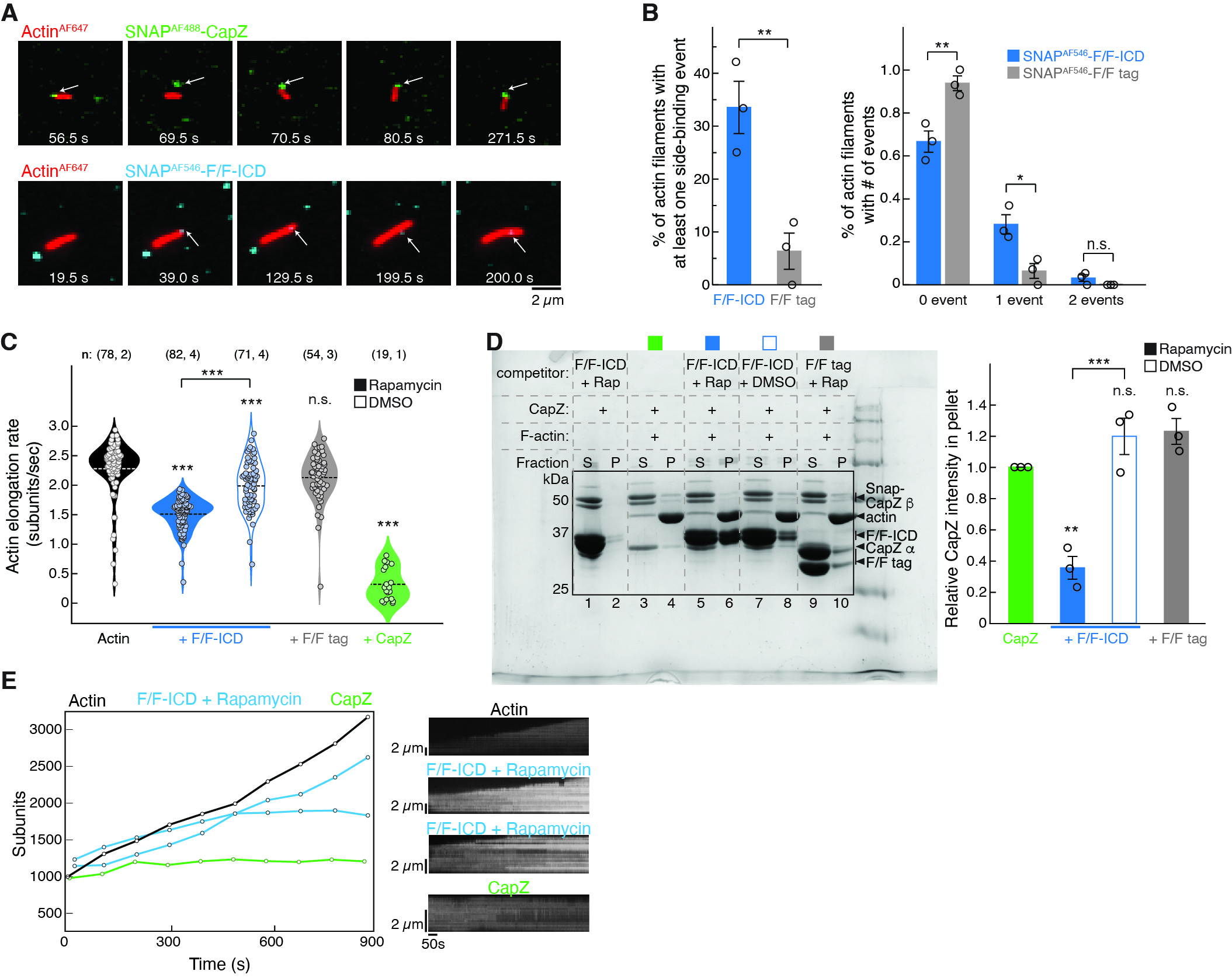

Supplement: Figure 7—source data 1. [file elife-88492-fig7-data1.zip › Figure 7 - source data 1/Figure 7 - unedited.jpg]

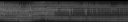

Supplement: Figure 7—source data 3. [file elife-88492-fig7-data3.zip › Figure 7 - source data 3/CapZ kymograph #5.tif]

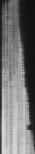

Supplement: Figure 7—source data 3. [file elife-88492-fig7-data3.zip › Figure 7 - source data 3/FFICD rap kymograph #3.tif]

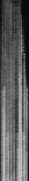

Supplement: Figure 7—source data 3. [file elife-88492-fig7-data3.zip › Figure 7 - source data 3/FFICD rap kymograph #5.tif]

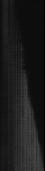

Supplement: Figure 7—source data 3. [file elife-88492-fig7-data3.zip › Figure 7 - source data 3/Actin kymograph #2.tif]

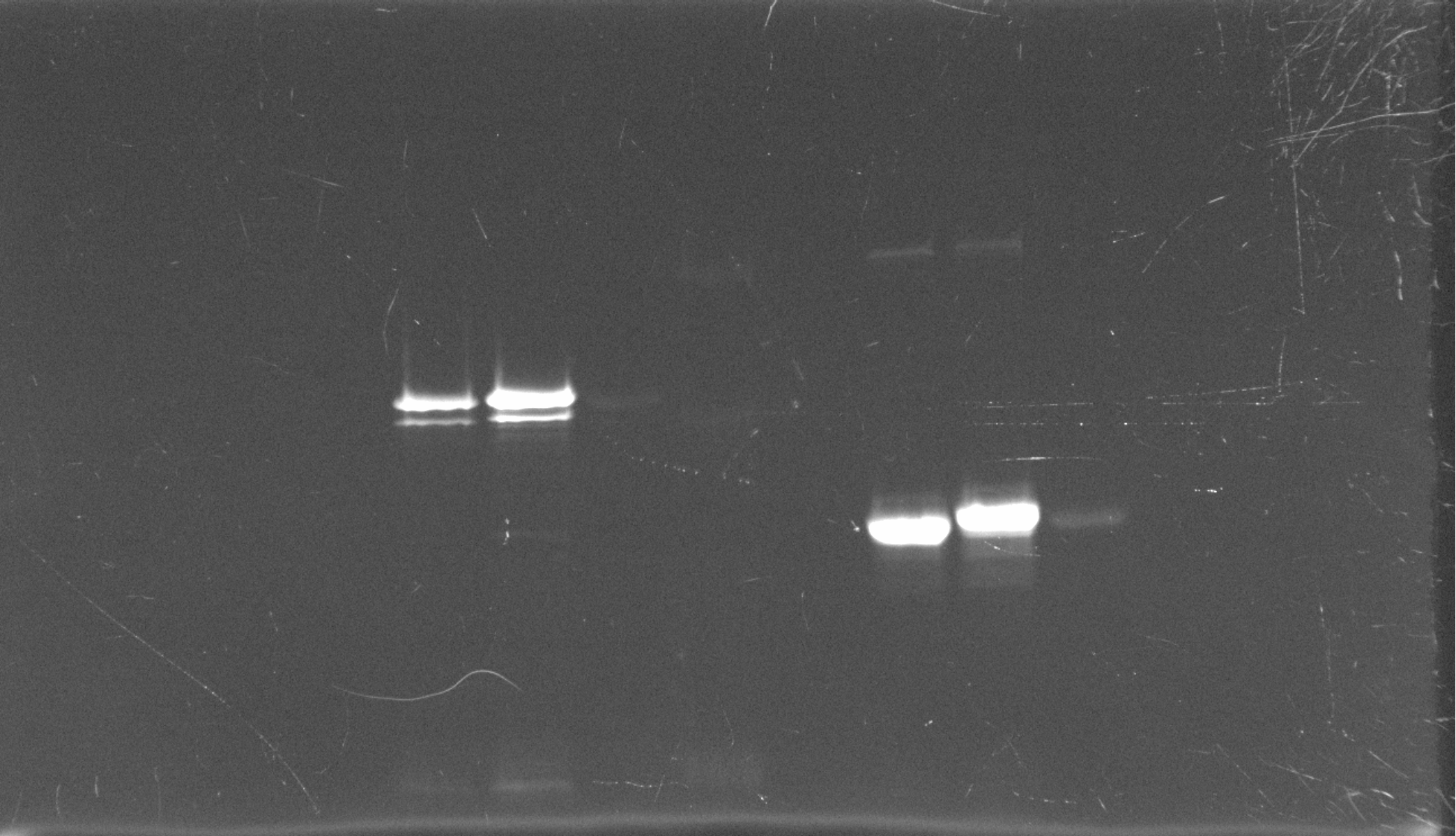

Supplement: Figure 7—figure supplement 1—source data 1. [file elife-88492-fig7-figsupp1-data1.zip › Figure 7 - figure supplement 1 - source data 1/Figure 7ΓÇöfigure supplement 1B Left raw image.tif]

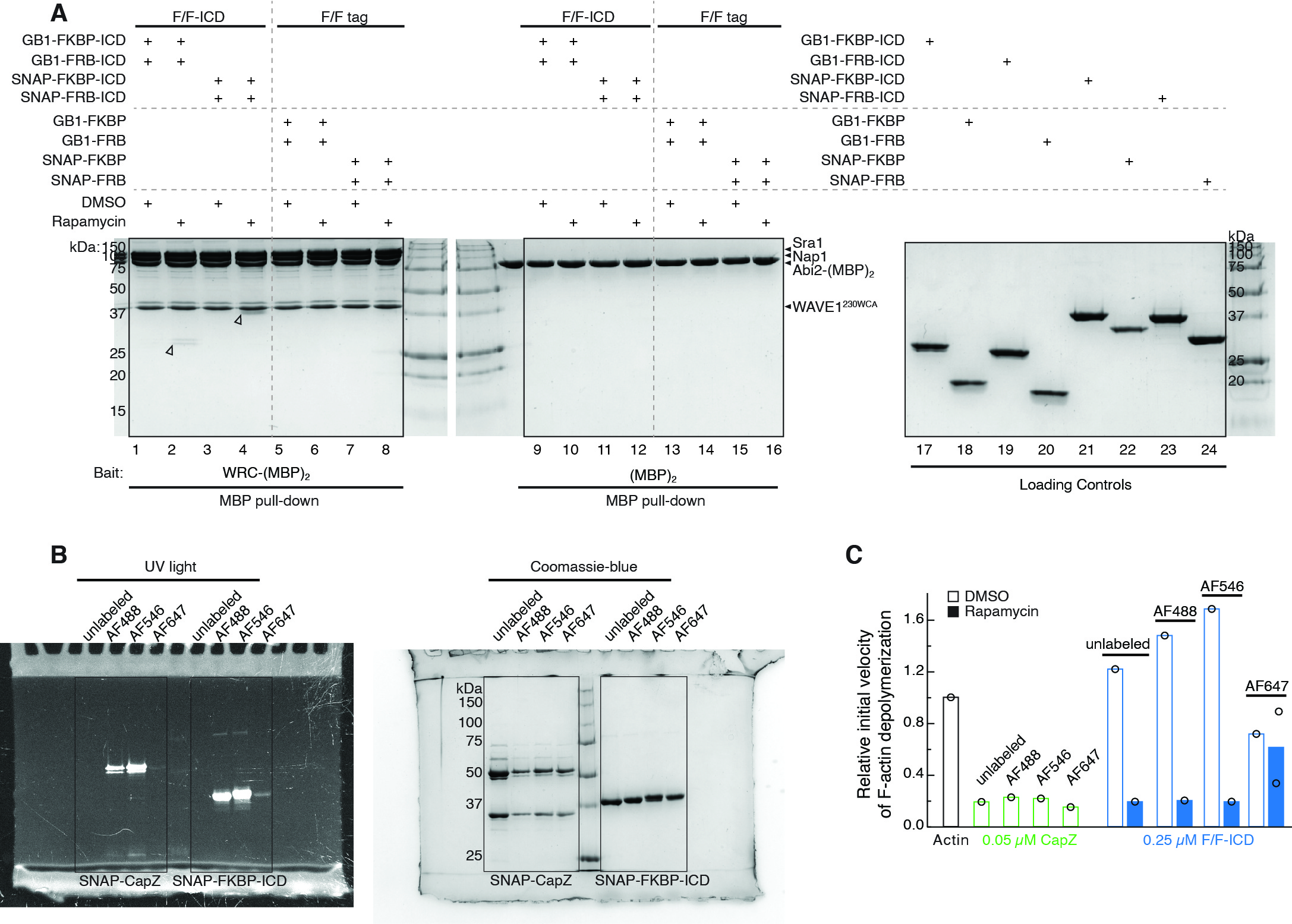

Supplement: Figure 7—figure supplement 1—source data 1. [file elife-88492-fig7-figsupp1-data1.zip › Figure 7 - figure supplement 1 - source data 1/Figure 7ΓÇöfigure supplement 1 Unedited.jpg]

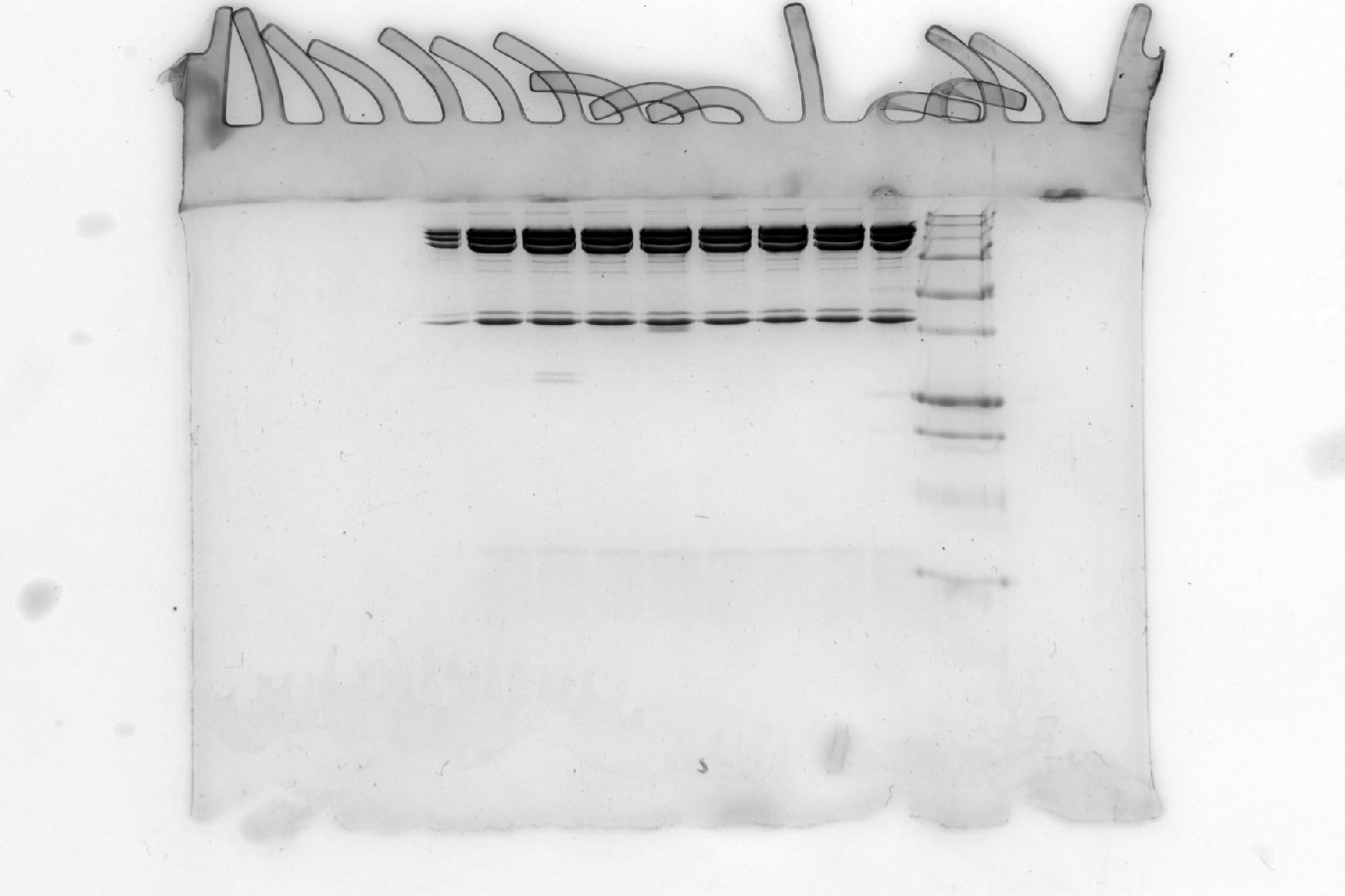

Supplement: Figure 7—figure supplement 1—source data 1. [file elife-88492-fig7-figsupp1-data1.zip › Figure 7 - figure supplement 1 - source data 1/Figure 7ΓÇöfigure supplement 1A left raw image.jpg]

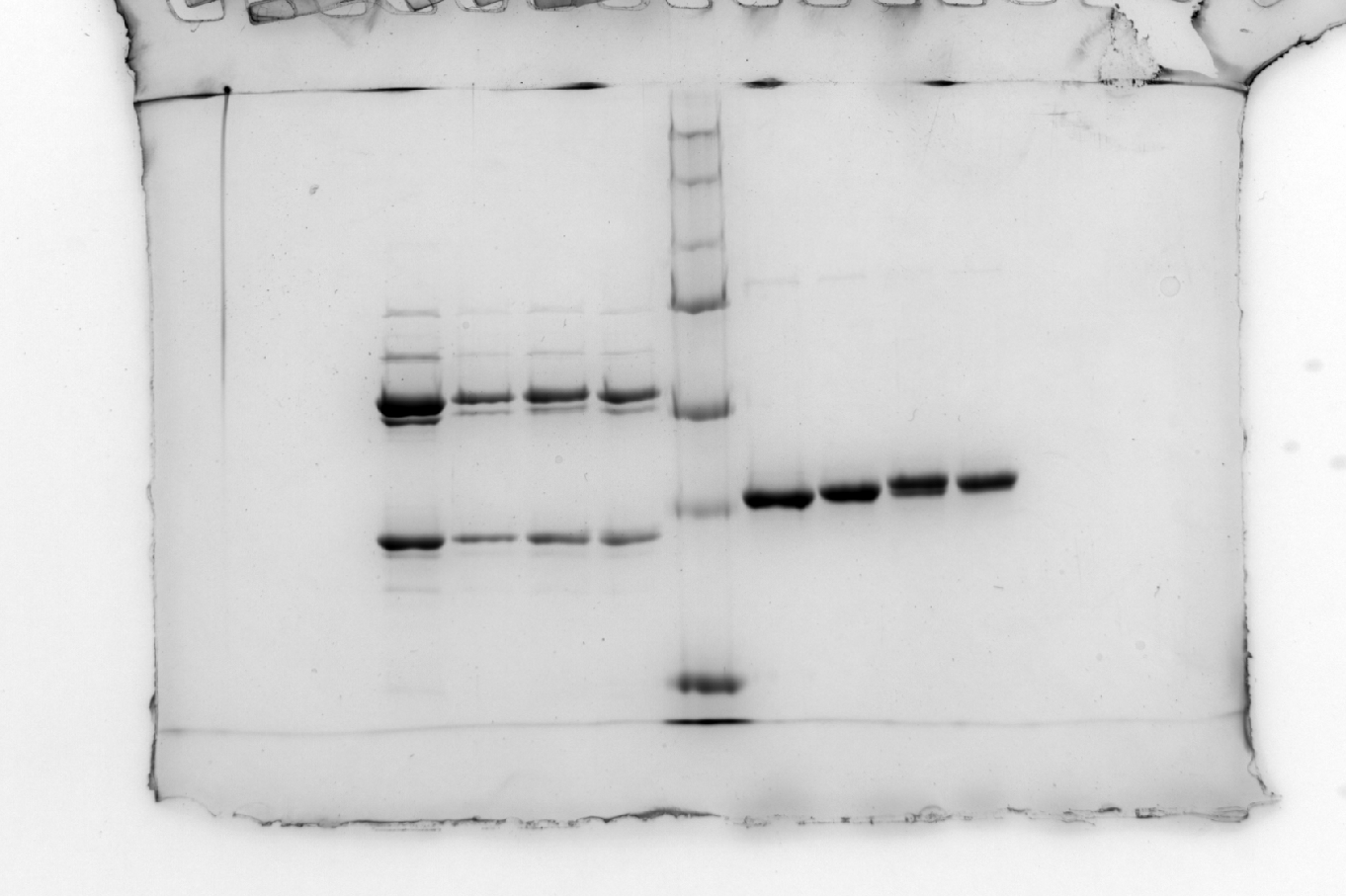

Supplement: Figure 7—figure supplement 1—source data 1. [file elife-88492-fig7-figsupp1-data1.zip › Figure 7 - figure supplement 1 - source data 1/Figure 7ΓÇöfigure supplement 1B Right raw image.jpg]

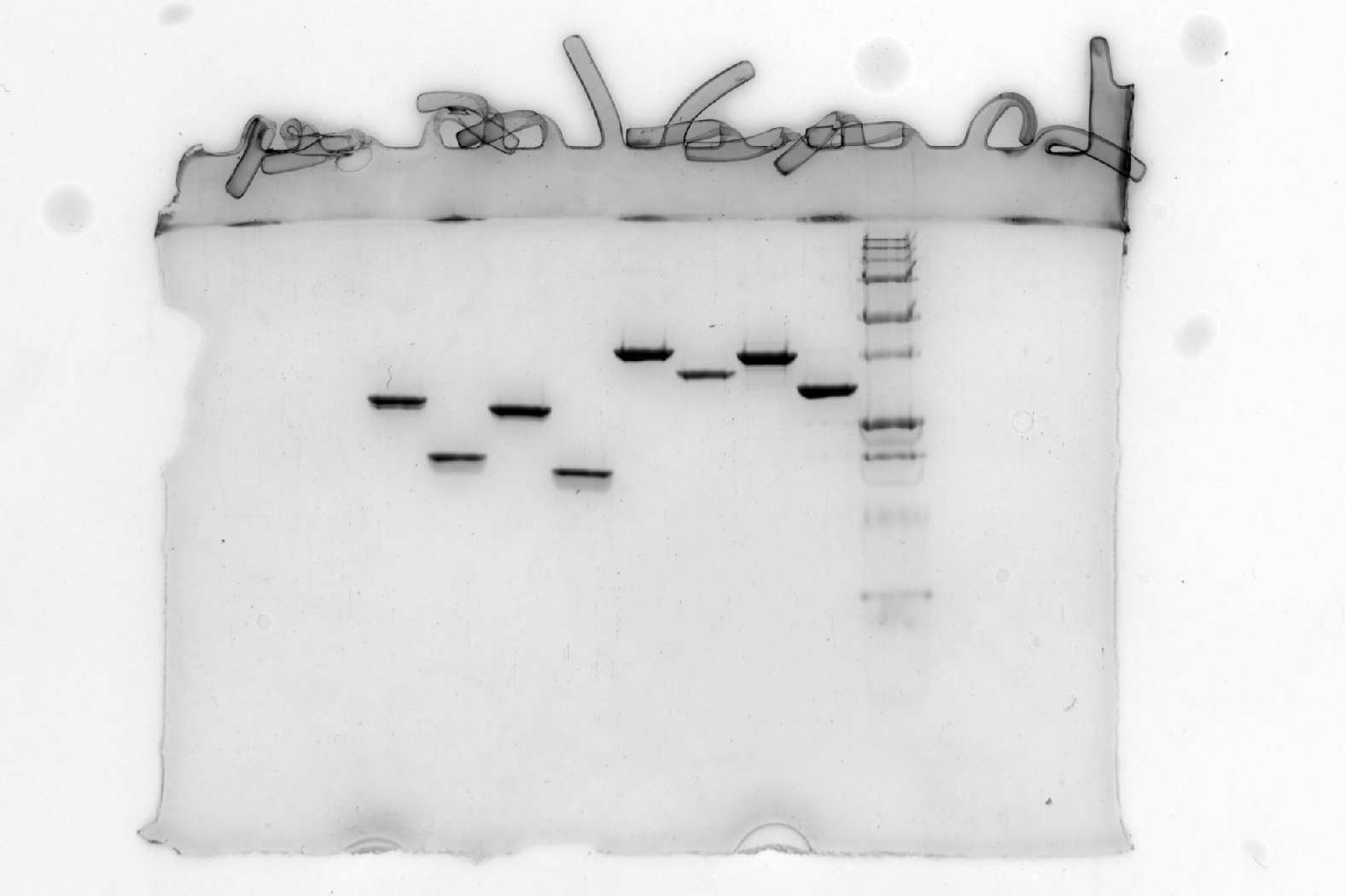

Supplement: Figure 7—figure supplement 1—source data 1. [file elife-88492-fig7-figsupp1-data1.zip › Figure 7 - figure supplement 1 - source data 1/Figure 7ΓÇöfigure supplement 1A right raw image.jpg]

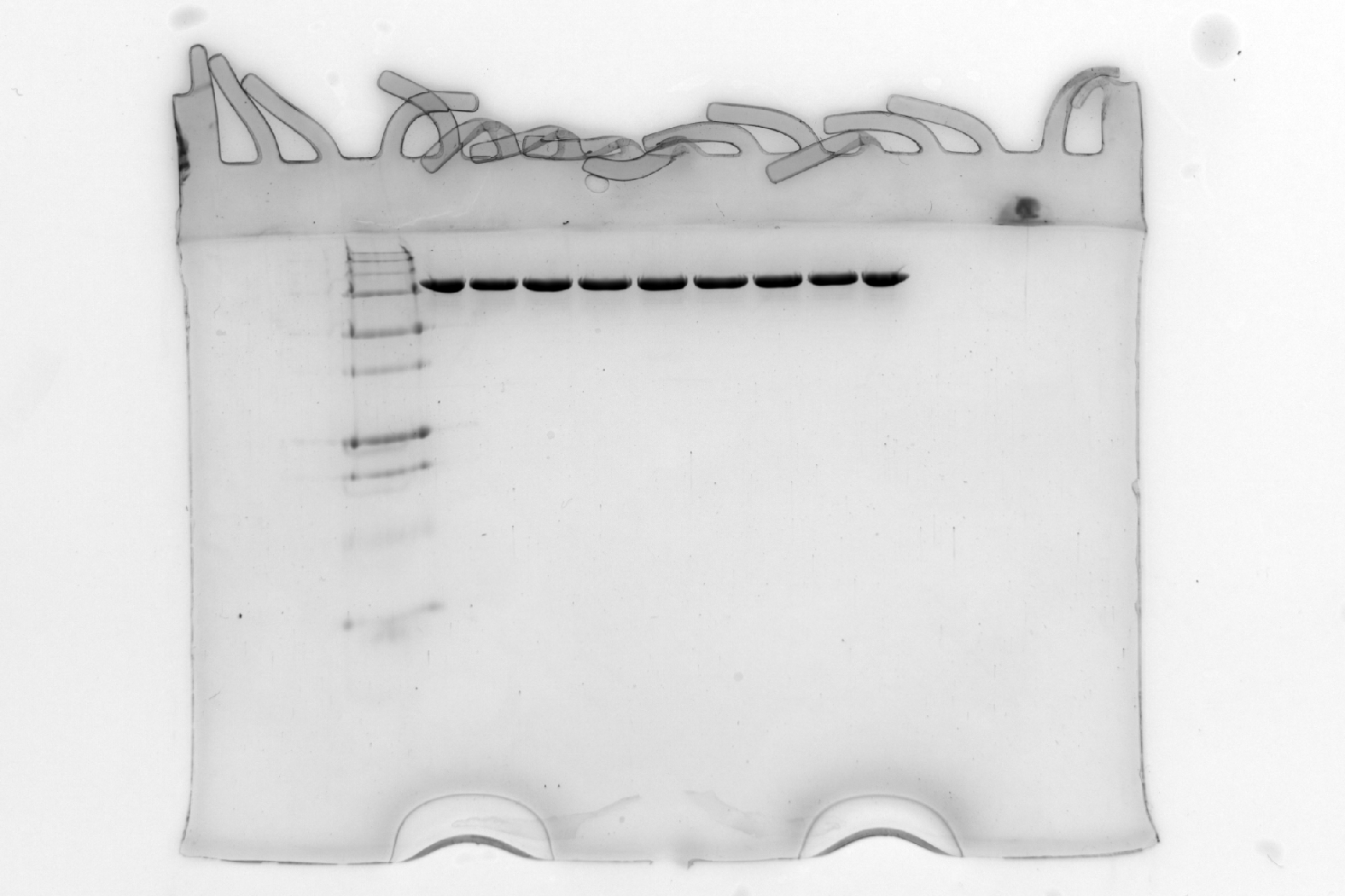

Supplement: Figure 7—figure supplement 1—source data 1. [file elife-88492-fig7-figsupp1-data1.zip › Figure 7 - figure supplement 1 - source data 1/Figure 7ΓÇöfigure supplement 1A middle raw image.jpg]

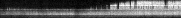

Supplement: Figure 7—figure supplement 1—source data 2. [file elife-88492-fig7-figsupp1-data2.zip › Figure 7 - figure supplement 2 - source data 2/CapZ kymograph #1.tif]

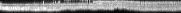

Supplement: Figure 7—figure supplement 1—source data 2. [file elife-88492-fig7-figsupp1-data2.zip › Figure 7 - figure supplement 2 - source data 2/CapZ kymograph #2.tif]

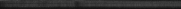

Supplement: Figure 7—figure supplement 1—source data 2. [file elife-88492-fig7-figsupp1-data2.zip › Figure 7 - figure supplement 2 - source data 2/CapZ kymograph #3.tif]

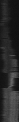

Supplement: Figure 7—figure supplement 1—source data 2. [file elife-88492-fig7-figsupp1-data2.zip › Figure 7 - figure supplement 2 - source data 2/CapZ kymograph #4.tif]

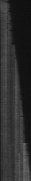

Supplement: Figure 7—figure supplement 1—source data 2. [file elife-88492-fig7-figsupp1-data2.zip › Figure 7 - figure supplement 2 - source data 2/FFICD rap kymograph #1.tif]

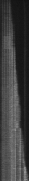

Supplement: Figure 7—figure supplement 1—source data 2. [file elife-88492-fig7-figsupp1-data2.zip › Figure 7 - figure supplement 2 - source data 2/FFICD rap kymograph #2.tif]

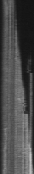

Supplement: Figure 7—figure supplement 1—source data 2. [file elife-88492-fig7-figsupp1-data2.zip › Figure 7 - figure supplement 2 - source data 2/FFICD rap kymograph #6.tif]

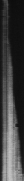

Supplement: Figure 7—figure supplement 1—source data 2. [file elife-88492-fig7-figsupp1-data2.zip › Figure 7 - figure supplement 2 - source data 2/FFICD rap kymograph #4.tif]

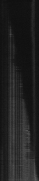

Supplement: Figure 7—figure supplement 1—source data 2. [file elife-88492-fig7-figsupp1-data2.zip › Figure 7 - figure supplement 2 - source data 2/Actin kymograph #5.tif]

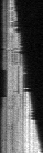

Supplement: Figure 7—figure supplement 1—source data 2. [file elife-88492-fig7-figsupp1-data2.zip › Figure 7 - figure supplement 2 - source data 2/Actin kymograph #4.tif]

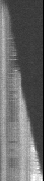

Supplement: Figure 7—figure supplement 1—source data 2. [file elife-88492-fig7-figsupp1-data2.zip › Figure 7 - figure supplement 2 - source data 2/Actin kymograph #3.tif]

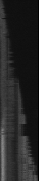

Supplement: Figure 7—figure supplement 1—source data 2. [file elife-88492-fig7-figsupp1-data2.zip › Figure 7 - figure supplement 2 - source data 2/Actin kymograph #1.tif]

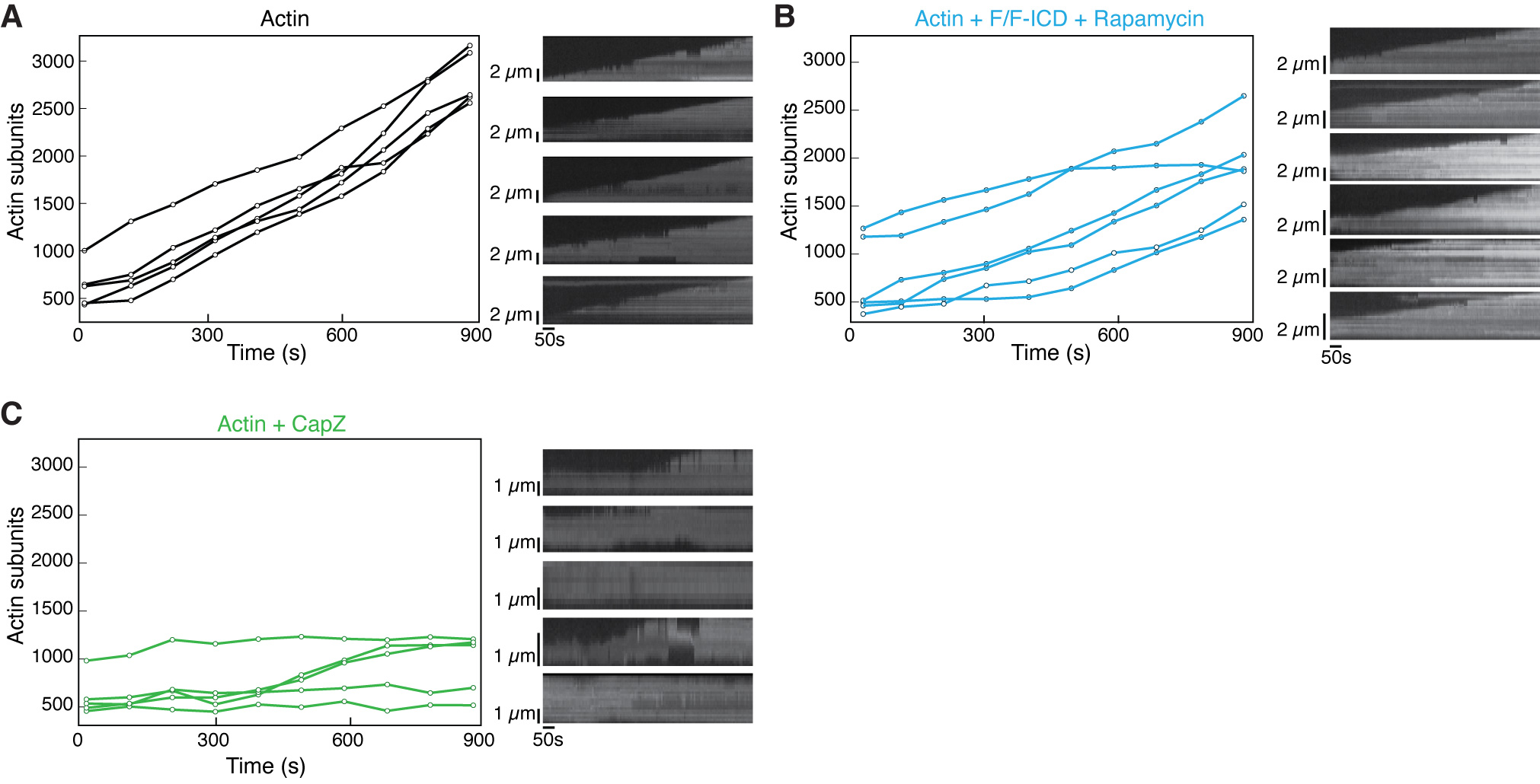

Supplement: Figure 7—figure supplement 2—source data 1. [file elife-88492-fig7-figsupp2-data1.zip › Figure 7 - figure supplement 2 - source data 1/Figure 7 - figure supplement 2 - unedited.jpg]
